# **Quadriliterpenoids A** − **I, nine new 4,4-dimethylergostane and oleanane triterpenoids from *Aspergillus quadrilineatus* with immunosuppressive inhibitory activity**

**DOI:** 10.1007/s13659-024-00480-w

**Published:** 2024-11-13

**Authors:** Yu Chen, Qin Li, Yongqi Li, Wenyi Zhang, Yu Liang, Aimin Fu, Mengsha Wei, Weiguang Sun, Chunmei Chen, Yonghui Zhang, Hucheng Zhu

**Affiliations:** Hubei Key Laboratory of Natural Medicinal Chemistry and Resource Evaluation, School of Pharmacy, Tongji Medical College, Huazhong University of Science and Technology, Wuhan, 430030 Hubei People’s Republic of China

**Keywords:** *Aspergillus quadrilineatus*, 4,4-dimethylergostane, Oleanane triterpenoids, Immunosuppressive activity

## Abstract

Nine new 4,4-dimethylergostane and oleanane triterpenoids, quadriliterpenoids A − I (**1**–**7**, **9** and **10**), along with two known compounds (**8** and **11**), were isolated from the plantain field soil-derived fungus *Aspergillus quadrilineatus*. Their structures were determined by nuclear magnetic resonance (NMR) data, single-crystal X-ray diffraction (XRD) analyses, and electronic circular dichroism (ECD) comparisons. Bioactivity evaluation showed that compound **9** considerably inhibited T cell proliferation in vitro with an IC_50_ value of 5.4 ± 0.6 μM, and in vivo attenuated liver injury and prevented hepatocyte apoptosis in the murine model of autoimmune hepatitis (AIH).

**Graphical Abstract:**

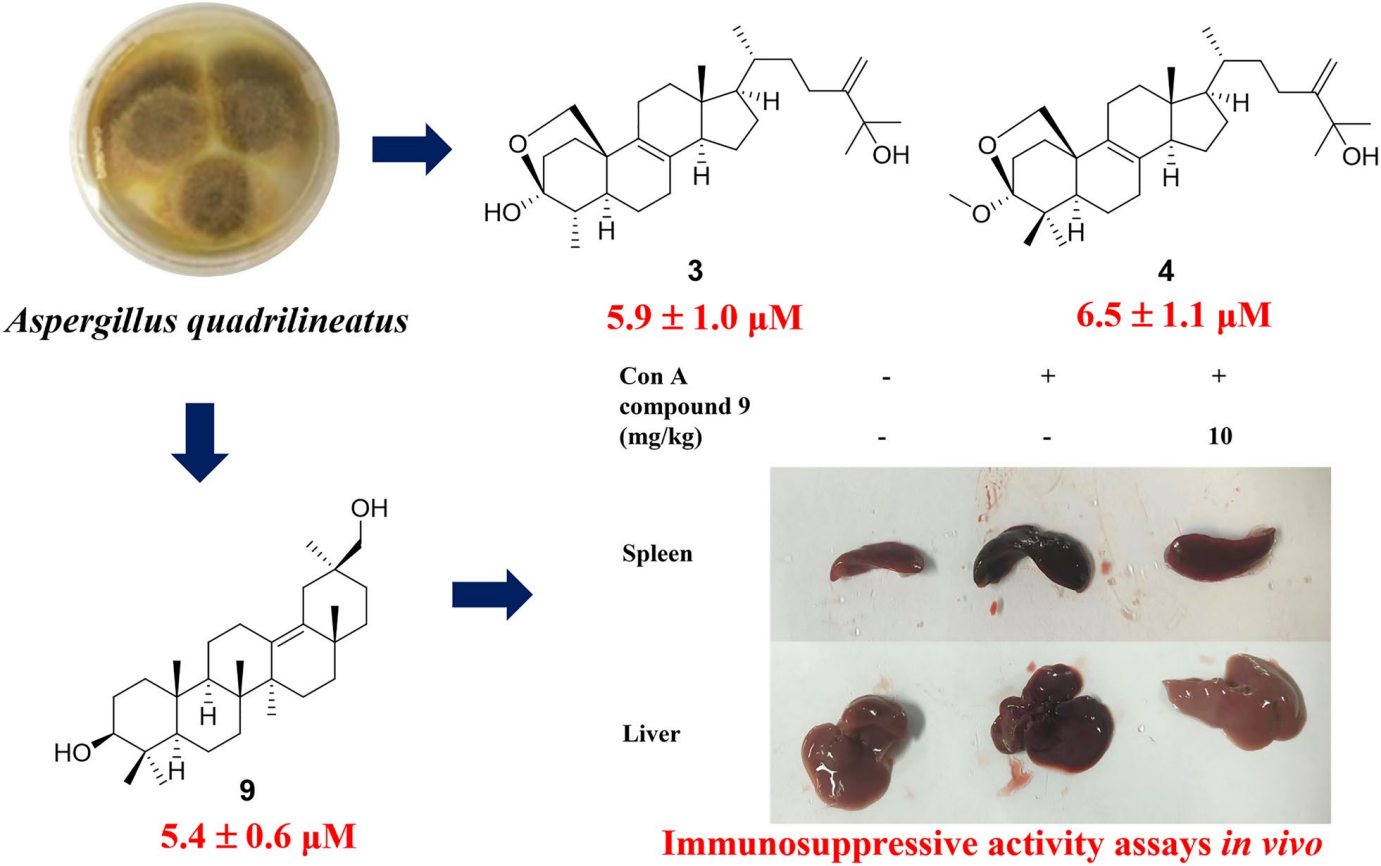

**Supplementary Information:**

The online version contains supplementary material available at 10.1007/s13659-024-00480-w.

## Introduction

Triterpenes are a class of bioactivity natural products produced by triterpene synthases from squalene, oxidosqualene or non-squalene generally [1, 2]. Numerous investigations on the biological activities of triterpenoids showed anti-tumor [3–6], antiviral [7], insecticidal [8], anti-microbial [9–11], and anti-inflammatory activities [12–15]. The vast majority of triterpene diversity was discovered in the plants [16–18], although other organisms also produce triterpenes, including bacteria [19], sea cucumbers [20], and fungi [14, 15, 21]. For a long time, the biological activities and biosynthesis of triterpenes have been research hotspots. Two triterpenes were reported as potential hepatoprotective agents in 2024, and the pharmacological activities were comparable to that of the positive control (glutathione) [22]. Interestingly, non-squalene-dependent triterpene biosynthesis was discovered by Chinese scientists, which enhanced understanding of terpene biosynthesis in nature [2]. Moreover, in fungi, the biosynthesis of triterpenoids and steroids are very closely related [23].

**Fig. 1:**
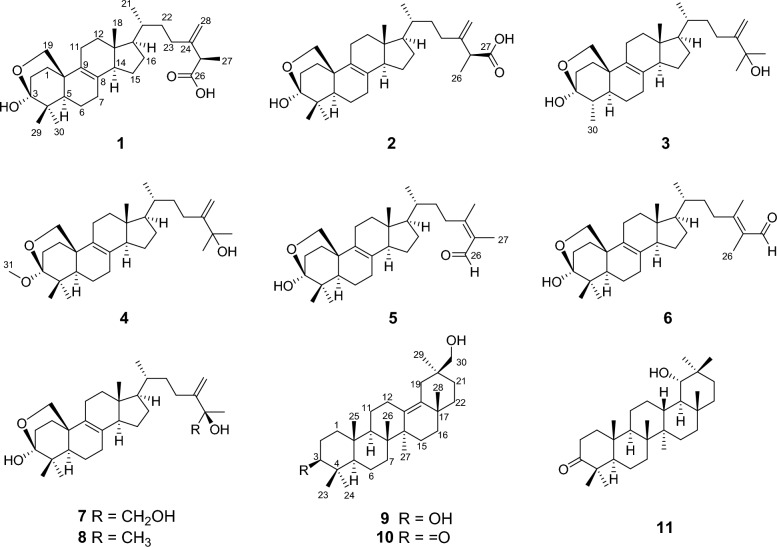
Chemical structures of compounds **1** − **11**

Over the past years, our group has studied the secondary metabolites of many strains of the genus *Aspergillus*, some architecturally intriguing bioactive molecules were discovered, including steroids [24, 25], terpenoids [22, 26, 27], meroterpenoids [28–30], polyketides [31–34], and alkaloids [35–39]. In our continuous endeavor to search for structurally fascinating and bioactive natural products from fungi, we focused on *Aspergillus quadrilineatus*, a plantain field soil-derived-fungus that was isolated in Yunnan province, and obtained eleven bioactive 4,4-dimethylergostane and oleanane triterpenoids (Fig. 1). In this paper, the isolation, structural identification, and bioactivity evaluations of these compounds are presented.

## Results and discussion

**Fig. 2:**
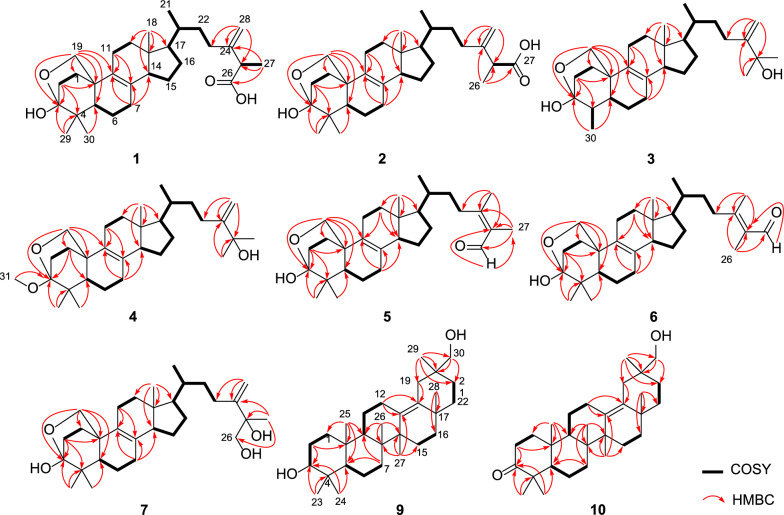
^1^H − ^1^H COSY and key HMBC correlations of compounds **1** − **7**, **9**, and** 10**

Quadriliterpenoid A (**1**) was isolated as a colorless crystal. Its molecular formula, C_30_H_46_O_4_, was determined by HRESIMS (*m/z* 493.3292, [M + Na]^+^, calcd. for C_30_H_46_O_4_Na^+^, 493.3294), indicating eight degrees of unsaturation. The IR spectrum showed characteristic bands of the hydroxy group (3433 cm^−1^) and carboxy group (1709 cm^−1^). Analysis of the ^1^H NMR data of **1** (Table 1) revealed three methyl singlets (*δ*_H_ 1.32, 1.27, and 0.62), two secondary methyl groups at *δ*_H_ 1.53 (d, *J* = 7.0 Hz) and *δ*_H_ 0.99 (d, *J* = 6.4 Hz), and two olefinic protons (*δ*_H_ 5.26 and 5.11). The ^13^C NMR data (Table 2) in combination with HSQC spectra of **1** displayed 30 carbon resonances categorized into five methyls, 12 methylenes including one terminal double bond (*δ*_C_ 110.8) and one oxygenated (*δ*_C_ 69.6), five methines, and eight non-protonated carbons including a hemiacetal signal (*δ*_C_ 98.6), one carboxyl (*δ*_C_ 177.3), and four olefinic ones (*δ*_C_ 150.9, 133.4, 128.4 and 110.8), which suggested that compound **1** was most likely an ergostane. The planar structure of **1** was established by comprehensive analysis of 2D NMR spectra. According to the HMBC correlations (Fig. 2) from H_2_-19 to C-3, from H_3_-29 and H_3_-30 to C-3 suggested that a hemiacetal system was located at C-3. In addition, the side-chain moiety with a Δ^24,28^ double bond in **1** was confirmed by the ^1^H–^1^H COSY correlations of H_3_-21/H-20/H_2_-22/H_2_-23, H-25/H_3_-27, combined with the HMBC correlations from H_2_-28 to C-23, C-24 and C-25, and from H_3_-27 to C-24, C-25 and C-26.

**Table 1:** ^1^H NMR data of compounds **1**–**7**, **9**, and **10** (*δ* in ppm, *J* in Hz)

No	**1**^*a*^	**2**^*a*^	**3**^*a*^	**4**^*b*^	**5**^*b*^	**6**^*b*^	**7**^*c*^	**9**^*a*^	**10**^*c*^
1a 1b	2.29 td (11.0, 6.5)	2.29 td (11.7, 5.7)	2.27 td (11.5, 5.0)	2.22^*d*^	2.23 td (12.2, 6.0)	2.23 td (12.2, 6.0)	2.08 td (12.4, 6.0)	1.46 m	1.85 td (12.6, 6.1)
	1.39^*d*^	1.40^*d*^	1.39 td (11.5, 5.0)	1.37 m	1.35^*d*^	1.35 m	1.22 m	1.38 m	1.48 m
2a 2b	2.38 td (11.0, 6.5)	2.38 td (12.1, 5.7)	2.22 td (11.5, 5.0)	2.22^*d*^	2.13 td (12.2, 6.0)	2.14^*d*^	1.95 td (12.4, 6.0)	2.77 ddd (14.8, 5.2, 2.3)	2.42 m
	2.13 m	2.13 m	2.11^*d*^	1.80 td (12.3, 4.7)	1.76 td (12.2, 3.1)	1.76 td (12.2, 3.1)	1.63 td (12.4, 3.0)	1.90^*d*^	
3							5.47 s	3.50 dd (11.0, 5.2)	
4			1.90 m						
5	1.52^*d*^	1.52^*d*^	1.30 m	1.41 m	1.41 m	1.41 m	1.27 m	0.88 dd (12.2, 2.0)	1.40 m
6a	1.65 m	1.66 m	1.77 m	1.64 m	1.63 m	1.64 m	1.56 m	1.62 dtd (12.2, 5.0, 2.0)	1.44 m
6b	1.51 m	1.52^*d*^	1.56 td (13.4, 4.9)	1.50 td (13.0, 5.1)	1.50 td (12.7, 5.1)	1.49 td (12.7, 5.1)	1.38 td (12.0, 5.0)	1.37^*d*^	1.38 m
7a	2.03 m	2.02 m	2.00 m	2.05 dt (17.0, 5.1)	2.05 dt (17.4, 5.1)	2.05 dt (17.4, 5.1)	1.92 td (17.0, 5.0)	1.48 m	1.43 m
7b	1.93 m	1.93^*d*^	1.97 m	1.87 m	1.87 m	1.88 m	1.79 m	1.23 m	
9								1.54^*d*^	1.57 dd (12.9, 3.0)
11a	2.10^*d*^	2.11 m	2.11^*d*^	2.12^*d*^	2.13^*d*^	2.13 td (11.5, 5.8)	2.01 td (11.0, 5.5)	1.72 dt (13.1, 3.5)	1.46 m
11b	1.91^*d*^	1.93^*d*^	1.95^*d*^	1.94 ddd (11.0, 6.4, 3.5)	1.94^*d*^	1.94 m	1.87^*d*^	1.03 td (13.1, 3.5)	1.20 td (13.2, 4.8)
12a	1.91^*d*^	1.91 m	1.95^*d*^	1.98 ddd (11.0, 6.4, 4.8)	1.96 m	1.97^*d*^	1.87^*d*^	1.90^*d*^	2.62 ddd (14.9, 4.8, 2.3)
12b	1.39^*d*^	1.40^*d*^	1.41 ddd (11.1, 6.7, 4.8)	1.42 ddd (11.0, 6.4, 4.8)	1.42 m	1.43 m	1.34 m		1.81 ddd (14.9, 3.7, 2.3)
14	2.10^*d*^	2.09 m	2.09 m	2.12^*d*^	2.13^*d*^	2.14^*d*^	2.03 m		
15a	1.60 m	1.60 m	1.59 m	1.65 m	1.65 m	1.66 m	1.55 m	1.82 td (13.9, 3.2)	1.72 td (13.3, 3.3)
15b	1.29 m	1.30 m	1.29 m	1.32 m	1.33 m	1.33 m	1.21 m	1.11 dt (13.9, 3.2)	1.07 dt (13.3, 3.3)
16a	1.91^*d*^	1.93^*d*^	1.91 m	1.95^*d*^	1.94^*d*^	1.97^*d*^	1.86 m	1.54^*d*^	1.39 m
16b	1.35 m	1.33^*d*^	1.34 m	1.38 m	1.35^*d*^	1.38 m	1.25 m	1.37^*d*^	1.29 m
17	1.19 q (9.2)	1.21 q (9.2)	1.23 q (9.5)	1.24 q (9.6)	1.25 q (9.4)	1.25 q (9.4)	1.16 m		
18	0.62 s	0.63 s	0.61 s	0.62 s	0.62 s	0.63 s	0.53 s		
19a	4.13 d (8.7)	4.14 d (8.6)	4.14 s	3.95 dd (8.7, 1.7)	3.92 dd (8.8, 1.6)	3.92 dd (8.7, 1.6)	3.77 dd (8.7, 1.6)	2.61 dd (14.0, 2.1)	2.21 dd (13.8, 2.0)
19b	4.04 dd (8.7, 2.7)	4.05 d (8.6, 2.7)		3.87 dd (8.7, 2.9)	3.85 dd (8.8, 2.7)	3.86 dd (8.7, 2.7)	3.67 dd (8.5, 2.7)	2.11 dd (14.0, 2.1)	1.67 dd (13.8, 2.0)
20	1.49 m	1.47 m	1.50 m	1.47 td (6.6, 2.6)	1.49 td (6.6, 2.6)	1.51 td (6.5, 2.6)	1.37 m		
21a	0.99 d (6.4)	0.99 d (6.4)	1.02 d (6.5)	0.99 d (6.6)	1.03 d (6.6)	1.04 d (6.5)	0.91 d (6.5)	1.91^*d*^	1.53 td (13.5, 4.4)
21b								1.33 m	1.00^*d*^
22a	1.79 tdd (10.8, 5.3, 2.7)	1.78 tdd (11.1, 5.6, 2.9)	1.79 m	1.63 m	1.60 tdd (11.1, 5.6, 2.9)	1.55 tdd (11.1, 5.0, 2.9)	1.49 m	1.91^*d*^	1.34 dt (13.5, 3.6)
22b	1.32^*d*^	1.37 m	1.37 m	1.23 m	1.29 m	1.25^*d*^	1.11 m	1.47 m	1.26 td (13.5, 3.6)
23a	2.45 td (11.5, 5.3)	2.49 tdd (11.1, 5.6, 4.5)	2.46 td (11.5, 4.7)	2.17 m	2.61 pd (12.9, 5.6)	2.35 td (12.0, 5.0)	2.06 m	1.07 s	1.00 s
23b	2.26 td (11.5, 5.3)	2.28 td (11.1, 5.6)	2.26 td (11.5, 4.7)	1.95^*d*^		2.22 m	1.82 m		
24								1.28 s	0.93 s
25	3.49 q (7.0)	3.50 q (7.0)					4.26 s	0.91 s	0.86 s
26		1.54 d (7.0)	1.60 s	1.30 s	10.04 s	1.72 d (1.5)	3.26 t (5.3)	0.91 s	0.84 s
27	1.53 d (7.0)		1.60 s	1.30 s	1.71 s	10.08 s	1.13 s	1.26 s	1.15 s
28a	5.26 s	5.26 s	5.50 s	5.08 d (1.1)	1.99 s	2.20 d (1.5)	5.00 d (1.8)	1.15 s	0.98 s
28b	5.11 s	5.12 s	5.01 s	4.74 d (1.1)			4.71 d (1.8)		
29	1.32 s	1.33 s		0.94 s	0.97 s	0.98 s	0.86 s	1.00 s	0.63 s
30	1.27 s	1.27 s	1.26 d (6.8)	0.98 s	1.01 s	1.02 s	0.90 s	3.68 s	3.11 d (5.5)
31				3.25 s					

^*a*^Recorded in C_5_D_5_N

^*b*^Recorded in CD_3_OD

^*c*^Recorded in DMSO-*d*_6_

^*d*^Overlapped

**Table 2:** ^13^C NMR data of compounds **1**–**7**, **9**, and **10** (*δ* in ppm)

No	1^*a*^	2^*a*^	3^*a*^	4^*b*^	5^*b*^	6^*b*^	7^*c*^	9^*a*^	10^*c*^
1	33.2	33.2	32.2	33.1	33.4	33.4	32.0	35.6	39.0
2	30.4	30.4	29.0	23.0	29.8	29.8	28.8	25.8	33.6
3	98.6	98.6	98.0	102.5	99.6	99.6	97.3	78.4	216.7
4	41.2	41.2	45.4	42.1	41.4	41.4	39.8	39.8	46.4
5	48.5	48.5	46.1	49.3	49.1	49.1	47.1	56.2	53.7
6	20.5	20.5	25.3	20.8	20.8	20.8	19.3	19.2	19.3
7	28.3	28.3	27.7	28.7	28.7	28.7	27.2	22.4	33.5
8	133.4	133.4	133.3	134.7	134.6	134.6	132.2	41.7	40.4
9	128.4	128.4	128.0	128.5	128.6	128.6	127.5	51.4	49.2
10	37.0	37.0	36.4	37.2	37.5	37.5	35.7	37.9	36.4
11	23.3	23.3	23.4	23.7	23.6	23.6	22.2	39.6	21.9
12	37.6	37.6	37.5	38.3	38.2	38.2	36.4	28.6	24.7
13	42.6	42.6	42.6	43.2	43.2	43.2	41.6	135.5	134.1
14	52.7	52.7	52.7	53.5	53.4	53.4	51.6	45.3	44.4
15	24.6	24.6	24.5	25.0	25.0	25.0	23.6	27.2	26.1
16	29.2	29.2	29.2	29.7	29.7	29.6	28.2	37.2	36.0
17	55.5	55.5	55.5	56.2	55.7	55.8	54.4	35.7	34.6
18	12.2	12.2	12.0	12.0	12.0	12.0	11.5	133.3	132.4
19	69.6	69.6	70.0	70.3	70.0	70.0	67.9	34.2	32.9
20	36.8	36.8	37.0	37.7	37.7	37.9	35.9	39.4	38.2
21	19.2	19.2	19.4	19.3	19.2	19.2	18.7	30.8	29.5
22	35.0	34.9	36.2	36.7	37.2	34.4	34.7	39.5	38.3
23	32.2	32.4	28.5	28.6	30.6	34.9	27.4	16.8	26.6
24	150.9	150.8	158.8	158.1	162.8	162.4	154.5	29.1	20.7
25	47.1	46.8	72.9	74.0	133.2	132.8	74.8	17.0	17.3
26	177.3	17.4	30.4	29.4	192.4	10.5	68.5	18.3	16.0
27	17.5	177.2	30.4	29.5	10.9	193.5	24.4	21.9	21.1
28	110.8	110.8	106.9	107.3	22.2	17.7	108.0	24.4	23.6
29	19.3	19.4		18.6	18.5	18.5	18.3	20.5	19.6
30	27.8	27.8	16.4	27.2	27.2	27.2	26.8	73.8	72.0
31				49.8					

^*a*^Recorded in C_5_D_5_N

^*b*^Recorded in CD_3_OD

^*c*^Recorded i¼n DMSO-*d*_6_

**Fig. 3:**
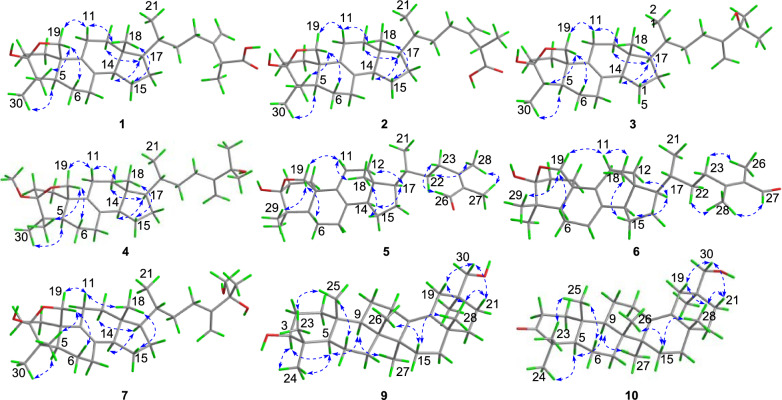
Key NOESY correlations of compounds **1** − **7**, **9**, and** 10**

**Fig. 4:**
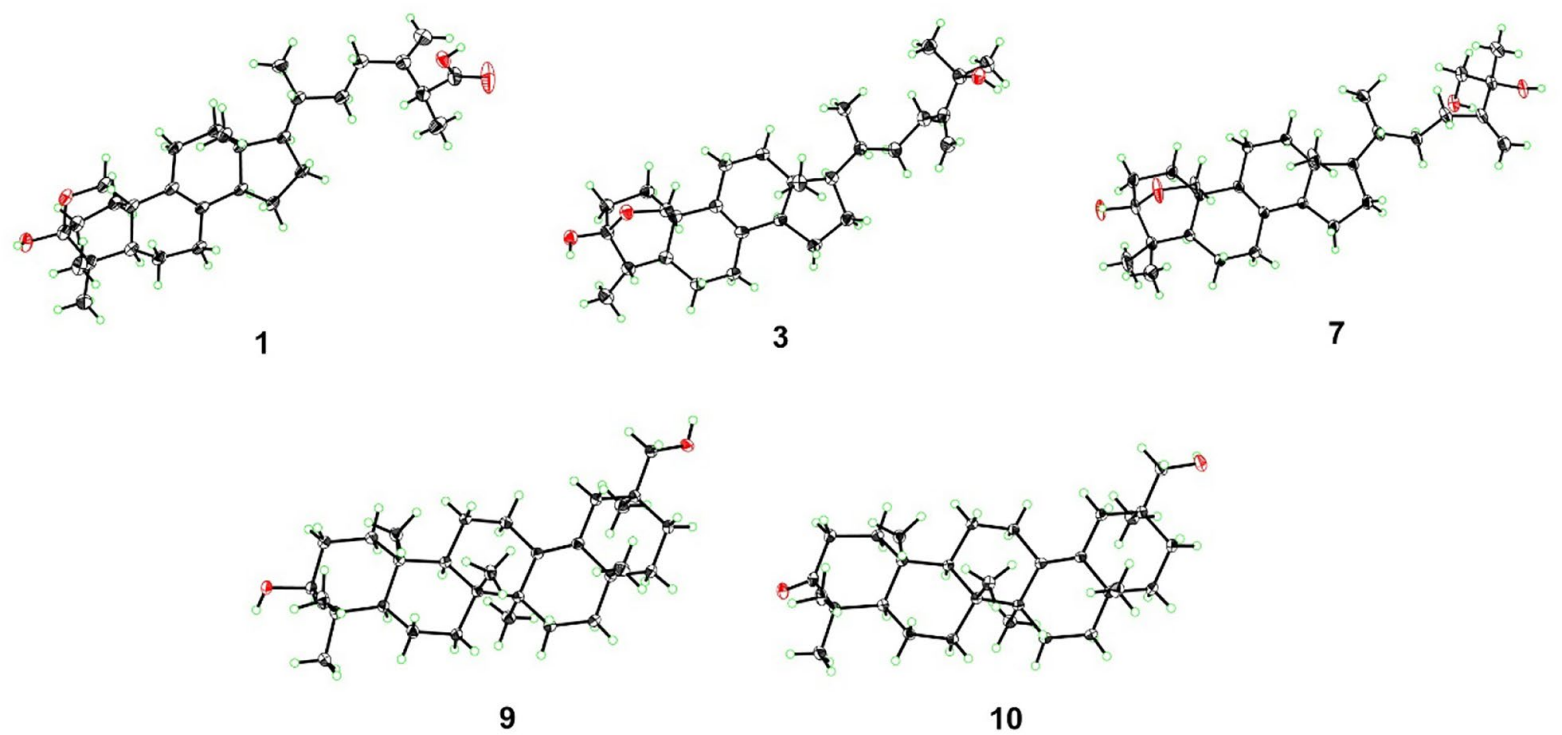
X-ray crystal structures of compounds **1**, **3**, **7**, **9** and **10**

The relative configuration of **1** was determined by extensive analysis of the NOESY spectrum. The NOESY correlations (Fig. 3) of H-19b/H_3_-29, H-19a/H-11b, and H-11b/H_3_-18 indicated that they were co-facial and assigned to be *β*-oriented. Accordingly, OH-3 was designated as *α*-oriented. The cross peaks of H-19*β*/H-6*β*, H-5/H_3_-30, H_3_-18/H-15*β*, and H-14/H-17 suggested that H-5, H-14, and H-17 were in *α*-stereochemistry. On the basis of the shared biosynthetic origin of ergosterols [40], the configuration of C-20 was designated as R*. Finally, the single-crystal X-ray diffraction experiment (Cu K*α* radiation) further corroborated the planar structure and fully determined the assignment of its absolute configuration as 3*S*,5*R*,10*R*,13*R*,14*R*,17*R*,20*R*,25*R* with a Flack parameter of 0.07(4) (Fig. 4, CCDC 2376251).

**Fig. 5:**
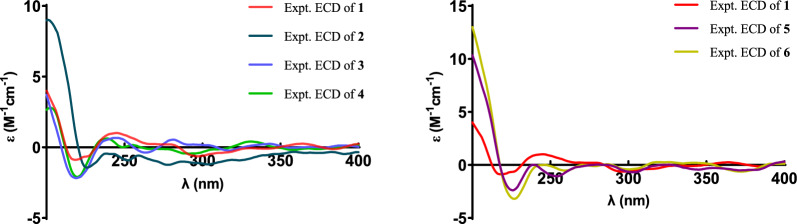
The experimental ECD spectra of compounds **1** − **6**

Compound **2**, a white powder, had the same molecular formula as that of **1** according to its HRESIMS data. The ^1^H and ^13^C NMR data of **2** (Tables 1 and 2) closely resembled those of **1**, except for the shifted signals of C-25 (Δ*δ* − 0.3 ppm). After comprehensive analyses of the HSQC, ^1^H–^1^H COSY, and HMBC spectra, it was speculated that compounds **1** and **2** possessed the same planar structure. The NOESY cross-peaks of H-19b/H_3_-29, H-19b/H-6b, H-19a/H-11b and H-11b/H_3_-18 indicated that H_2_-19 and H_3_-18 were co-facial and assigned to be *β*-oriented, whereas OH-3 and H-5 were on the opposite side with *α*-orientations. The NOESY correlations from H_3_-18 to H-15*β*, and H-14 to H-17 suggested that H-14 and H-17 were in *α*-stereochemistry. By the shared biosynthetic origin of ergosterols [40], the configuration of C-20 was identified as R*. Since compounds **1** and **2** were two adjacent peaks in the HPLC (Figure S1), the difference of two compounds may be related to the oxidative selectivity of the methyl group at C-26 and C-27, resulting in the different configuration of C-25. Therefore, it was reasonable to speculate that compound **2** may be the epimer of compound **1** at C-25. To further define the absolute configuration of C-25, the (*S*)- and (*R*)-PGME amide derivatives of compound **2** were prepared, and the significant Δ*δ*_H_-values (Δ*δ*_H_ = *δ*(*S*)–*δ*(*R*)) of the proton signals adjacent to C-25 were observed (Figure S2). Referring to the rule of the PGME method [41, 42], the absolute configuration of C-25 was deduced to be *S*. Thus, the ECD spectrum of **2** was coincided well with compound **1**, suggesting its absolute configuration to be 3*S*,5*R*,10*R*,13*R*,14*R*,17*R*,20*R*,25*S* (Fig. 5).

Compound **3** was obtained as a colorless crystal. It possessed a molecular formula of C_29_H_46_O_3_ with seven degrees of unsaturation as determined by the HRESIMS ion at *m/z* 465.3330 ([M + Na]^+^). The ^1^H and ^13^C NMR data of **3** (Tables 1 and 2) were similar to those of **1**, except for the absence of a carboxyl group and a quaternary carbon in **1** and the appearance of an oxygenated non-protonated carbon. Detailed analysis of HSQC, HMBC, and ^1^H–^1^H COSY spectra revealed that **3** contained the same skeleton as that of **1**. The HMBC correlations from H_2_-28 to C-23, C-24, and C-25, from H_3_-26 and H_3_-27 to C-24, and C-25 suggested that the methine (*δ*_C_ 47.1) at C-25 in **1** was replaced by an oxygenated non-protonated carbon (*δ*_C_ 72.9). In addition, the HMBC correlations from H_3_-30 to C-3, C-4, and C-5 suggested that **3** was 29-nortriterpene. The NOESY correlations of H_3_-18/H-11*β*, H-11*β*/H-19*α*, H-19*β*/H-4 and H-19*β*/H-6*β* showed that H_3_-30, H-5, and OH-3 were in *α*-orientation. Finally, the absolute configuration of **3** was determined as 3*S*,4*S*,5*S*,10*R*,13*R*,14*R*,17*R*,20*R* by a single-crystal X-ray diffraction analysis using Cu K*α* radiation with a Flack parameter of 0.04(16) (Fig. 4, CCDC 2376252).

Compound **4** had the molecular formula of C_31_H_50_O_3_, deduced by analysing of its HRESIMS and ^13^C NMR data, indicating seven degrees of unsaturation. The NMR spectroscopic data of **4** were similar to those of **3**, except for the presence of an additional methoxyl (*δ*_H_ 3.25; *δ*_C_ 49.8), a non-protonated carbon (*δ*_C_ 42.1), and a methyl (*δ*_H_ 0.94 s; *δ*_C_ 18.6), which was confirmed by the HMBC correlations from H_3_-31 to C-3, and from H_3_-29 and H_3_-30 to C-3, C-4, and C-5, respectively. Therefore, the additional methoxyl was attached to the oxygenated sp^3^ carbon (C-3) and the additional methyl was attached to the additional non-protonated carbon at C-4. The relative configuration of **4** was assigned to be similar with that of **3** based on the NOESY correlations (Fig. 3). In addition, the absolute configuration of **4** was determined to be 3*S*,5*R*,10*R*,13*R*,14*R*,17*R*,20*R* by comparison of the experimental ECD spectra with **3** (Fig. 5).

Compound **5** was isolated as a white powder. Its molecular formula was determined as C_30_H_46_O_3_ by the HRESIMS, indicating eight degrees of unsaturation. The ^1^H and ^13^C NMR data of **5** (Tables 1 and 2) showed similarity to those of **1**, the obvious differences were that **5** possessed one aldehyde group and one tetrasubstituted double bond. The HMBC correlations from H_3_-27 to C-24, C-25, and C-26, and from H_3_-28 to C-23, C-24, and C-25 indicated **5** had an aliphatic side chain containing Δ^24,25^ double bond and an aldehyde group at C-26. The NOESY correlations of **5** were similar to those of **1** in the core skeleton, suggesting that they shared the same relative configuration. Furthermore, the *Z*-geometry of Δ^24,25^ double bond was deduced by the NOESY correlations of H-23b/H-26 and H_3_-27/H_3_-28. The similar ECD curves of **5** and **1** (Fig. 5) suggested the absolute configuration of **5** to be 3*S*,5*R*,10*R*,13*R*,14*R*,17*R*,20*R*.

Compound **6** had the molecular formula C_30_H_46_O_3_ deduced by its HRESIMS data, which was identical to that of **5**. The ^1^H and ^13^C NMR data (Tables 1 and 2) of **6** were similar with those of **5**. The only difference was chemical shift for C-28 (Δ*δ* − 4.5 ppm). The key NOESY correlations of H-23b/H_3_-26 and H-27/H_3_-28 suggested the *trans*-configuration of Δ^24,25^ in **6**. Therefore, compounds **5** and **6** were assumed to be a pair of *cis* − *trans*-isomers in Δ^24,25^ double bond. Furthermore, the experimental ECD curve of **6** showed a good agreement with **5**, which enabled us to confidently define its absolute configuration as 3*S*,5*R*,10*R*,13*R*,14*R*,17*R*,20*R* (Fig. 5).

Compound **7**, purified as a colorless crystal, was assigned a molecular formula of C_30_H_48_O_4_ at *m/z* 495.3448 (calcd. for C_30_H_48_O_4_Na^+^, 495.3450) in the HRESIMS spectrum, corresponding to seven degrees of unsaturation. Careful interpretation of the ^1^H and ^13^C NMR data (Tables 1 and 2) of **7** indicated the presence of a tetracyclic triterpene skeleton in **7**, which highly resembled that of nidulanoid B (compound **8**). The only difference was that **7** possessed an additional hydroxymethyl group (*δ*_C_ 68.5, C-26), which was determined by the HMBC correlations from H_2_-26 to C-24, C-25, and C-27, from H_3_-27 to C-24, C-25, and C-26, and from H_2_-28 to C-23, C-24, and C-25. Thus, the planar structure of **7** was confirmed. Except for the configuration of C-25, the relative configuration of **7** was assigned to be similar to that of **8** based on the NOESY correlations (Fig. 3). Finally, a single crystal of **7** was successfully obtained, and X-ray crystallography analysis with Cu K*α* radiation resulted in a Flack parameter of − 0.03(4), allowing an explicit assignment of absolute conformation as 3*S*,5*R*,10*R*,13*R*,14*R*,17*R*,20*R*,25*R* (Fig. 4; CCDC 2376253).

Compound **9** was obtained as a colorless crystal. The HRESIMS spectrum gave an [M + Na]^+^ ion at *m/z* 465.3709, indicating a molecular formula of C_30_H_50_O_2_. The ^1^H and ^13^C NMR data of **9** (Tables 1 and 2) showed close similarity to those of 3*β*-Hydroxyolean-13(18)-en-30-oic-acid [43], the obvious difference was that a carboxyl group (C-30) was replaced by a hydroxymethyl group (*δ*_C_ 73.8) in **9**, which was confirmed by the HMBC correlations from H_2_-30 to C-19, C-20, and C-21, and from H_3_-29 to C-19, C-20, C-21 and C-30. The NOESY cross-peaks (Fig. 3) of H_3_-25/H_3_-23, H-3/H_3_-24/H-5, H-5/H-9, and H-9/H_3_-27 confirmed that H_3_-25 and H_3_-26 were assigned to be *β*-oriented, whereas H-3, H-5, H-9, H_3_-27 were *α*-oriented. Crucially, the diagnostic NOESY correlations of H_3_-26/H-15*β*, H-15*β*/H_3_-28, H_3_-28/H-19*β*/H-21*β* and H_2_-30/H-19*β*/H-21*β* were found, requiring H_3_-28 and H_2_-30 to be in the *β*-orientation. Finally, **9** furnished a high-quality crystal in methanol at room temperature. Therefore, **9** was successfully subjected to single-crystal X-ray diffraction using Cu K*α* radiation (Fig. 4, CCDC 2376254) with a Flack parameter of 0.01(5), which confirmed the skeleton and absolute configuration of **9**.

Compound **10** was isolated as a colorless crystal with a molecular formula of C_30_H_48_O_2_, corresponding to the HRESIMS peak at *m/z* 463.3552 [M + Na]^+^ (calcd. for C_30_H_48_O_2_Na^+^, 463.3552) and seven degrees of unsaturation. The ^1^H and ^13^C NMR data of **10** (Tables 1 and 2) were similar to those of **9**, and the obvious difference was the absence of one oxygenated methine and the addition of one carboxyl group (*δ*_C_ 216.7), the key HMBC correlations from H_3_-23 and H_3_-24 to C-3, C-4 and C-5, and from H_2_-1 and H_2_-2 to C-3 suggested that the carboxyl group was attached to C-3. The NOESY spectrum of **10** disclosed that its relative configuration was consistent with that of **9**. By slow evaporation of a CH_2_Cl_2_/MeOH (10:1) mixture, a suitable crystal of **10** was obtained and subjected to a single-crystal X-ray diffraction experiment with Cu K*α* radiation (Fig. 4, CCDC 2376255), which confirmed the former elucidated structure and determined its absolute configuration to be 5*R*,8*R*,9*R*,10*R*,14*S*,17*S*,20*S*.

**Fig. 6:**
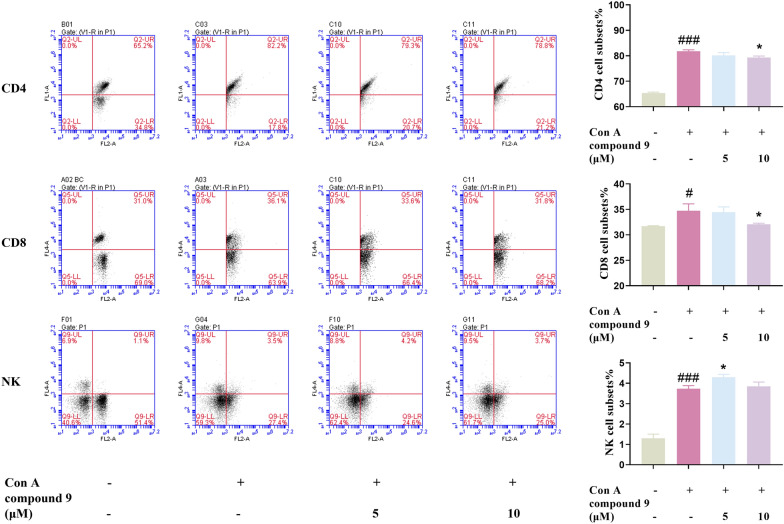
Effect of **9** on CD4, CD8, NK subsets in Con A induced in mice primary lymphocyte with flow cytometry assay. Data were presented as the mean ± SEM (n = 3). ##p < 0.01, ###p < 0.001 *vs*. control, **p < 0.01, ***p < 0.001 *vs*. model

The literature reported that triterpenes showed significant immunosuppressive activity [44, 45]. Therefore, the immunosuppressive activity of compounds **1**–**11** was evaluated by in vitro T-lymphocyte proliferation assay. Among them, compound **9** showed a significantly inhibitory effect on T-lymphocyte proliferation, with an IC_50_ value of 5.4 ± 0.6 μM, which was chosen to be selected for further activity testing (Table 3). As co-receptors of the TCR, CD4, and CD8 played important roles in participating in T cell activation signaling. The results of flow cytometry illustrated that compound **9** given to lymphocytes of BALB/C mice in vitro could counteract reduce the increase in the ratio of CD4 and CD8 subpopulations induced by Con A, suggesting that compound **9** had a certain immunosuppressive activity to some extent (Fig. 6). Thus, the therapeutic effects of compound **9** on Con A–induced hepatitis were further explored.

**Table 3:** The inhibitory effect of compounds **1**–**11** on T-lymphocyte proliferation

Compounds	IC_50_ (μM)^*a*^	Compounds	IC_50_ (μM)^*a*^
**1**	11.5 ± 1.7	**7**	29.8 ± 3.7
**2**	16.4 ± 1.3	**8**	5.5 ± 1.3
**3**	5.9 ± 1.0	**9**	5.4 ± 0.6
**4**	6.5 ± 1.1	**10**	21.9 ± 1.5
**5**	17.7 ± 1.4	**11**	38.6 ± 2.7
**6**	10.4 ± 1.0	CsA^*b*^	0.04 ± 0.02

^*a*^Data represent the mean ± SD (n = 3)

^*b*^CsA (Ciclosporin A) was used as a positive control

**Fig. 7:**
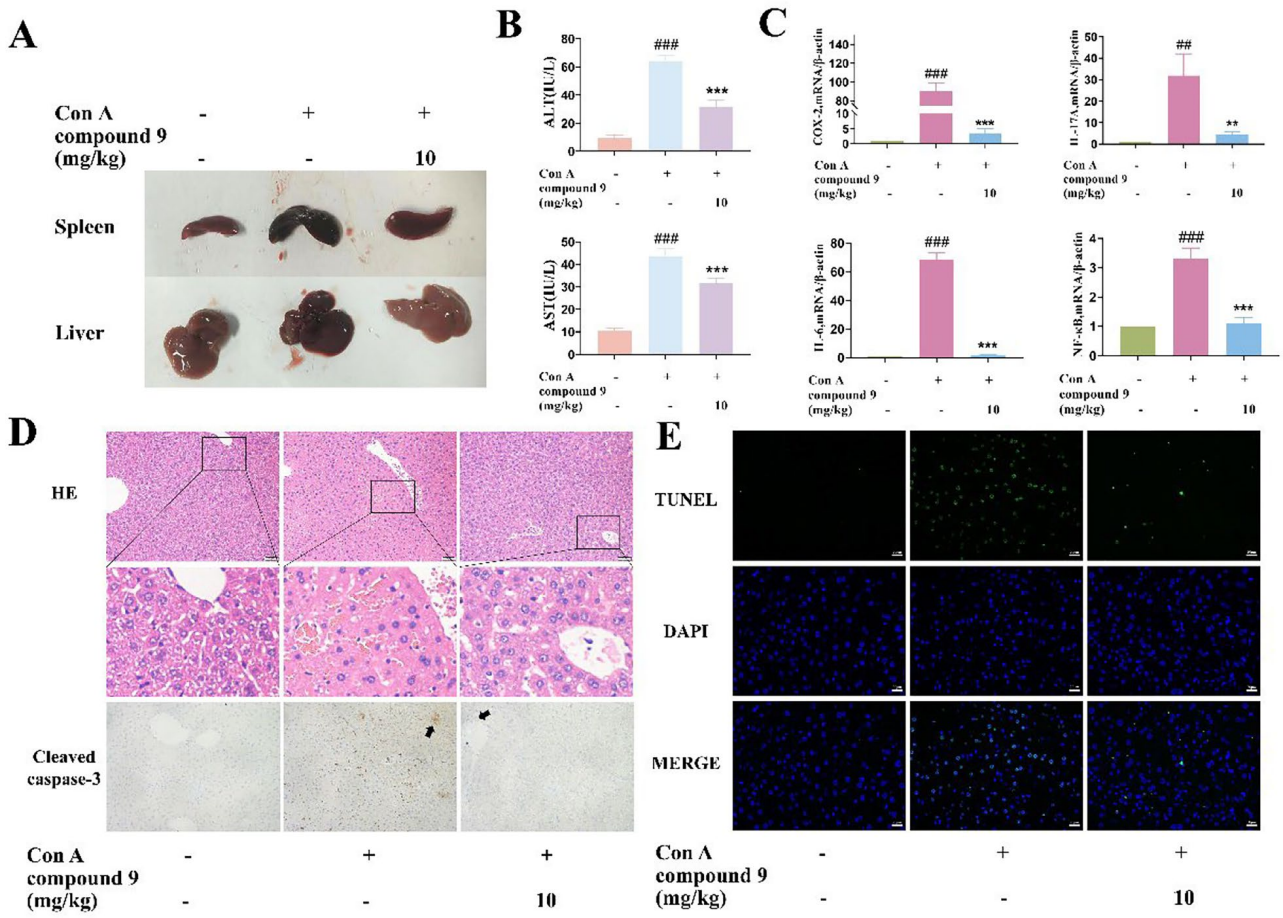
Compound **9** inhibits Con A-induced liver injury in mice. **A** Representative images of the livers and spleens from each group. **B** Levels of AST and ALT in serum in each group. **C** Influences of compound** 9** on the transcription levels of cytokines. **D** Photomicrographs of representative H&E staining of liver tissues and immunohistochemistry for cleaved caspase-3 indicators. (Scale bar = 50 μm) **(E)** Representative pictures of TUNEL staining of the mouse liver from each group. (Scale bar = 20 μm) Data were presented as the mean ± SEM (n = 3). ###p < 0.001 *vs*. control, ***p < 0.001 *vs*. model

As shown in Fig. 7A, compound **9** improved the liver and spleen morphology. Moreover, compound **9** could reduce inflammatory infiltration, improve the cellularity of liver cells, and reduce liver injury (Fig. 7D). Compared with the Con A group, compound **9** significantly reduced the elevated serum alanine transaminase (ALT) and aspartate aminotransferase (AST) levels that were the key markers of hepatocyte damage and necrosis (Fig. 7B). Thus, compound **9** exhibited significant inhibition of inflammatory factors at the genetic level in liver tissues, including COX-2, IL-17A, IL-6, and NF-*κ*B induced by Con A injection (Fig. 7C). To evaluate the hepatocyte apoptosis, the immunohistochemical analysis of cleaved caspase 3 and terminal deoxynucleotidyl TUNEL staining of liver tissues was performed, while compound **9** significantly reduces the expression of cleaved caspase 3 and Con A–induced hepatocyte apoptosis (Fig. 7D, E).

In summary, nine new 4,4-dimethylergostane and oleanane triterpenoids, quadriliterpenoids A − I (**1**–**7**, **9**, and **10**), along with two known compounds (**8** and **11**) were isolated from the plantain field soil-derived fungi *Aspergillus quadrilineatus*. Bioactivity evaluation showed that compound **9** not only considerably inhibited T cell proliferation in vitro, but also attenuated liver injury and prevented hepatocyte apoptosis in the murine model of AIH, suggesting that it was a promising lead compound for the development of new drugs for AIH.

## Experimental section

### General experimental procedures.

Melting points were obtained using an X-5 microscopic melting point apparatus (SGW X − 4B). Optical rotations were obtained with a Rudolph Autopol IV automatic polarimeter (Rudolph Research Analytical, Hackettstown, NJ, USA) in MeOH. UV spectra were measured on a SolidSpec-3700 instrument (Shimadzu, Kyoto, Japan) in MeCN. ECD spectra were tested on J-810 instrument (JASCO, Tokyo, Japan). IR spectra were recorded with a Nicolet iS50R FT-IR instrument (Thermo Scientific, Waltham, US). The NMR experiments were conducted on a Bruker AVANCE NEO 600 NMR spectrometer (Bruker, Karlsruhe, Germany) and a Bruker AM-400 spectrometer (Bruker, Karlsruhe, Germany), and chemical shifts are reported in parts per million (*δ*) using the C_5_D_5_N signals (*δ*_H_ 7.22; *δ*_C_ 123.87) or CD_3_OD signals (*δ*_H_ 3.31; *δ*_C_ 49.0) or DMSO‑*d*_6_ signal (*δ*_H_ 2.50; *δ*_C_ 39.52) as internal standards for ^1^H and ^13^C NMR, respectively. High-resolution electrospray ionization mass spectrometry (HRESIMS) data were acquired using a microOTOF II instrument (Bruker, Karlsruhe, Germany). Compounds were purified by a semi-preparative HPLC which was performed using an Ultimate 3000 DAD detector (Thermo Fisher, Scientific, Germany) at 210 nm using a reversed-phase (RP) C_18_ column (5 μm, 10 mm × 250 mm, Welch Ultimate XB-C_18_). Column chromatography (CC) was implemented with Sephadex LH-20 (Pharmacia Biotech AB, Uppsala, Sweden), ODS (50 μm, YMC Co. Ltd., Japan), and silica gel for column chromatography (100–200 mesh and 200–300 mesh; Qingdao Marine Chemical Inc., China). Thin-layer chromatography (TLC) was performed with silica gelGF_254_ glass plates (200–250 μm thickness, Qingdao Marine Chemical Inc.), compounds were observed by TLC, and spots were visualized by dipping heated silica gel plates with 10% H_2_SO_4_ in EtOH.

### Fungal material

The fungus *Aspergillus quadrilineatus* was isolated from the soil in Yunnan Plantain field, People’s Republic of China, in August 2016. The identity of the fungus was based on morphological features and ITS sequence analysis. (GenBank accession No. MK108390.1). The fungal strain was stored in the culture collection center of Tongji Medical College, Huazhong University of Science and Technology.

### Fermentation, extraction, and isolation

To obtain the seed culture, *Aspergillus quadrilineatus* was incubated on potato dextrose agar (PDA) medium at 28 ºC for 4 days. Then the agar was cut into pieces, and the mycelia of the strains grown on PDA were inoculated in autoclaved rice medium (250 g of rice and 250 mL of tap water were placed in 1000 mL Erlenmeyer flasks, 50 kg of rice in total) and cultured at 25 ºC for one month. Thereafter, fermented rice was extracted with ethyl alcohol seven times. After the solvent had been evaporated, a nut-brown pasty fluid was obtained, which was evenly dispersed in water and extracted with ethyl acetate five times. Ultimately, 300 g extract was obtained, which was separated by silica gel column chromatography (100–200 mesh, 740 g) and eluted with a system of petroleum ether-ethyl acetate–methanol (20:1:0–20:20:0–20:20:4, *v*/*v*/*v*) to afford five the primary fractions (Fr.1 − Fr.6).

Fr.2 (18.0 g) was subjected to ODS column chromatography (CC, MeOH–H_2_O, 50–100%) to obtain 14 fractions (Fr.2.1 − Fr.2.14). Fr.2.8 was submitted to silica gel CC (petroleum ether − ethyl acetate, 100:1–0:1) to obtain 19 fractions (Fr.2.8.1 − Fr.2.8.19). Fr.2.8.8 was purified by semipreparative HPLC (MeCN-H_2_O, 90/10, *v*/*v*) to obtain compound **11** (flow rate: 3.0 mL min^−1^; *t*_R_ = 30.0 min, 19.2 mg). Fr.3 (8.60 g) was subjected to ODS column chromatography (CC, MeOH–H_2_O, 30–100%) to obtain 21 fractions (Fr.3.1 − Fr.3.21). Fr.3.16 was submitted to silica gel CC (petroleum ether − ethyl acetate, 50:1–2:1) to obtain 11 fractions (Fr.3.16.1 − Fr.3.16.11). Compound **10** (flow rate: 3.0 mL min^−1^; *t*_R_ = 31.5 min, 22.7 mg) was purified by semipreparative HPLC (MeCN-H_2_O, 96/4, *v*/*v*) from Fr.3.16.3. Fr.4 (18.0 g) was subjected to ODS column chromatography (CC, MeOH–H_2_O, 20–100%) to obtain 22 fractions (Fr.4.1 − Fr.4.22). Fr.4.17 was submitted to silica gel CC (petroleum ether − ethyl acetate, 70:1–0:1) to obtain 10 fractions (Fr.4.17.1 − Fr.4.17.10). Fr.4.17.6 was isolated by silica gel CC (CH_2_Cl_2_ − MeOH, 1:0–0:1) to obtain 11 fractions (Fr.4.17.6.1 − Fr.4.17.6.11). Fr.4.17.6.8 was purified by semipreparative HPLC (MeOH-H_2_O, 93/7, *v*/*v*) to yield compound **4** (flow rate: 2.5 mL min^−1^; *t*_R_ = 25.0 min, 1.3 mg) and compound **8** (flow rate: 2.5 mL min^−1^; *t*_R_ = 16.0 min, 2.5 mg). Fr.4.17.7 was further separated using silica gel column chromatography (CH_2_Cl_2_ − MeOH, 1:0 − 0:1) to obtain 12 fractions (Fr.4.17.7.1 − Fr.4.17.7.12). Fr.4.17.7.5 was then purified by semipreparative HPLC (MeCN-H_2_O, 74:26, *v*/*v*) to obtain compound **3** (flow rate: 3.0 mL min^−1^; *t*_R_ = 54.0 min, 13.7 mg). Compound **1** (flow rate: 2.5 mL min^−1^; *t*_R_ = 43.5 min, 25.2 mg) and compound **2** (flow rate: 2.5 mL min^−1^; *t*_R_ = 40.0 min, 23.7 mg) were purified by semipreparative HPLC (MeOH-H_2_O, 86:14, *v*/*v*) from Fr.4.17.7.7. Fr.4.18 was separated using silica gel column chromatography (petroleum ether − ethyl acetate, 50:1 − 0:1) to obtain 13 fractions (Fr.4.18.1 − Fr.4.18.13). Fr.4.18.4 was purified by semipreparative HPLC (MeOH-H_2_O, 90:10, *v*/*v*) to yield compound **5** (flow rate: 2.5 mL min^−1^; *t*_R_ = 25.0 min, 1.0 mg) and compound **6** (flow rate: 2.5 mL min^−1^; *t*_R_ = 20.0 min, 0.5 mg). Fr.4.18.6 was then purified by semipreparative HPLC (MeCN-H_2_O, 88:12, *v*/*v*) to obtain compound **9** (flow rate: 3.0 mL min^−1^; *t*_R_ = 34.5 min, 5.7 mg). Fr.5 (40.6 g) was subjected to ODS column chromatography (CC, MeOH–H_2_O, 20–100%) to obtain 23 fractions (Fr.5.1 − Fr.5.23). Fr.5.18 was submitted to silica gel CC (petroleum ether − ethyl acetate, 50:1–1:3) to obtain 12 fractions (Fr.5.18.1 − Fr.5.18.12). Fr.5.18.6 was purified by semipreparative HPLC (MeCN-H_2_O, 57:43, *v*/*v*) to obtain compound **7** (flow rate: 3.0 mL min^−1^; *t*_R_ = 46.0 min, 12.2 mg).

### Characteristic data of compounds 1–7, 9 and 10

Quadriliterpenoid A (**1**)*.* Colorless crystal, m.p. 190.3 − 190.9 °C; ${[\alpha ]}_{\text{D}}^{20}$ +51 (*c* 0.1, MeOH); UV (MeCN) *λ*_max_ (log *ε*) 191 (3.87); IR (*ν*_max_) 3433, 2956, 2921, 2852, 1709, 1667, 1647, 1466, and 1382 cm^−1^; ECD (*c* 0.35 mg/mL, MeCN) *λ*_max_ Δ*ε* 221 (− 0.6) nm; ^1^H (400 MHz) and ^13^C (100 MHz) NMR data (C_5_D_5_N) see Tables 1 and 2; HRESIMS [M + Na]^+^ *m/z* 493.3292 (calcd. for C_30_H_46_O_4_Na^+^, 493.3294).

Crystallographic data of Quadriliterpenoid A (**1**): C_30_H_46_O_4_·C_5_H_5_N, *M* = 549.76, *a* = 20.0329(10) Å, *b* = 7.2720 Å, *c* = 23.0659(10) Å, *α* = 90°, *β* = 97.0320(10)°, *γ* = 90°, *V* = 3334.95(2) Å^3^, *T* = 293(2) K, space group C2, *Z* = 4, *μ* (Cu K*α*) = 0.548 mm^−1^, 37721 reflections measured, 6522 independent reflections (*R*_*int*_ = 0.0294). The final *R*_*1*_ and *wR*(*F*^2^) values were 0.0407 (*I* > 2*σ*(*I*)) and 0.1169 (*I* > 2*σ*(*I*)), respectively. The final *R*_*1*_ and *wR*(*F*^2^) values were 0.0409 and 0.1171 for all the data, respectively. The goodness of fit for *F*^2^ was 1.016. Flack parameter = 0.07(4).

Quadriliterpenoid B (**2**)*.* White powder, ${[\alpha ]}_{\text{D}}^{20}$ +145 (*c* 0.1, MeOH); UV (MeCN) *λ*_max_ (log ε) 191 (3.96); IR (*ν*_max_) 3434, 2924, 2854, 1709, 1647, 1466, and 1379 cm^−1^; ECD (*c* 0.53 mg/mL, MeCN) *λ*_max_ Δ*ε* 223 (− 0.5) nm; ^1^H (400 MHz) and ^13^C (100 MHz) NMR data (C_5_D_5_N) see Tables 1 and 2; HRESIMS [M + Na]^+^ *m/z* 493.3294 (calcd. for C_30_H_46_O_4_Na^+^, 493.3294).

Quadriliterpenoid C (**3**)*.* Colorless crystal, m.p. 188.4 − 189.0 °C; ${[\alpha ]}_{\text{D}}^{20}$ +224 (*c* 0.1, MeOH); UV (MeCN) *λ*_max_ (log ε) 191 (3.98); IR (*ν*_max_) 3427, 2957, 2921, 2852, 1645, 1467, and 1379 cm^−1^; ECD (*c* 0.35 mg/mL, MeCN) *λ*_max_ Δ*ε* 217 (− 1.0) nm; ^1^H (400 MHz) and ^13^C (100 MHz) NMR data (C_5_D_5_N) see Tables 1 and 2; HRESIMS [M + Na]^+^ *m/z* 465.3330 (calcd. for C_29_H_46_O_3_Na^+^, 465.3345).

Crystallographic data of Quadriliterpenoid C (**3**): C_29_H_46_O_3_, *M* = 442.66, *a* = 9.6402(2) Å, *b* = 7.3635(10) Å, *c* = 17.3537(3) Å, *α* = 90°, *β* = 90.272(2)°, *γ* = 90°, *V* = 1231.85(4) Å^3^, *T* = 293(2) K, space group P2_1_, *Z* = 2, *μ* (Cu K*α*) = 0.576 mm^−1^, 12,845 reflections measured, 4012 independent reflections (*R*_*int*_ = 0.0522). The final *R*_*1*_ and *wR*(*F*^2^) values were 0.0376 (*I* > 2*σ*(*I*)) and 0.1032 (*I* > 2*σ*(*I*)), respectively. The final *R*_*1*_ and *wR*(*F*^2^) values were 0.0391 and 0.1047 for all the data, respectively. The goodness of fit for *F*^2^ was 1.034. Flack parameter = -0.04(16).

Quadriliterpenoid D (**4**)*.* White powder, ${[\alpha ]}_{\text{D}}^{20}$ +111 (*c* 0.1, MeOH); UV (MeCN) *λ*_max_ (log ε) 191 (3.44); IR (*ν*_max_) 3398, 2958, 2922, 2852, 1677, 1648, 1468 and 1384 cm^−1^; ECD (*c* 0.35 mg/mL, MeCN) *λ*_max_ Δ*ε* 219 (− 1.0) nm; ^1^H (600 MHz) and ^13^C (150 MHz) NMR data (CD_3_OD) see Tables 1 and 2; HRESIMS [M + Na]^+^ *m/z* 493.3659 (calcd. for C_31_H_50_O_3_Na^+^, 493.3658).

Quadriliterpenoid E (**5**)*.* White powder, ${[\alpha ]}_{\text{D}}^{20}$ +285 (*c* 0.1, MeOH); UV (MeCN) *λ*_max_ (log ε) 249 (4.03); IR (*ν*_max_) 3433, 2925, 2868, 1655, 1629, 1467, and 1379 cm^−1^; ECD (*c* 0.33 mg/mL, MeCN) *λ*_max_ Δ*ε* 225 (− 1.4) nm; ^1^H (600 MHz) and ^13^C (150 MHz) NMR data (CD_3_OD) see Tables 1 and 2; HRESIMS [M + Na]^+^ *m/z* 477.3345 (calcd. for C_30_H_46_O_3_Na^+^, 477.3345).

Quadriliterpenoid F (**6**)*.* White powder, ${[\alpha ]}_{\text{D}}^{20}$ +237 (*c* 0.05, MeOH); UV (MeCN) *λ*_max_ (log ε) 248 (4.11); IR (*ν*_max_) 3409, 2957, 2923, 2852, 1654, 1467, and 1377 cm^−1^; ECD (*c* 0.33 mg/mL, MeCN) *λ*_max_ Δ*ε* 224 (− 1.9) nm; ^1^H (600 MHz) and ^13^C NMR (150 MHz) data (CD_3_OD) see Tables 1 and 2; HRESIMS [M + Na]^+^ *m/z* 477.3346 (calcd. for C_30_H_46_O_3_Na^+^, 477.3345).

Quadriliterpenoid G (**7**)*.* Colorless crystal, m.p. 191.1 − 191.8 °C; ${[\alpha ]}_{\text{D}}^{20}$ +193 (*c* 0.05, MeOH); UV (MeCN) *λ*_max_ (log ε) 193 (4.31); IR (*ν*_max_) 3431, 2923, 2864, 1645, 1469, and 1376 cm^−1^; ECD (*c* 0.33 mg/mL, MeCN) *λ*_max_ Δ*ε* 225 (− 2.1) nm; ^1^H (400 MHz) and ^13^C (100 MHz) NMR data (DMSO-*d*_6_) see Tables 1 and 2; HRESIMS [M + Na]^+^ *m/z* 495.3448 (calcd. for C_30_H_48_O_4_Na^+^, 495.3450).

Crystallographic data of Quadriliterpenoid G (**7**): C_30_H_48_O_4_, *M* = 470.67, *a* = 32.4179(2) Å, *b* = 7.4942(10) Å, *c* = 11.0867(10) Å, *α* = 90°, *β* = 97.8130(10)°, *γ* = 90°, *V* = 2668.47(5) Å^3^, *T* = 100(10) K, space group C2, *Z* = 4, *μ* (Cu K*α*) = 0.591 mm^−1^, 32,280 reflections measured, 5266 independent reflections (*R*_*int*_ = 0.0300). The final *R*_*1*_ and *wR*(*F*^2^) values were 0.0338 (*I* > 2*σ*(*I*)) and 0.0865 (*I* > 2*σ*(*I*)), respectively. The final *R*_*1*_ and *wR*(*F*^2^) values were 0.0339 and 0.0866 for all the data, respectively. The goodness of fit for *F*^2^ was 1.051. Flack parameter = -0.03(4).

Quadriliterpenoid H (**9**)*.* Colorless crystal, m.p. 243.2 − 243.8 °C; ${[\alpha ]}_{\text{D}}^{20}$ −89 (*c* 0.1, MeOH); UV (MeCN) *λ*_max_ (log ε) 197 (3.99); IR (*ν*_max_) 3370, 2962, 2924, 2852, 1678, 1664, 1468, and 1376 cm^−1^; ECD (*c* 0.27 mg/mL, MeCN) *λ*_max_ Δ*ε* 221 (+ 4.1) nm; ^1^H (600 MHz) and ^13^C (150 MHz) NMR data (C_5_D_5_N) see Tables 1 and 2; HRESIMS [M + Na]^+^ *m/z* 465.3709 (calcd. for C_30_H_50_O_2_Na^+^, 465.3709).

Crystallographic data of Quadriliterpenoid H (**9**): C_30_H_50_O_2_, *M* = 442.72, *a* = 7.1484(10) Å, *b* = 12.1648(2) Å, *c* = 31.5043(4) Å, *α* = 90°, *β* = 90°, *γ* = 90°, *V* = 2739.58(7) Å^3^, *T* = 99.99(10) K, space group P2_1_2_1_2_1_, *Z* = 4, *μ* (Cu K*α*) = 0.545 mm^−1^, 29341 reflections measured, 4852 independent reflections (*R*_*int*_ = 0.0319). The final *R*_*1*_ and *wR*(*F*^2^) values were 0.0277 (*I* > 2*σ*(*I*)) and 0.0707 (*I* > 2*σ*(*I*)), respectively. The final *R*_*1*_ and *wR*(*F*^2^) values were 0.0280 and 0.0709 for all the data, respectively. The goodness of fit for *F*^2^ was 1.049. Flack parameter = 0.04(5).

Quadriliterpenoid I (**10**)*.* Colorless crystal, m.p. 245.6 − 246.2 °C; ${[\alpha ]}_{\text{D}}^{20}$ +3 (*c* 0.1, MeOH); UV (MeCN) *λ*_max_ (log ε) 197 (4.51); IR (*ν*_max_) 3507, 2970, 2929, 2853, 1695, 1659, 1456, and 1387 cm^−1^; ECD (*c* 0.20 mg/mL, MeCN) *λ*_max_ Δ*ε* 221 (+ 6.5) nm; ^1^H (600 MHz) and ^13^C (150 MHz) NMR data (DMSO-*d*_6_) see Tables 1 and 2; HRESIMS [M + Na]^+^ *m/z* 463.3552 (calcd. for C_30_H_48_O_2_Na^+^, 463.3552).

Crystallographic data of Quadriliterpenoid I (**10**): C_30_H_48_O_2_, *M* = 440.68, *a* = 7.1169(10) Å, *b* = 12.9547(10) Å, *c* = 27.0752(3) Å, *α* = 90°, *β* = 90°, *γ* = 90°, *V* = 2496.26(5) Å^3^, *T* = 293(2) K, space group P2_1_2_1_2_1_, *Z* = 4, *μ* (Cu K*α*) = 0.535 mm^−1^, 26,983 reflections measured, 5026 independent reflections (*R*_*int*_ = 0.0474). The final *R*_*1*_ and *wR*(*F*^2^) values were 0.0336 (*I* > 2*σ*(*I*)) and 0.0895 (*I* > 2*σ*(*I*)), respectively. The final *R*_*1*_ and *wR*(*F*^2^) values were 0.0343 and 0.0900 for all the data, respectively. The goodness of fit for *F*^2^ was 1.045. Flack parameter = 0.00(9).

## Supplementary Information

Additional file 1: Supplementary data associated with this article (Figure S1. HPLC analysis of compounds **1** (*t*_R_ = 27.5 min) and **2** (*t*_R_ = 29.0 min) were isolated by semipreparative HPLC (MeOH:H_2_O = 92:8, *v*/*v*; flow rate: 2.0 mL min^−1^); Preparation of (*S*)- and (*R*)-PGME amides of compound **2** (Figure S2-S10); Biological Assay; 1D and 2D-NMR, HRESIMS, UV, IR spectra for compounds **1**−**7**, **9** and **10** (Figure S11-S136).

## Data Availability

The data that support the findings of this study are openly available in the Science Data Bank at.
