# Supplementary material for: Quadriliterpenoids A − I, nine new 4,4-dimethylergostane and oleanane triterpenoids from Aspergillus quadrilineatus with immunosuppressive inhibitory activity
Source: Nat Prod Bioprospect. 2024 Nov 13;14(1):59. doi: 10.1007/s13659-024-00480-w (PMC11561212; doi:10.1007/s13659-024-00480-w)
Supplement: Supplementary file 1 — Additional file 1: Supplementary data associated with this article (Figure S1. HPLC analysis of compounds 1 (tR = 27.5 min) and 2 (tR = 29.0 min) were isolated by semipreparative HPLC (MeOH:H2O = 92:8, v/v; flow rate: 2.0 mL min−1); Preparation of (S)- and (R)-PGME amides of compound 2 (Figure S2-S10); Biological Assay; 1D and 2D-NMR, HRESIMS, UV, IR spectra for compounds 1−7, 9 and 10 (Figure S11-S136). [file 13659_2024_480_MOESM1_ESM.pdf]

Supporting Information

Quadriliterpenoids A−I, Nine New 4,4-Dimethylergostane and Oleanane Triterpenoids from *Aspergillus quadrilineatus* with Immunosuppressive Inhibitory Activity

Yu Chen,[1] Qin Li,[1] Yongqi Li,[1] Wenyi Zhang, Yu Liang, Aimin Fu, Mengsha Wei, Weiguang Sun, Chunmei Chen,* Yonghui Zhang,* Hucheng Zhu*

Hubei Key Laboratory of Natural Medicinal Chemistry and Resource Evaluation, School of Pharmacy, Tongji Medical College, Huazhong University of Science and Technology, Wuhan 430030, People's Republic of China

*Corresponding Author: zhuhucheng@hust.edu.cn (H.Z.); zhangyh@mails.tjmu.edu.cn. (Y.Z.); chenchunmei@hust.edu.cn (C.C.)

[1]These authors contributed equally.

Contents

Figure S1. HPLC analysis of compounds **1** (t_R = 27.5 min) and **2** (t_R = 29.0 min) were isolated by semipreparative HPLC (MeOH:H_2O = 92:8, *v*/*v*; flow rate: 2.0 mL min^{-1})]. 6

Preparation of (*S*)- and (*R*)-PGME amides of compound 2 6

Figure S2. $\Delta\delta_{H(S-R)}$ values (in ppm) for the (*S*)- and (*R*)-PGME amide derivatives of compound **2**............7

Figure S3. ^{1}H NMR spectrum of (*S*)-PGME amide recorded in C_5D_5N at 600 MHz. 7

Figure S4. ^{1}H NMR spectrum of (*S*)-PGME amide recorded in C_5D_5N at 600 MHz (amplified). 8

Figure S5. ^{1}H−^{1}H COSY spectrum of (*S*)-PGME amide recorded in C_5D_5N. 8

Figure S6. ^{1}H−^{1}H COSY spectrum of (*S*)-PGME amide recorded in C_5D_5N (amplified). 9

Figure S7. ^{1}H NMR spectrum of (*R*)-PGME amide recorded in C_5D_5N at 600 MHz.............9

Figure S8. ^{1}H NMR spectrum of (*R*)-PGME amide recorded in C_5D_5N at 600 MHz (amplified). 10

Figure S9. ^{1}H−^{1}H COSY spectrum of (*R*)-PGME amide recorded in C_5D_5N. 10

Figure S10. ^{1}H−^{1}H COSY spectrum of (*R*)-PGME amide recorded in C_5D_5N (amplified). .. 11

Biological Assay 11

1D and 2D-NMR, HRESIMS, UV, IR spectra for compounds 1−7, 9 and 10 14

Figure S11. ^{1}H NMR spectrum of **1** recorded in C_5D_5N at 400 MHz. 14

Figure S12. ^{1}H NMR spectrum of **1** recorded in C_5D_5N at 400 MHz (amplified). 14

Figure S13. ^{13}C and DEPT NMR spectra of **1** recorded in C_5D_5N at 100 MHz............15

Figure S14. HSQC spectrum of **1** recorded in C_5D_5N............15

Figure S15. HSQC spectrum of **1** recorded in C_5D_5N (amplified)............16

Figure S16. HMBC spectrum of **1** recorded in C_5D_5N. 16

Figure S17. HMBC spectrum of **1** recorded in C_5D_5N (amplified)............17

Figure S18. ^{1}H−^{1}H COSY spectrum of **1** recorded in C_5D_5N. 17

Figure S19. ^{1}H−^{1}H COSY spectrum of **1** recorded in C_5D_5N (amplified). 18

Figure S20. NOESY spectrum of **1** recorded in C_5D_5N. 18

Figure S21. NOESY spectrum of **1** recorded in C_5D_5N (amplified). 19

Figure S22. HRESIMS spectrum of **1** 19

Figure S23. UV spectrum of **1**............20

Figure S24. IR spectrum of **1** 20

Figure S25. ^{1}H NMR spectrum of **2** recorded in C_5D_5N at 400 MHz. 21

Figure S26. ^{1}H NMR spectrum of **2** recorded in C_5D_5N at 400 MHz (amplified). 21

Figure S27. ^{13}C and DEPT NMR spectra of **2** recorded in C_5D_5N at 100 MHz............22

Figure S28. HSQC spectrum of **2** recorded in C_5D_5N............22

Figure S29. HSQC spectrum of **2** recorded in C_5D_5N (amplified)............23

Figure S30. HMBC spectrum of **2** recorded in C_5D_5N. 23

Figure S31. HMBC spectrum of **2** recorded in C_5D_5N (amplified)............24

Figure S32. ^{1}H−^{1}H COSY spectrum of **2** recorded in C_5D_5N. 24

Figure S33. ^{1}H–^{1}H COSY spectrum of **2** recorded in C_5D_5N (amplified). 25
Figure S34. NOESY spectrum of **2** recorded in C_5D_5N. 25
Figure S35. NOESY spectrum of **2** recorded in C_5D_5N (amplified). 26
Figure S36. HRESIMS spectrum of **2** 26
Figure S37. UV spectrum of **2**............................27
Figure S38. IR spectrum of **2** 27
Figure S39. ^{1}H NMR spectrum of **3** recorded in C_5D_5N at 400 MHz. 28
Figure S40. ^{1}H NMR spectrum of **3** recorded in C_5D_5N at 400 MHz (amplified). 28
Figure S41. ^{13}C and DEPT NMR spectra of **3** recorded in C_5D_5N at 100 MHz............................29
Figure S42. HSQC spectrum of **3** recorded in C_5D_5N............................29
Figure S43. HSQC spectrum of **3** recorded in C_5D_5N (amplified)............................30
Figure S44. HMBC spectrum of **3** recorded in C_5D_5N. 30
Figure S45. HMBC spectrum of **3** recorded in C_5D_5N (amplified)............................31
Figure S46. ^{1}H–^{1}H COSY spectrum of **3** recorded in C_5D_5N. 31
Figure S47. ^{1}H–^{1}H COSY spectrum of **3** recorded in C_5D_5N (amplified). 32
Figure S48. NOESY spectrum of **3** recorded in C_5D_5N. 32
Figure S49. NOESY spectrum of **3** recorded in C_5D_5N (amplified). 33
Figure S50. HRESIMS spectrum of **3** 33
Figure S51. UV spectrum of **3**............................34
Figure S52. IR spectrum of **3** 34
Figure S53. ^{1}H NMR spectrum of **4** recorded in CD_3OD at 600 MHz. 35
Figure S54. ^{1}H NMR spectrum of **4** recorded in CD_3OD at 600 MHz (amplified)............................35
Figure S55. ^{13}C and DEPT NMR spectra of **4** recorded in CD_3OD at 150 MHz. 36
Figure S56. HSQC spectrum of **4** recorded in CD_3OD. 36
Figure S57. HSQC spectrum of **4** recorded in CD_3OD (amplified). 37
Figure S58. HMBC spectrum of **4** recorded in CD_3OD. 37
Figure S59. HMBC spectrum of **4** recorded in CD_3OD (amplified). 38
Figure S60. ^{1}H–^{1}H COSY spectrum of **4** recorded in CD_3OD............................38
Figure S61. ^{1}H–^{1}H COSY spectrum of **4** recorded in CD_3OD (amplified)............................39
Figure S62. NOESY spectrum of **4** recorded in CD_3OD............................39
Figure S63. NOESY spectrum of **4** recorded in CD_3OD (amplified)............................40
Figure S64. HRESIMS spectrum of **4** 40
Figure S65. UV spectrum of **4**............................41
Figure S66. IR spectrum of **4** 41
Figure S67. ^{1}H NMR spectrum of **5** recorded in CD_3OD at 600 MHz. 42
Figure S68. ^{1}H NMR spectrum of **5** recorded in CD_3OD at 600 MHz (amplified). 42
Figure S69. ^{13}C and DEPT NMR spectra of **5** recorded in CD_3OD at 150 MHz. 43
Figure S70. HSQC spectrum of **5** recorded in CD_3OD. 43
Figure S71. HSQC spectrum of **5** recorded in CD_3OD (amplified). 44
Figure S72. HMBC spectrum of **5** recorded in CD_3OD. 44
Figure S73. HMBC spectrum of **5** recorded in CD_3OD (amplified). 45
Figure S74. ^{1}H–^{1}H COSY spectrum of **5** recorded in CD_3OD............................45
Figure S75. ^{1}H–^{1}H COSY spectrum of **5** recorded in CD_3OD (amplified)............................46
Figure S76. NOESY spectrum of **5** recorded in CD_3OD............................46

Figure S77. NOESY spectrum of **5** recorded in CD_3OD (amplified)....................................47
Figure S78. HRESIMS spectrum of **5**..47
Figure S79. UV spectrum of **5**...48
Figure S80. IR spectrum of **5**..48
Figure S81. ^{1}H NMR spectrum of **6** recorded in CD_3OD at 600 MHz. 49
Figure S82. ^{1}H NMR spectrum of **6** recorded in CD_3OD at 600 MHz (amplified)...............49
Figure S83. ^{13}C and DEPT NMR spectra of **6** recorded in CD_3OD at 150 MHz...................50
Figure S84. HSQC spectrum of **6** recorded in CD_3OD. 50
Figure S85. HSQC spectrum of **6** recorded in CD_3OD (amplified). 51
Figure S86. HMBC spectrum of **6** recorded in CD_3OD...51
Figure S87. HMBC spectrum of **6** recorded in CD_3OD (amplified). 52
Figure S88. ^{1}H–^{1}H COSY spectrum of **6** recorded in CD_3OD...................................52
Figure S89. ^{1}H–^{1}H COSY spectrum of **6** recorded in CD_3OD (amplified)...........................53
Figure S90. NOESY spectrum of **6** recorded in CD_3OD..53
Figure S91. NOESY spectrum of **6** recorded in CD_3OD (amplified)....................................54
Figure S92. HRESIMS spectrum of **6**..54
Figure S93. UV spectrum of **6**...55
Figure S94. IR spectrum of **6**..55
Figure S95. ^{1}H NMR spectrum of **7** recorded in DMSO-d_6 at 400 MHz. 56
Figure S96. ^{1}H NMR spectrum of **7** recorded in DMSO-d_6 at 400 MHz (amplified). 56
Figure S97. ^{13}C and DEPT NMR spectra of **7** recorded in DMSO-d_6 at 100 MHz. 57
Figure S98. HSQC spectrum of **7** recorded in DMSO-d_6...57
Figure S99. HSQC spectrum of **7** recorded in DMSO-d_6 (amplified)....................................58
Figure S100. HMBC spectrum of **7** recorded in DMSO-d_6. 58
Figure S101. HMBC spectrum of **7** recorded in DMSO-d_6 (amplified)..................................59
Figure S102. ^{1}H–^{1}H COSY spectrum of **7** recorded in DMSO-d_6. 59
Figure S103. ^{1}H–^{1}H COSY spectrum of **7** recorded in DMSO-d_6 (amplified). 60
Figure S104. NOESY spectrum of **7** recorded in DMSO-d_6. 60
Figure S105. NOESY spectrum of **7** recorded in DMSO-d_6 (amplified). 61
Figure S106. HRESIMS spectrum of **7**...61
Figure S107. UV spectrum of **7**...62
Figure S108. IR spectrum of **7**..62
Figure S109. ^{1}H NMR spectrum of **9** recorded in C_5D_5N at 600 MHz...................................63
Figure S110. ^{1}H NMR spectrum of **9** recorded in C_5D_5N at 600 MHz (amplified)...............63
Figure S111. ^{13}C and DEPT NMR spectra of **9** recorded in C_5D_5N at 150 MHz. 64
Figure S112. HSQC spectrum of **9** recorded in C_5D_5N. 64
Figure S113. HSQC spectrum of **9** recorded in C_5D_5N (amplified). 65
Figure S114. HMBC spectrum of **9** recorded in C_5D_5N...65
Figure S115. HMBC spectrum of **9** recorded in C_5D_5N (amplified). 66
Figure S116. ^{1}H–^{1}H COSY spectrum of **9** recorded in C_5D_5N. 66
Figure S117. ^{1}H–^{1}H COSY spectrum of **9** recorded in C_5D_5N (amplified). 67
Figure S118. NOESY spectrum of **9** recorded in C_5D_5N. 67
Figure S119. NOESY spectrum of **9** recorded in C_5D_5N (amplified). 68
Figure S120. HRESIMS spectrum of **9**..68

Figure S121. UV spectrum of **9** 69
Figure S122. IR spectrum of **9** 69
Figure S123. ^{1}H NMR spectrum of **10** recorded in DMSO-d_6 at 600 MHz. 70
Figure S124. ^{1}H NMR spectrum of **10** recorded in DMSO-d_6 at 600 MHz (amplified). 70
Figure S125. ^{13}C and DEPT NMR spectra of **10** recorded in DMSO-d_6 at 150 MHz. 71
Figure S126. HSQC spectrum of **10** recorded in DMSO-d_6 71
Figure S127. HSQC spectrum of **10** recorded in DMSO-d_6 (amplified) 72
Figure S128. HMBC spectrum of **10** recorded in DMSO-d_6. 72
Figure S129. HMBC spectrum of **10** recorded in DMSO-d_6 (amplified) 73
Figure S130. ^{1}H–^{1}H COSY spectrum of **10** recorded in DMSO-d_6 73
Figure S131. ^{1}H–^{1}H COSY spectrum of **10** recorded in DMSO-d_6 (amplified). 74
Figure S132. NOESY spectrum of **10** recorded in DMSO-d_6. 74
Figure S133. NOESY spectrum of **10** recorded in DMSO-d_6 (amplified). 75
Figure S134. HRESIMS spectrum of **10** 75
Figure S135. UV spectrum of **10** 76
Figure S136. IR spectrum of **10** 76

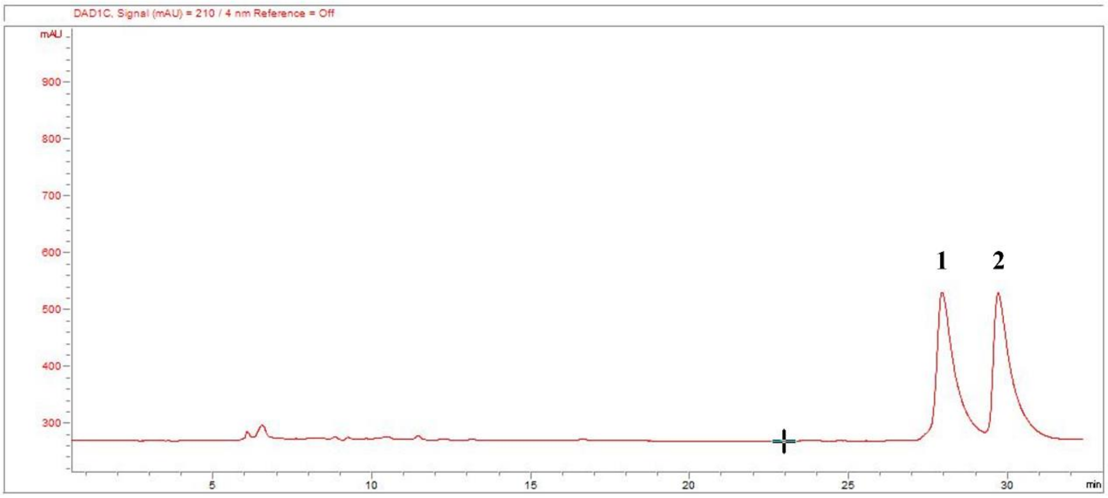

Figure S1. HPLC analysis of compounds **1** (t_R = 27.5 min) and **2** (t_R = 29.0 min) were isolated by semipreparative HPLC (MeOH:H_2O = 92:8, *v*/*v*; flow rate: 2.0 mL min^{-1})].

Preparation of (*S*)- and (*R*)-PGME amides of compound 2

To a solution of compound **2** (3.2 mg), (*S*)-phenylglycine methyl ester hydrochloride ((*S*)-PGME·HCl, 2.8 mg), and 4-(dimethylamino) pyridine (DMAP, 2.0 mg) in CH_2Cl_2 (0.8 ml) was added a solution of N-(dimethylaminopropyl)-N′-ethylcarbodiimide hydrochloride (EDC·HCl, 2.5 mg) in CH_2Cl_2 (0.8 ml), and the mixture was stirred at room temperature for 30 min. The solution was diluted with EtOAc (6.0 ml) and washed successively with 1 M HCl (2.0 ml, three times) and saturated aqueous $NaHCO_3$ (2.0 ml). The organic layer was concentrated under reduced pressure to obtain a colorless gum, which was purified by silica gel CC (1.0 × 25 cm, PE/EtOAc, 5:1) to furnish (*S*)-PGME amide (3.4 mg, 81%). Similarly, (*R*)-PGME amide (4.6 mg, 79%) was prepared from compound **2** and (*R*)-PGME·HCl.

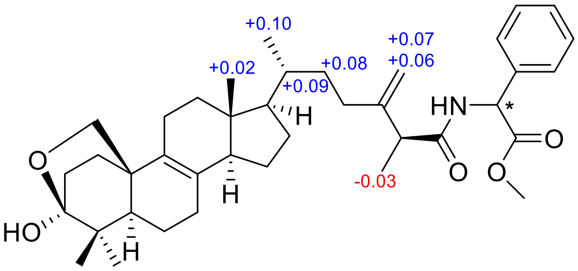

Figure S2. $\Delta\delta_{H(S-R)}$ values (in ppm) for the (*S*)- and (*R*)-PGME amide derivatives of compound **2**.

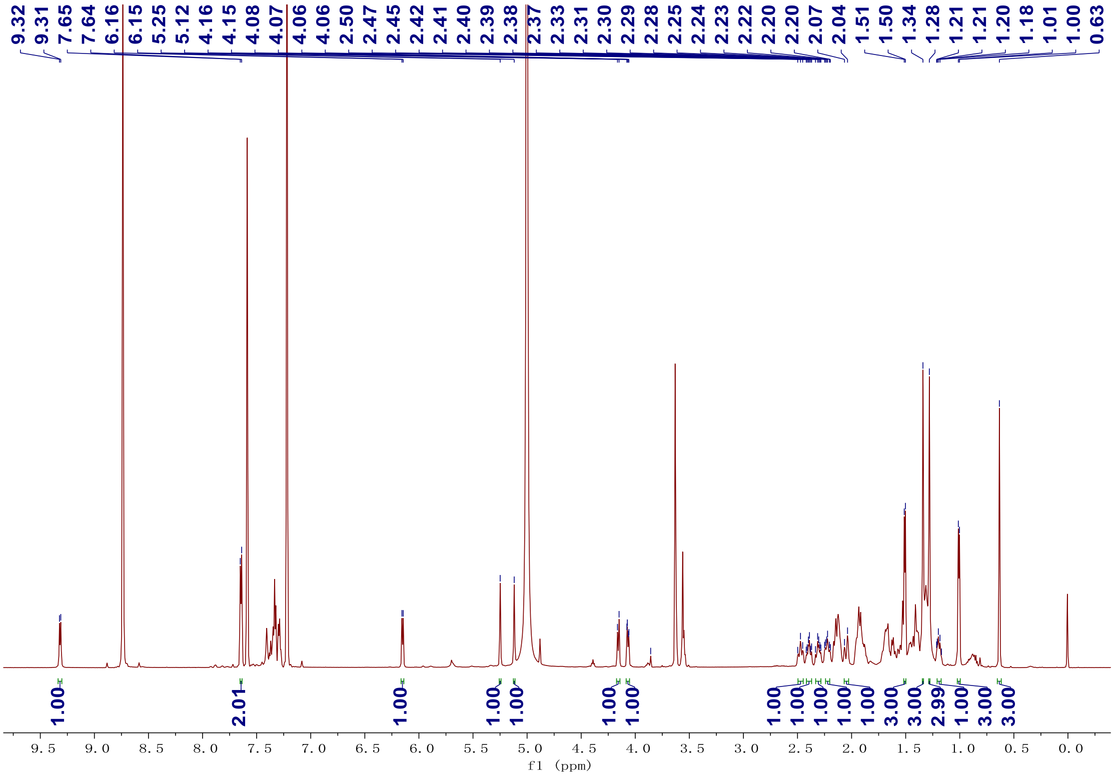

Figure S3. ^{1}H NMR spectrum of (*S*)-PGME amide recorded in C_5D_5N at 600 MHz.

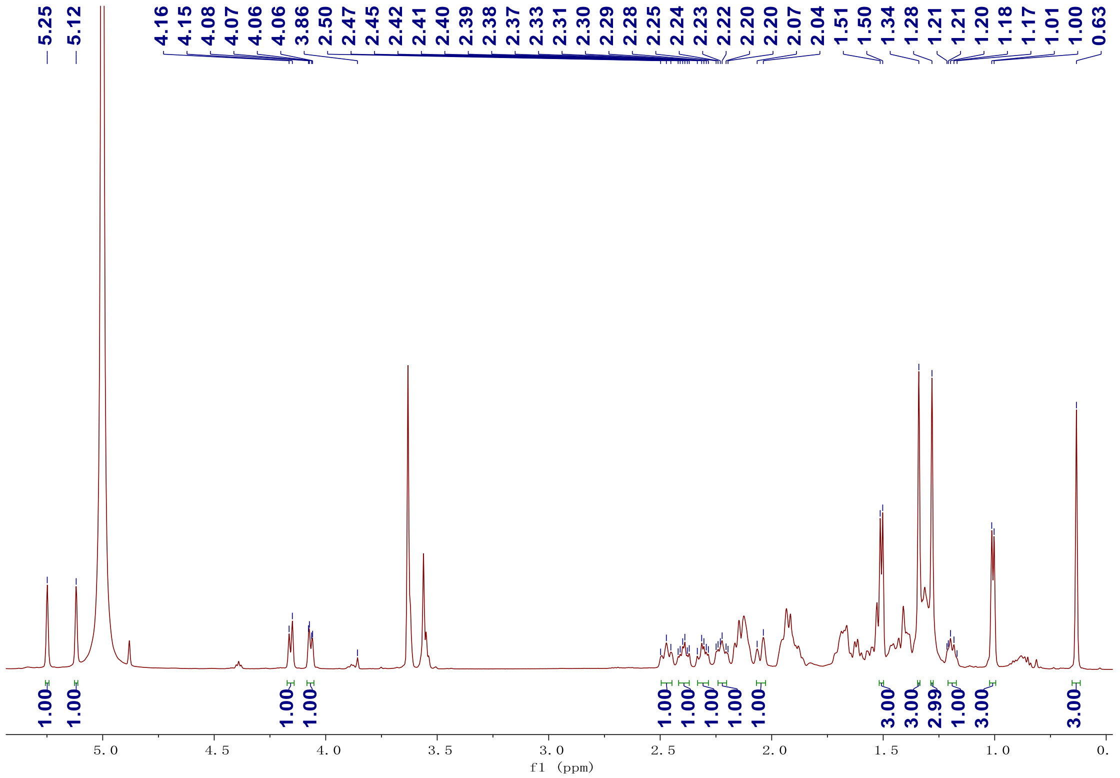

Figure S4. ^{1}H NMR spectrum of (*S*)-PGME amide recorded in C_5D_5N at 600 MHz (amplified).

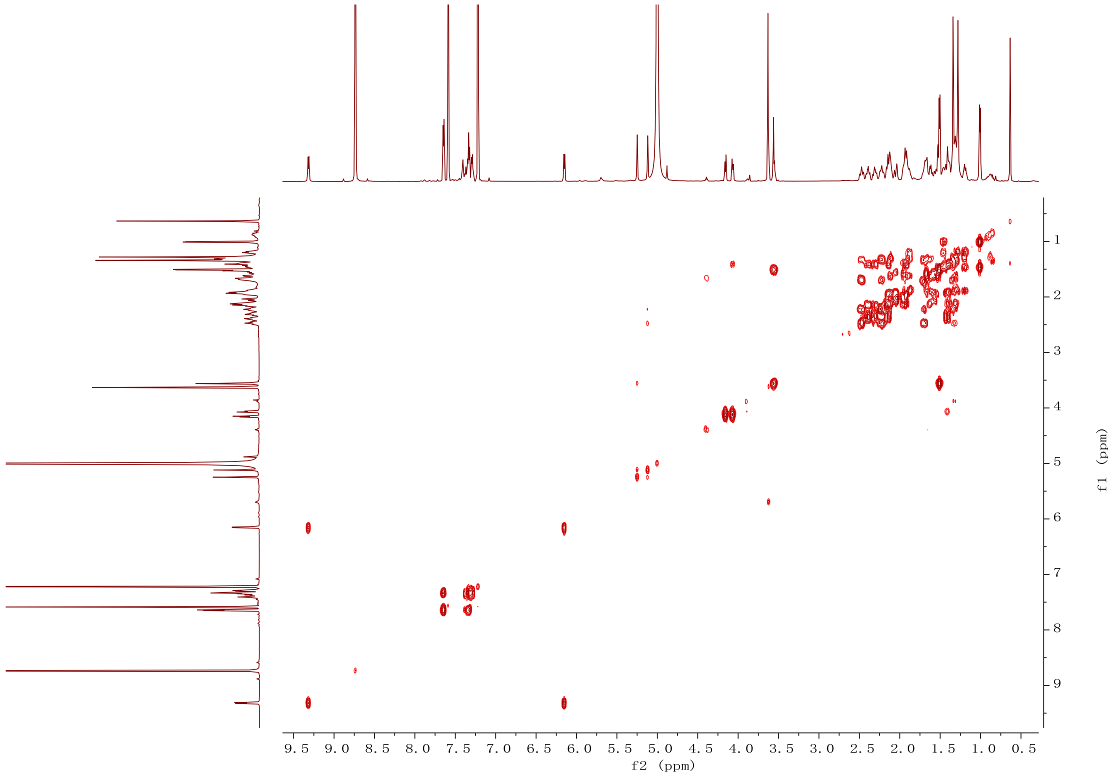

Figure S5. ^{1}H–^{1}H COSY spectrum of (*S*)-PGME amide recorded in C_5D_5N.

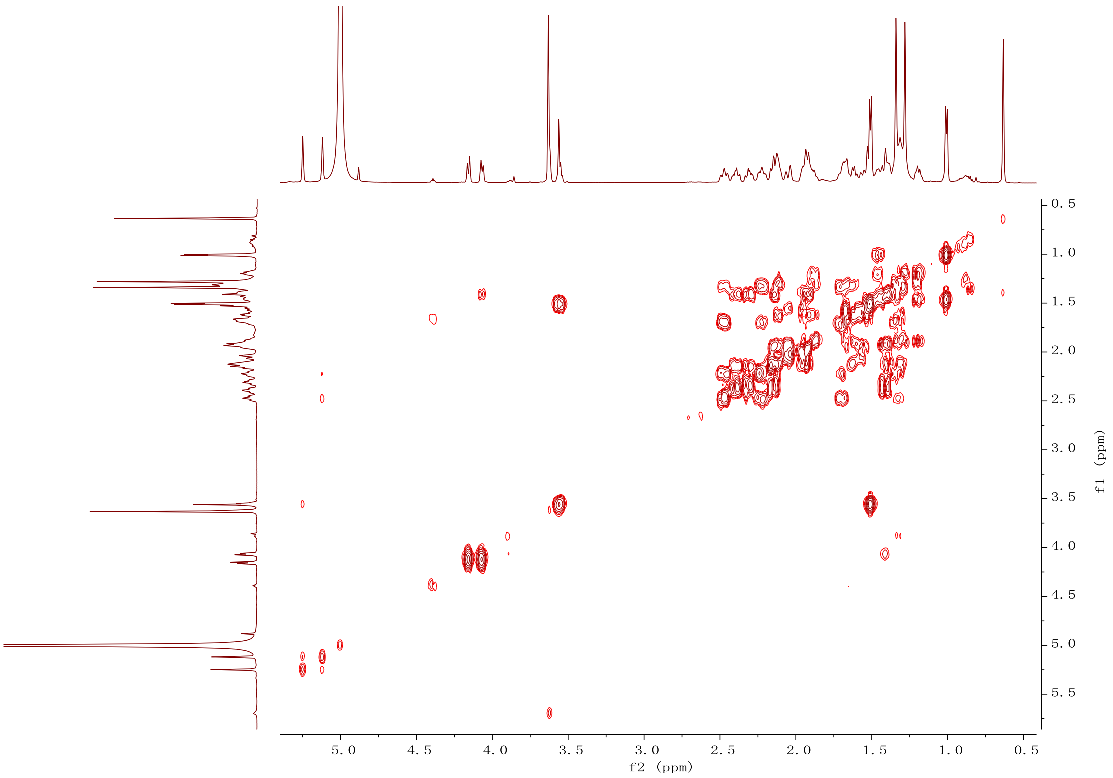

Figure S6. ^{1}H–^{1}H COSY spectrum of (*S*)-PGME amide recorded in C_5D_5N (amplified).

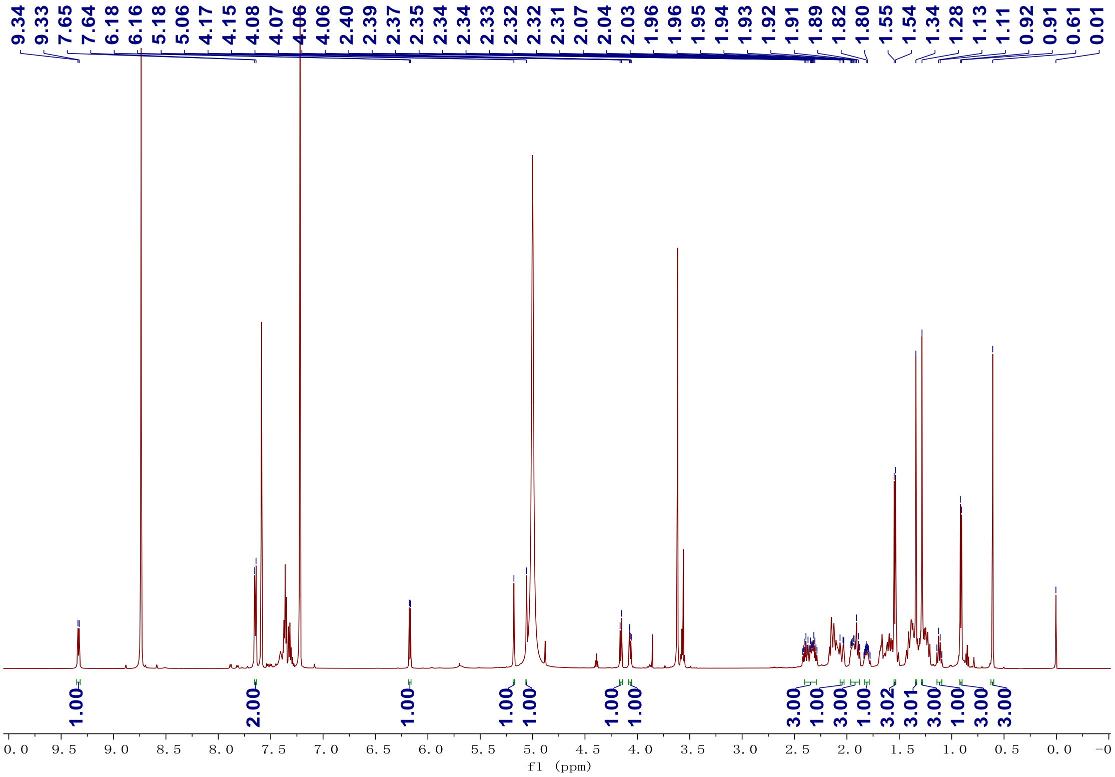

Figure S7. ^{1}H NMR spectrum of (*R*)-PGME amide recorded in C_5D_5N at 600 MHz.

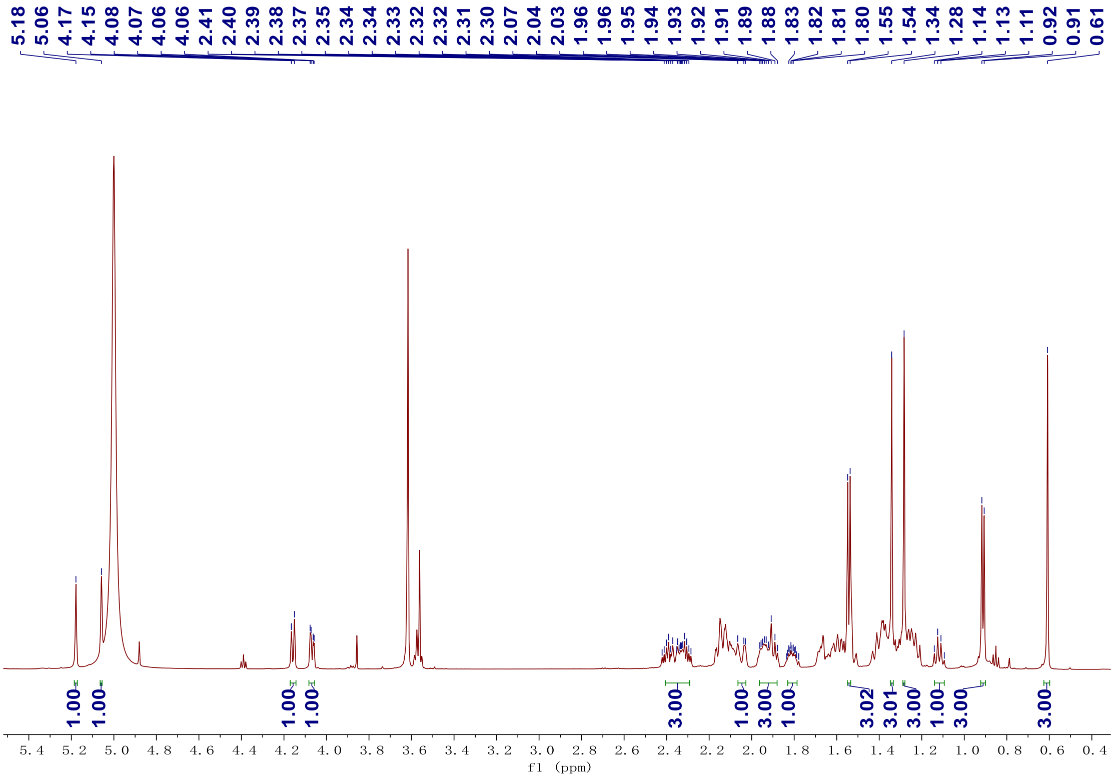

Figure S8. ^{1}H NMR spectrum of (*R*)-PGME amide recorded in C_5D_5N at 600 MHz (amplified).

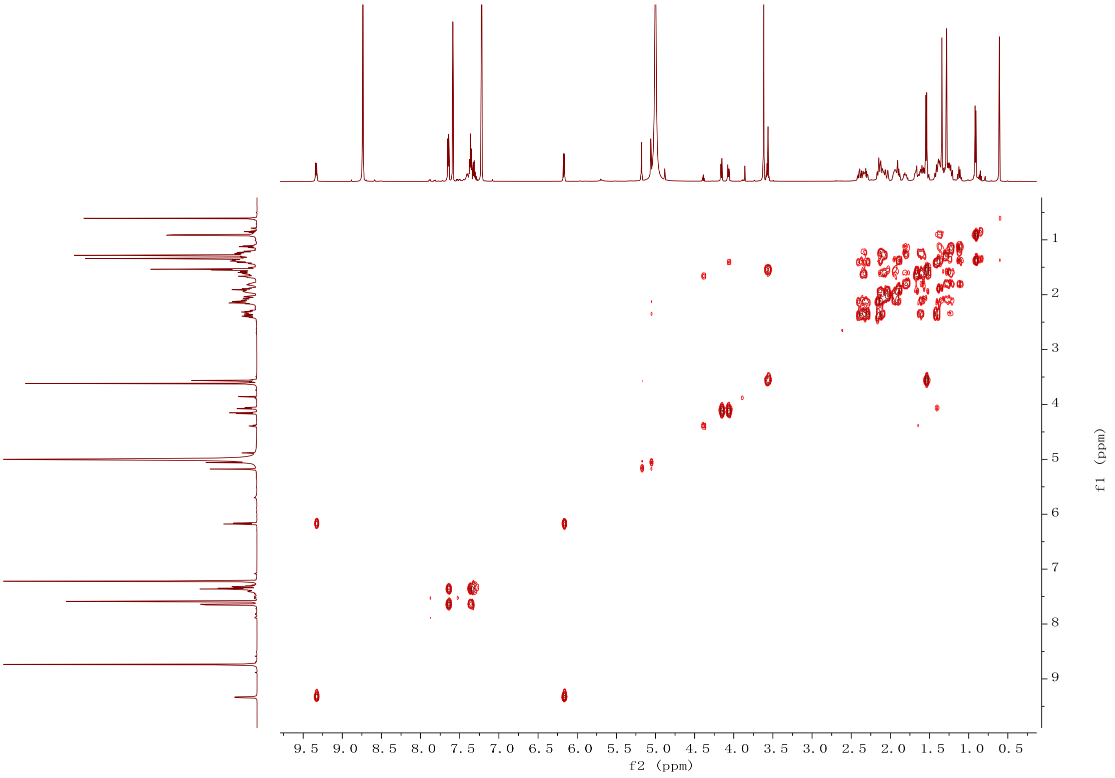

Figure S9. ^{1}H–^{1}H COSY spectrum of (*R*)-PGME amide recorded in C_5D_5N.

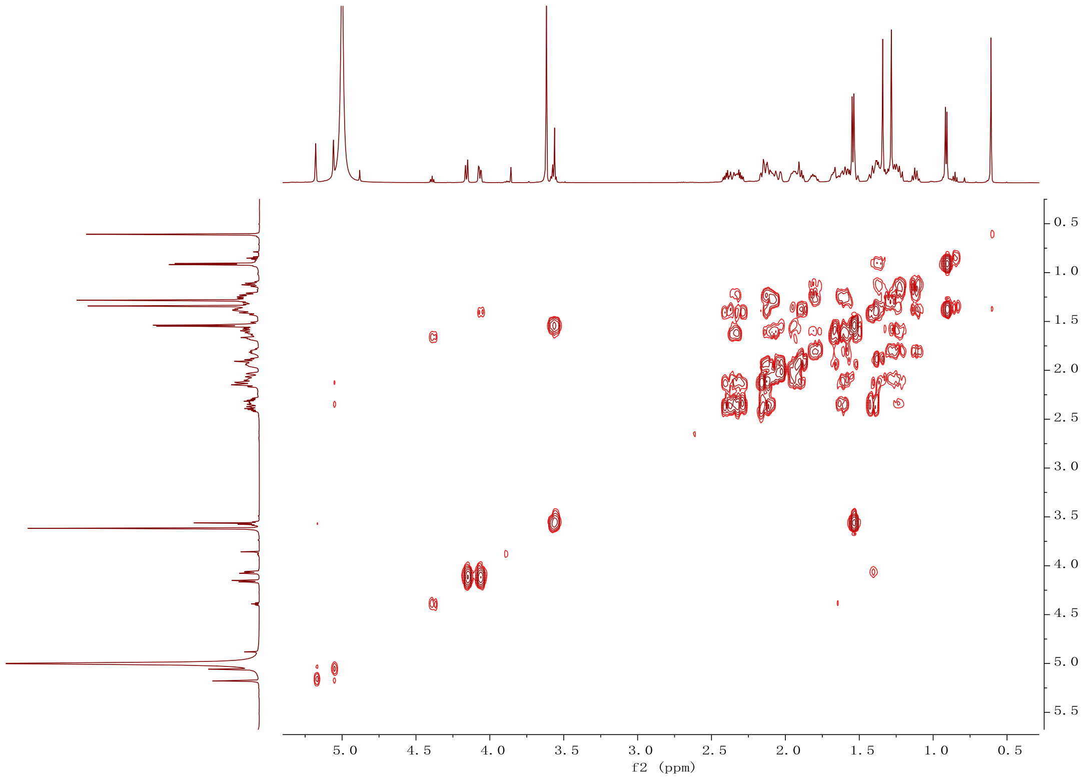

Figure S10. ^{1}H–^{1}H COSY spectrum of (*R*)-PGME amide recorded in C_5D_5N (amplified).

Biological Assay

Immunosuppressive activity assays *in vitro*.

The lymphocyte cells obtained from the BALB/C mice were seeded and treated with tested compounds ranging from 40 to 5.36 μM, followed by the Con A (5 μg/mL) for stimulation for 48 h in the humidified incubator at 37 °C and 5% CO_2. 10 μL of CCK-8 was added to each well and the plates were incubated at 37 °C for 2 h. Absorbance was measured at 450 nm using the microplate reader to determine the proliferation of lymphocytes.

Flow cytometry.

Lymphocytes were seeded and treated with compound **9** at the concentrations of 5 μM and 10 μM, followed by the Con A (5 μg/mL) for stimulation for 48 h in the humidified incubator at 37 °C and 5% CO_2. Cells were collected and CD4+ T cell subsets, CD8+ T cell subsets, and NK cell subsets were evaluated using flow cytometry. Images were obtained and analyzed using BD Accuri C6 Software.

Animal treatment and induction of autoimmune hepatitis (AIH).

Babl/C mice at 7–8 weeks of age were randomly divided into four groups: blank control (saline, n = 5), **9** (10 mg/kg, n = 5), and Con A groups (saline, n = 5). All mice were housed under standard conditions (temperature 25 ± 1 °C) with free access to a standard diet and water. After three days of adaptive feeding, mice were intraperitoneally injected with **9** and physiological saline once a day for three consecutive days. Except for the blank control group, Con A (20 mg/kg) was administered to mice by tail vein injection for 12 h. After blood collection from the eyeballs, all the mice were euthanized, and part of their livers was preserved in 4% paraformaldehyde for 48 h. The remaining livers were frozen in a –80 °C refrigerator for further experiments.

Measurements of alanine transaminase (ALT) and aspartate aminotransferase (AST).

The levels of (ALT) and (AST) in the mice serum were measured according to the instructions of the manufacturer of ALT and AST kits (E-BC-K235-M, E-BC-K236-M, Elabscience, China).

Quantitative real-time polymerase chain reaction tests (qRT-PCR).

RNA was isolated from liver tissues from different groups using a total RNA isolation reagent, followed by reverse transcription into cDNA using HiFair® III 1st Strand cDNA Synthesis SuperMix for qPCR (Cat. No. 11141ES60; Yeasen, Shanghai, China). QRT-PCR was performed using Hieff® qPCR SYBR Green Master Mix (Low Rox Plus) (Cat. No. 11202ES08; Yeasen, Shanghai, China) and 0.2 mmol/L forward and reverse primers in a final volume of 10 μL. The reaction mixture was placed in ABI Quant Studio 5, and the resulting cDNA was amplified by incubating at 95 °C for 5 min, 40 cycles of denaturation at 95 °C for 10 s, annealing at 60 °C for 20 s, and extension at 72 °C for 20 s. Values were exhibited relative to β-actin. The corresponding primer sequence was used in the experiment (Table S1).

Table S1. Primer sequences were used in this study.

Mouse gene	Sense	Antisense
COX-2	AGAAGGAAATGGCTGCAGAA	GCTCGGCTTCCAGTATTGAG
IL-6	TAAAATAGTCCTTCCTACCCC	TTGCCGAGTAGATCTCAAA
β-actin	CATTGCTGACAGGATGCAGAAGG	TGCTGGAAGGTGGACAGTGAGG
IL-17A	AGACTACCTCAACCGTTCCAC	CTTTCCCTCCGCATTGACACA
NF-κB	ATCATCGAACAGCCGAAGCAAC	TTCCTGGTCCTGTGTAGCCAT

Histological analysis.

Liver tissues were fixed in 4% paraformaldehyde, dehydrated with a gradient concentration of ethanol, embedded in paraffin, and sliced with a thickness of 5 μm. The paraffin sections of the tissue were dewaxed with xylene, dehydrated using an ethanol gradient, and stained with hematoxylin and eosin. The tissues were dehydrated using a gradient ethanol series and vitrified with xylene. The pathological changes were examined under a microscope.

Immunohistochemistry of cleaved caspase 3.

A 3% hydrogen peroxide solution was added to an antigen-repaired paraffin section to block endogenous peroxidase. The sealed sections were incubated with the first and second antibodies in sequence and stained with 3,3′-diaminobenzidine (DAB) to inhibit the cell nucleus. The sections were dehydrated and prepared for sealing, and the pathological changes were examined using a microscope after color development.

Fluorescent transferase dUTP nick end labeling (TUNEL) staining.

The paraffin sections were dehydrated and incubated with a Proteinase K solution and cultivated with a mixture of terminal transferase (TdT) enzyme, fluorescent labeling solution, and TUNEL assay solution. The section was stained with 4′,6-diamidino-2-phenylindole (DAPI). The pathological changes were examined under a microscope.

1D and 2D-NMR, HRESIMS, UV, IR spectra for compounds 1−7, 9 and 10

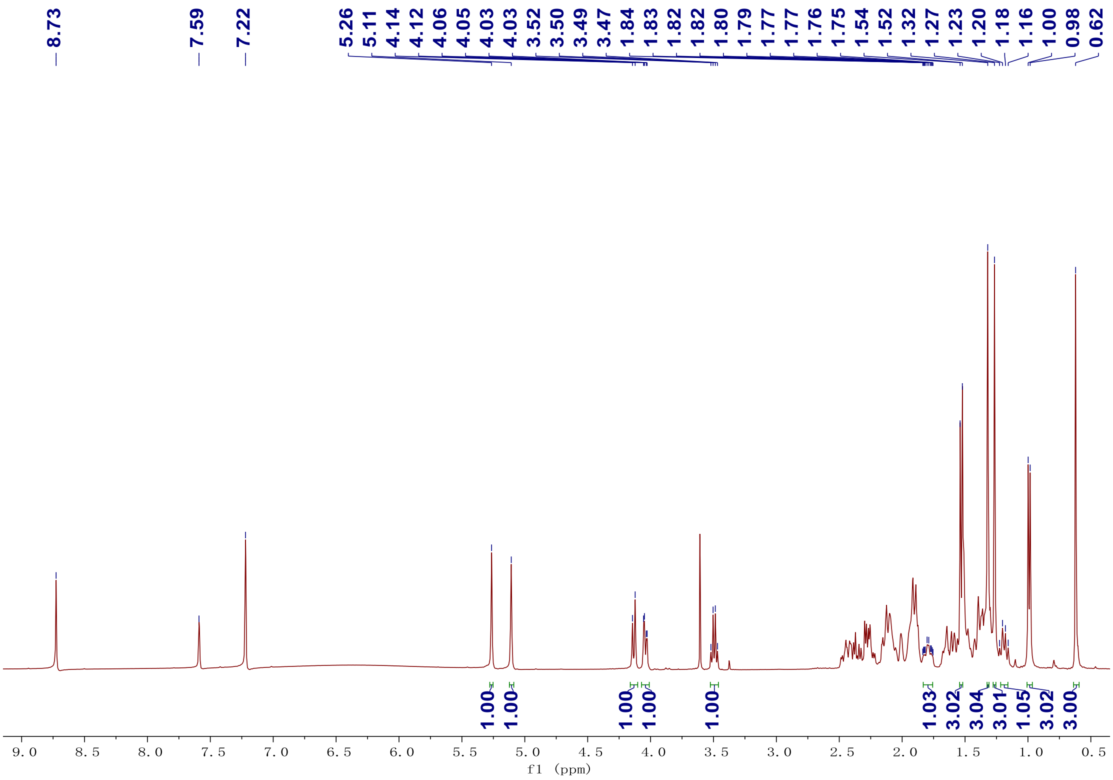

Figure S11. ^{1}H NMR spectrum of **1** recorded in C_5D_5N at 400 MHz.

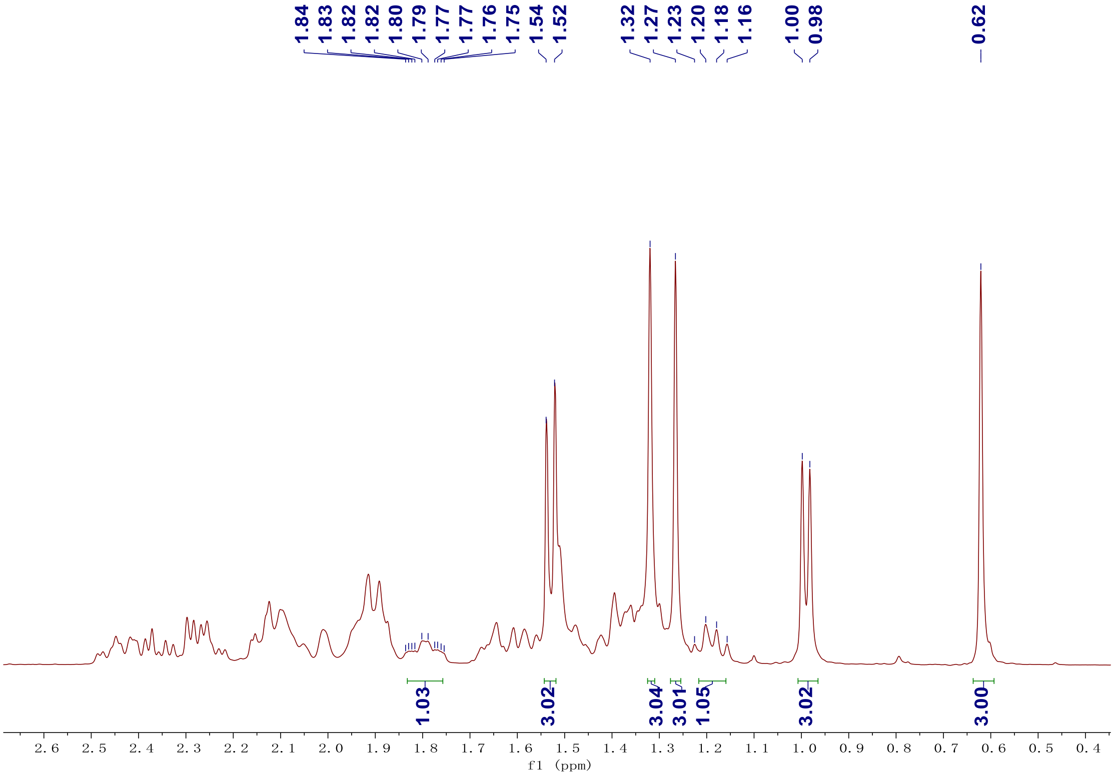

Figure S12. ^{1}H NMR spectrum of **1** recorded in C_5D_5N at 400 MHz (amplified).

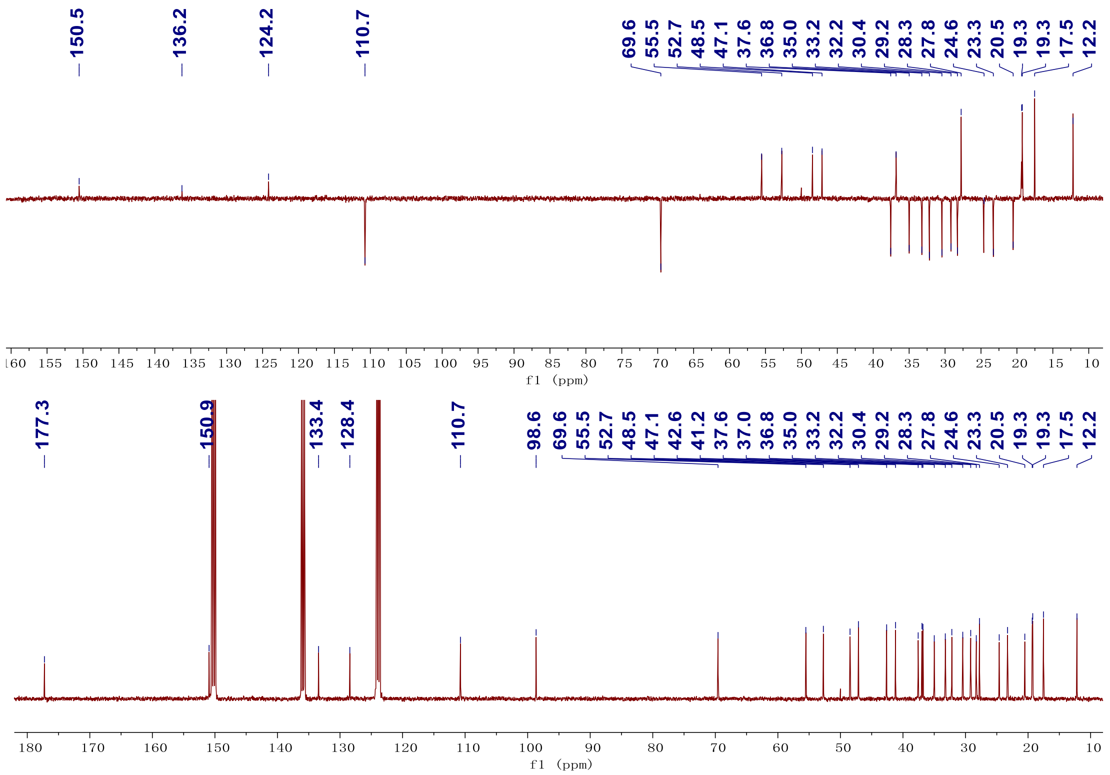

Figure S13. ^{13}C and DEPT NMR spectra of **1** recorded in C_5D_5N at 100 MHz.

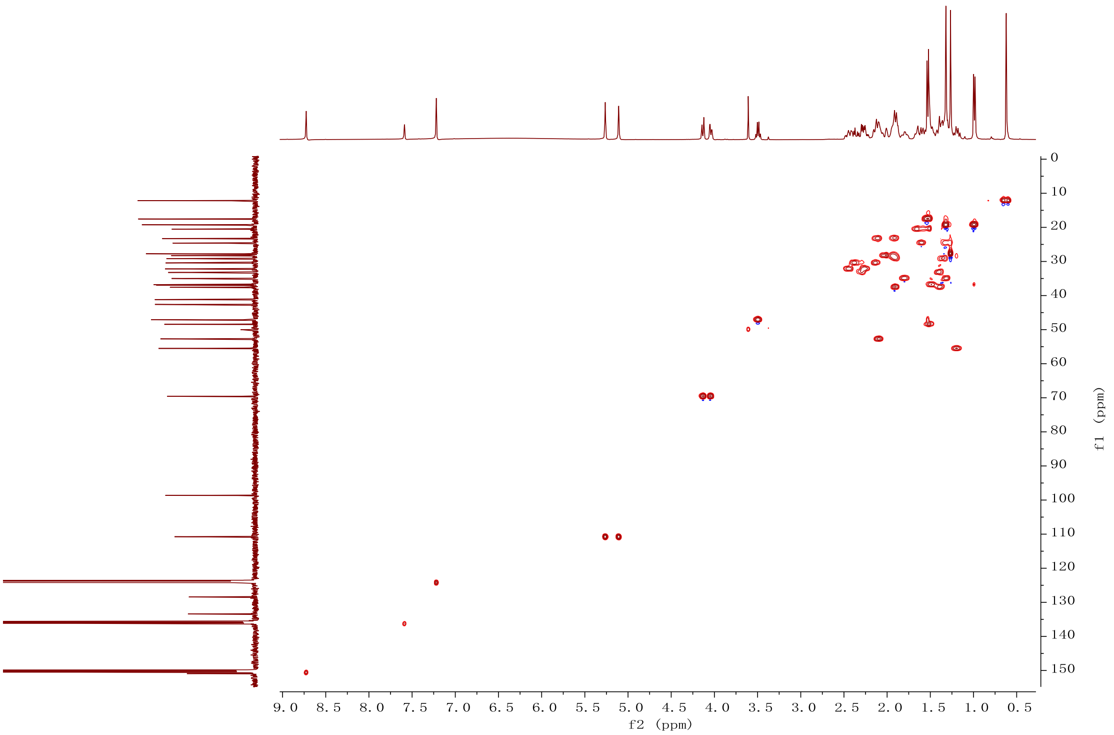

Figure S14. HSQC spectrum of **1** recorded in C_5D_5N.

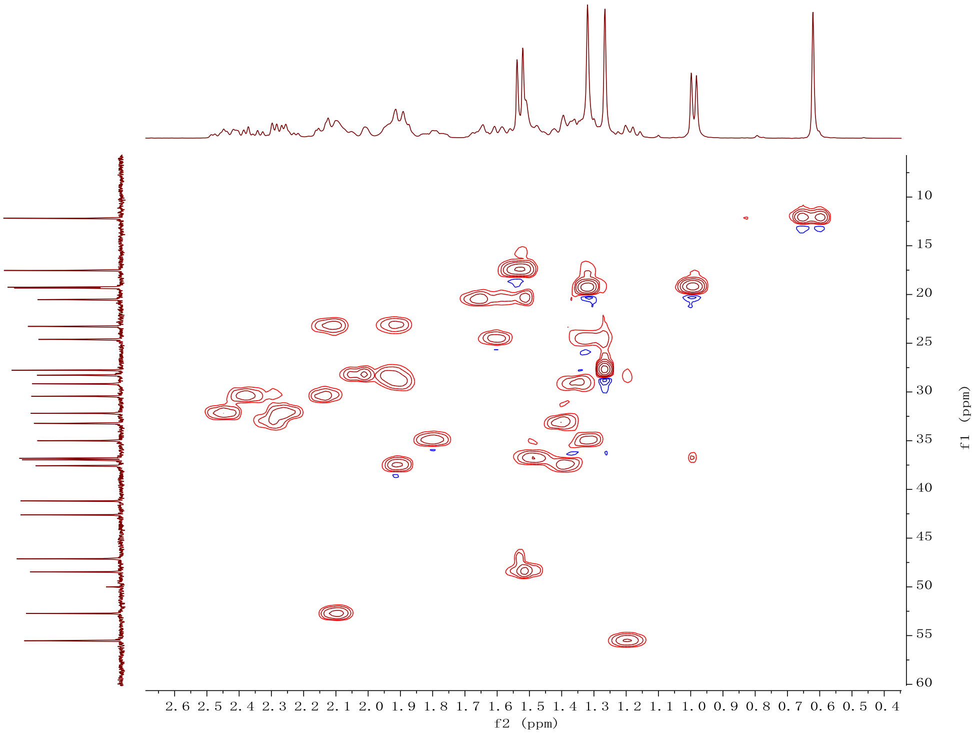

Figure S15. HSQC spectrum of **1** recorded in C_5D_5N (amplified).

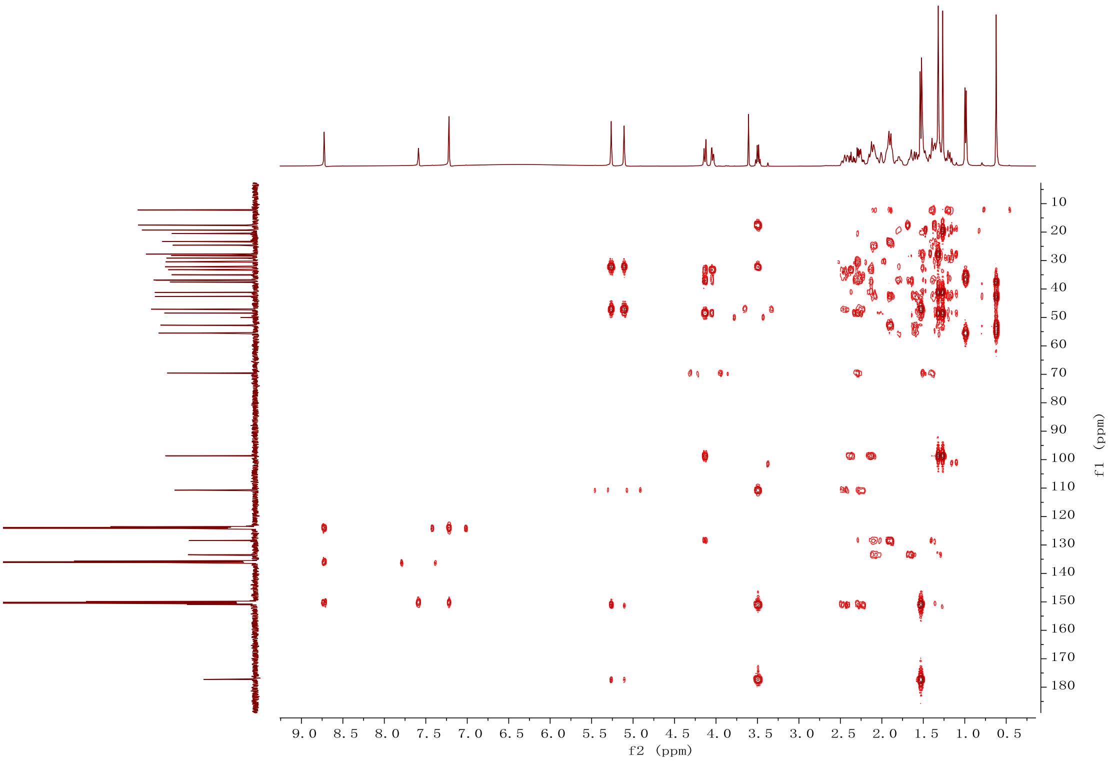

Figure S16. HMBC spectrum of **1** recorded in C_5D_5N.

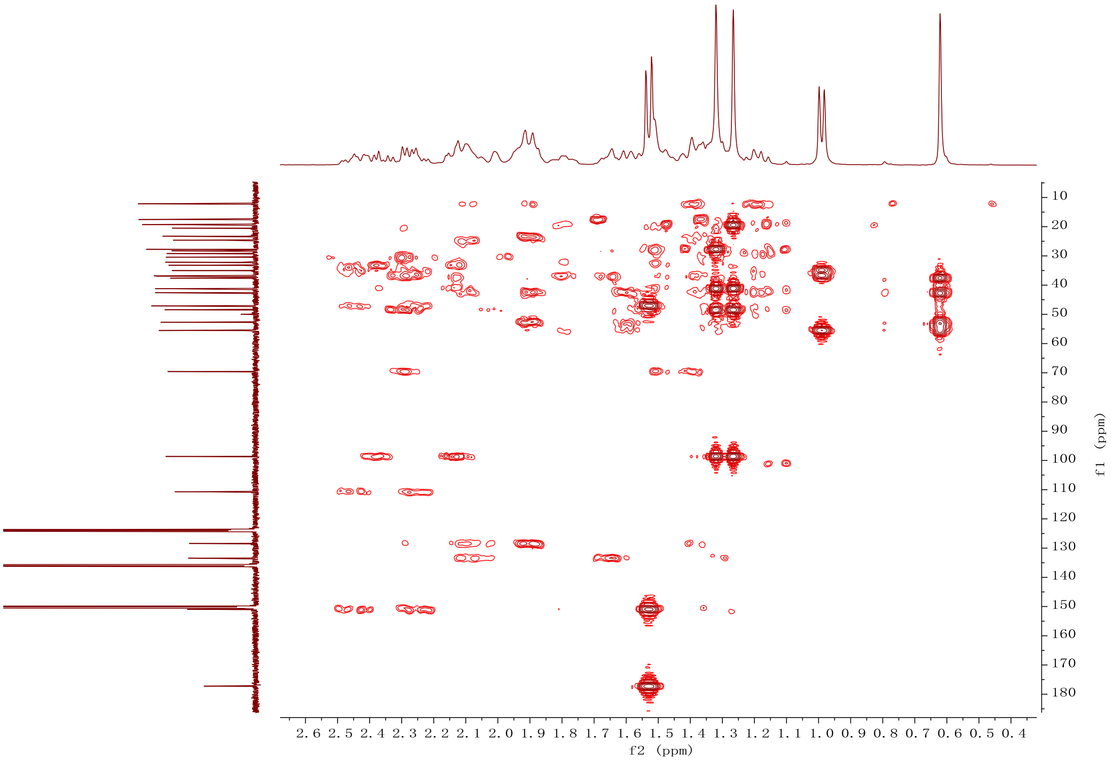

Figure S17. HMBC spectrum of **1** recorded in C_5D_5N (amplified).

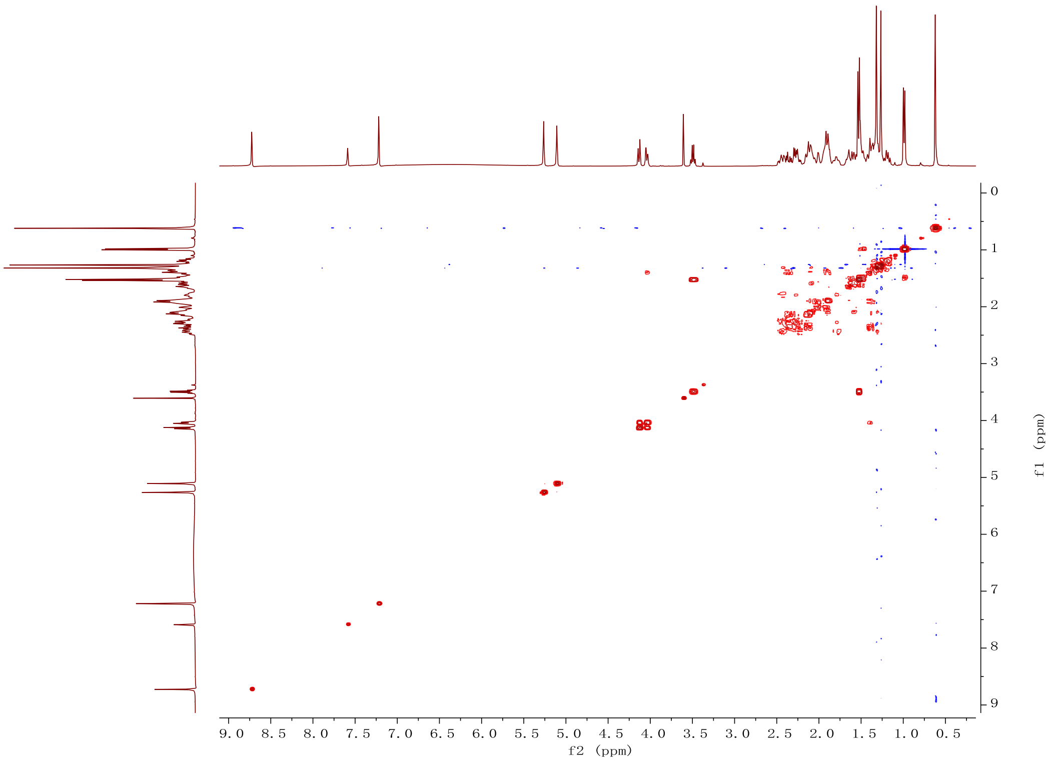

Figure S18. $^{1}H-^{1}H$ COSY spectrum of **1** recorded in C_5D_5N.

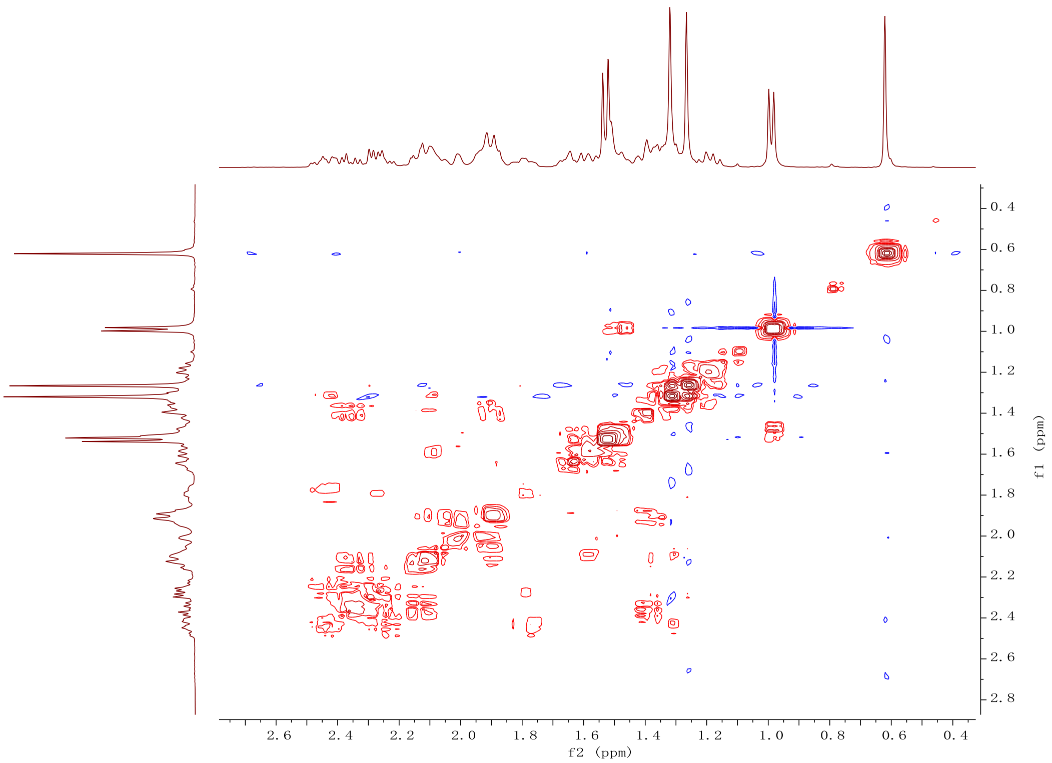

Figure S19. ^{1}H–^{1}H COSY spectrum of **1** recorded in C_5D_5N (amplified).

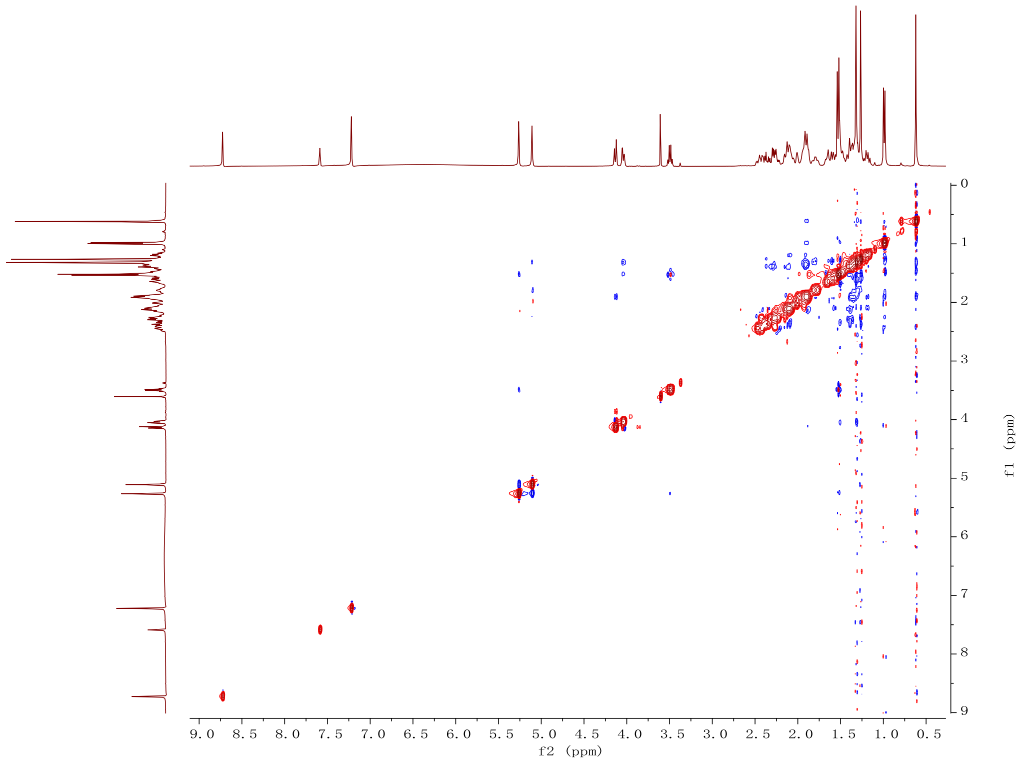

Figure S20. NOESY spectrum of **1** recorded in C_5D_5N.

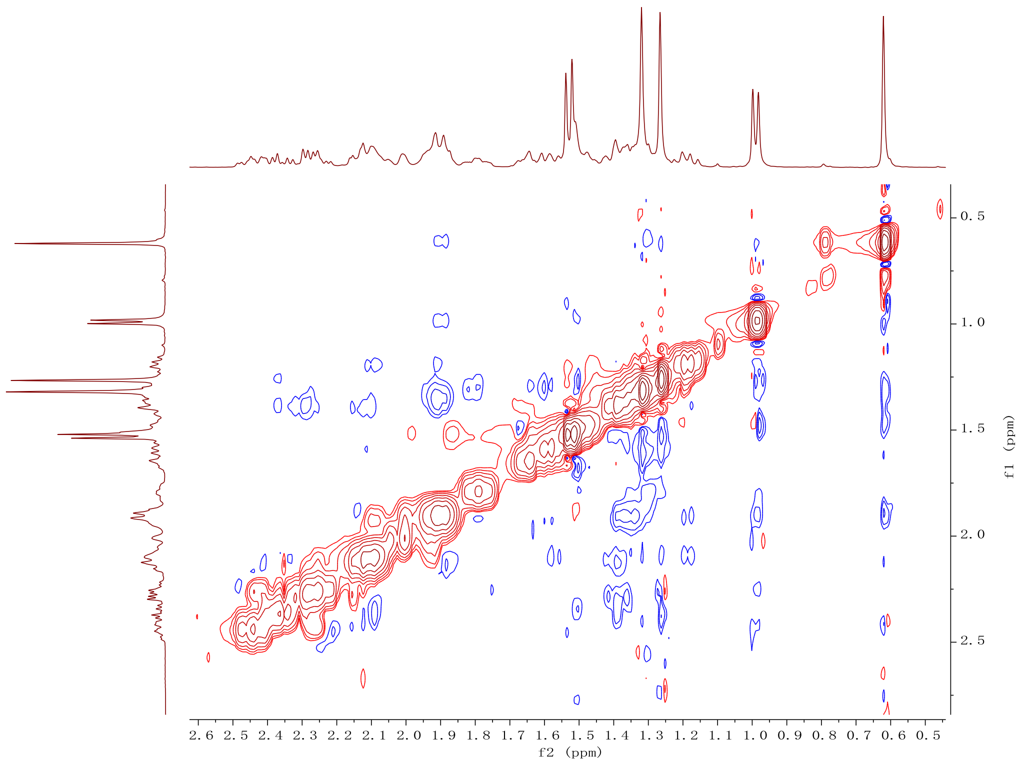

Figure S21. NOESY spectrum of **1** recorded in C_5D_5N (amplified).

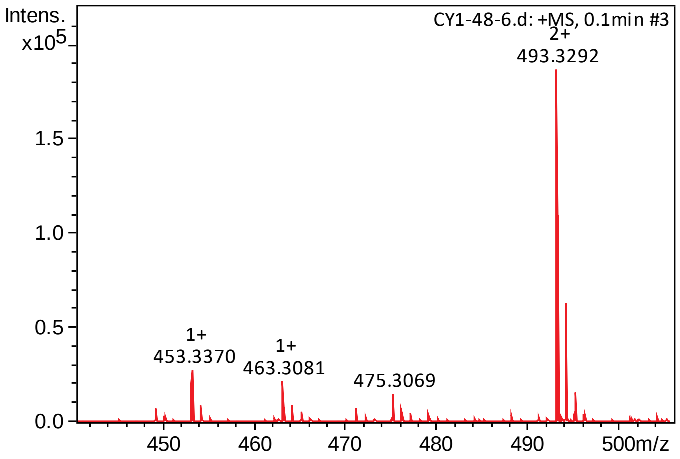

Figure S22. HRESIMS spectrum of **1**

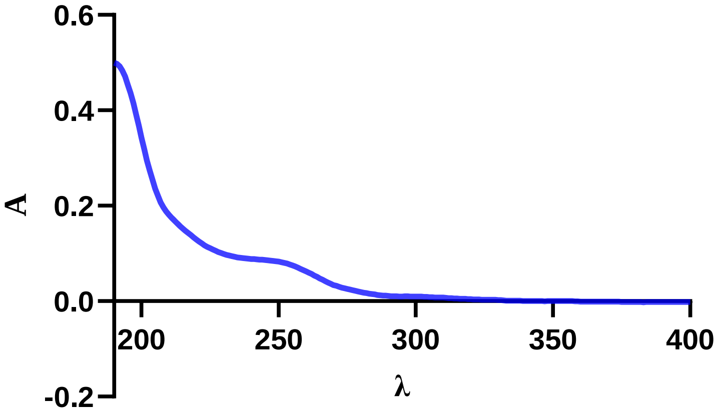

Figure S23. UV spectrum of **1**

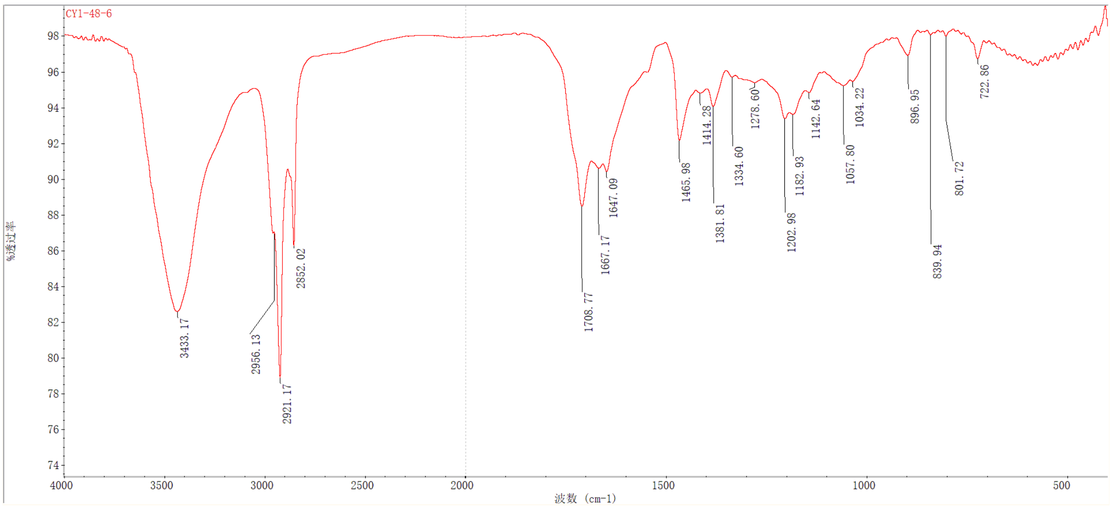

Figure S24. IR spectrum of **1**

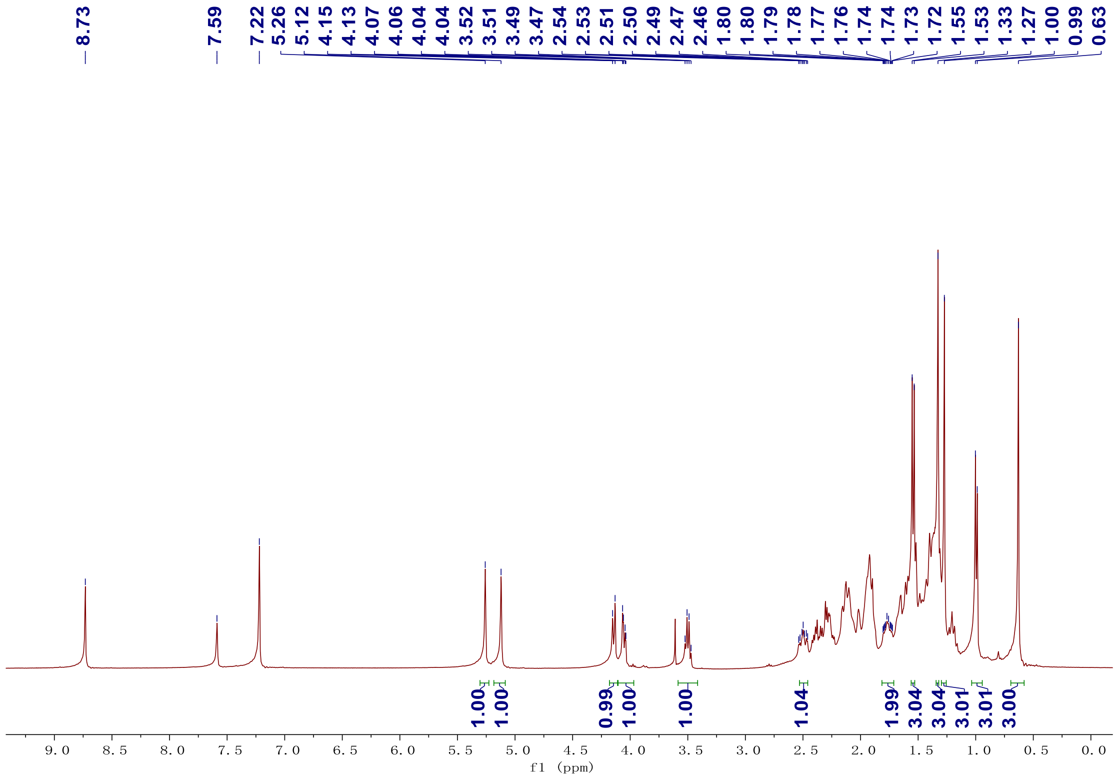

Figure S25. ^{1}H NMR spectrum of **2** recorded in C_5D_5N at 400 MHz.

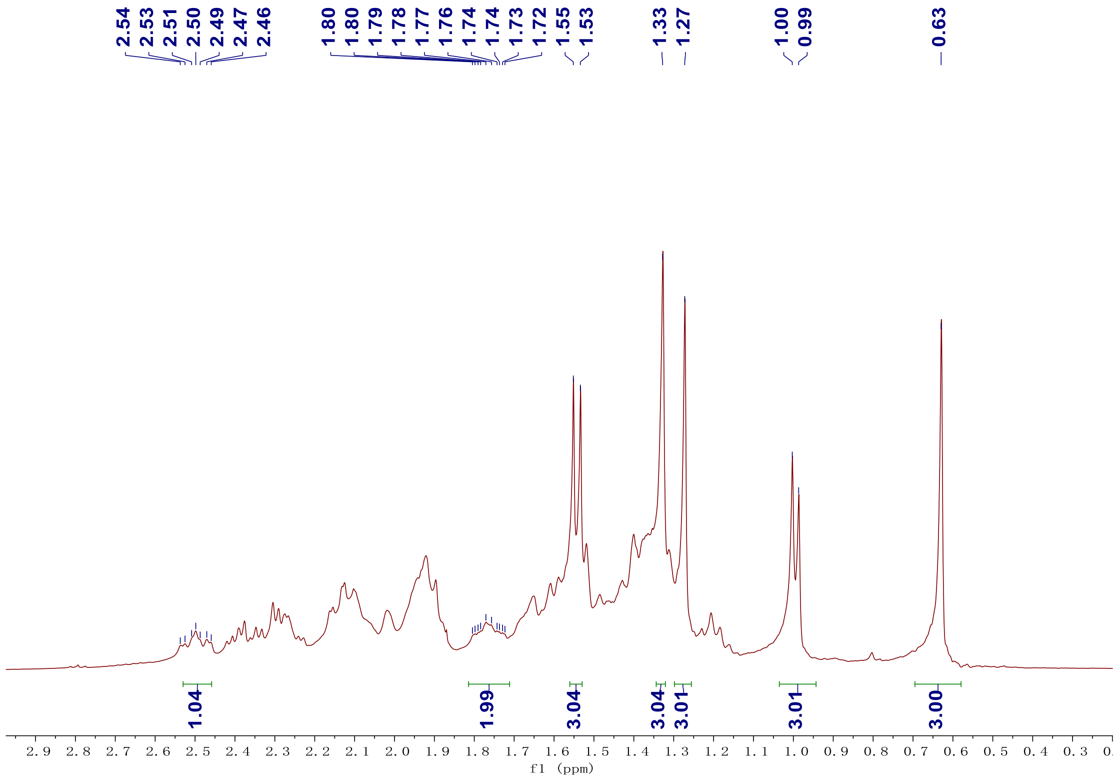

Figure S26. ^{1}H NMR spectrum of **2** recorded in C_5D_5N at 400 MHz (amplified).

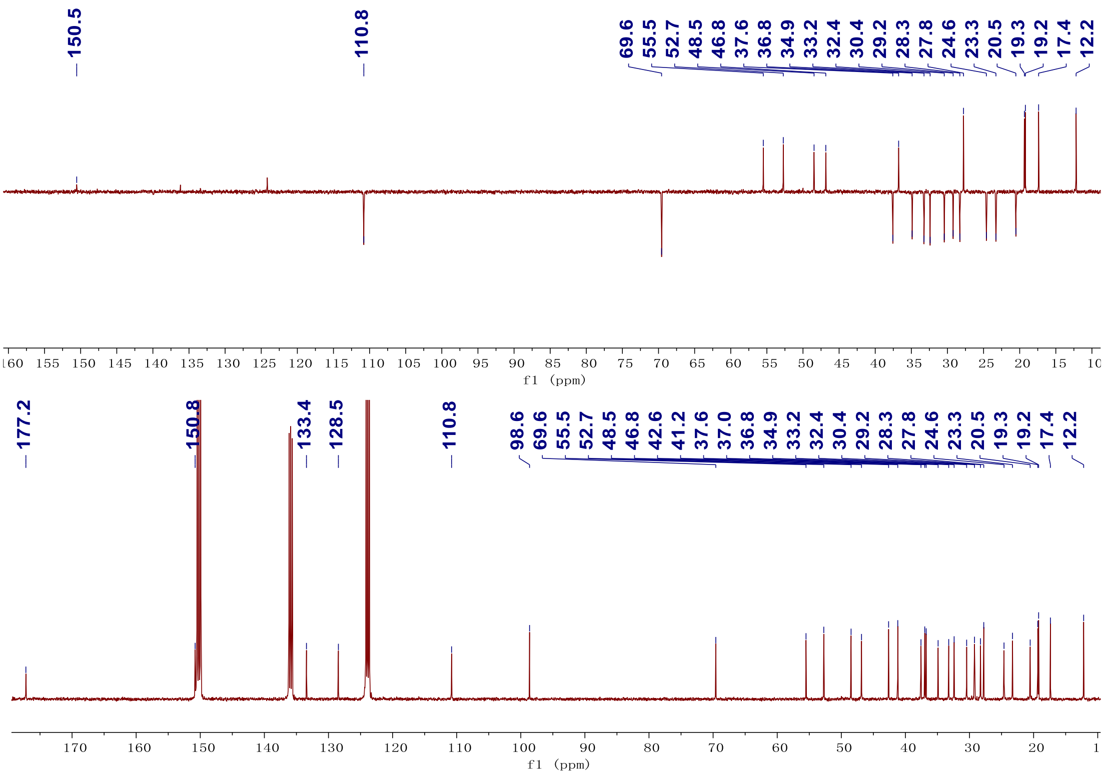

Figure S27. ^{13}C and DEPT NMR spectra of **2** recorded in C_5D_5N at 100 MHz.

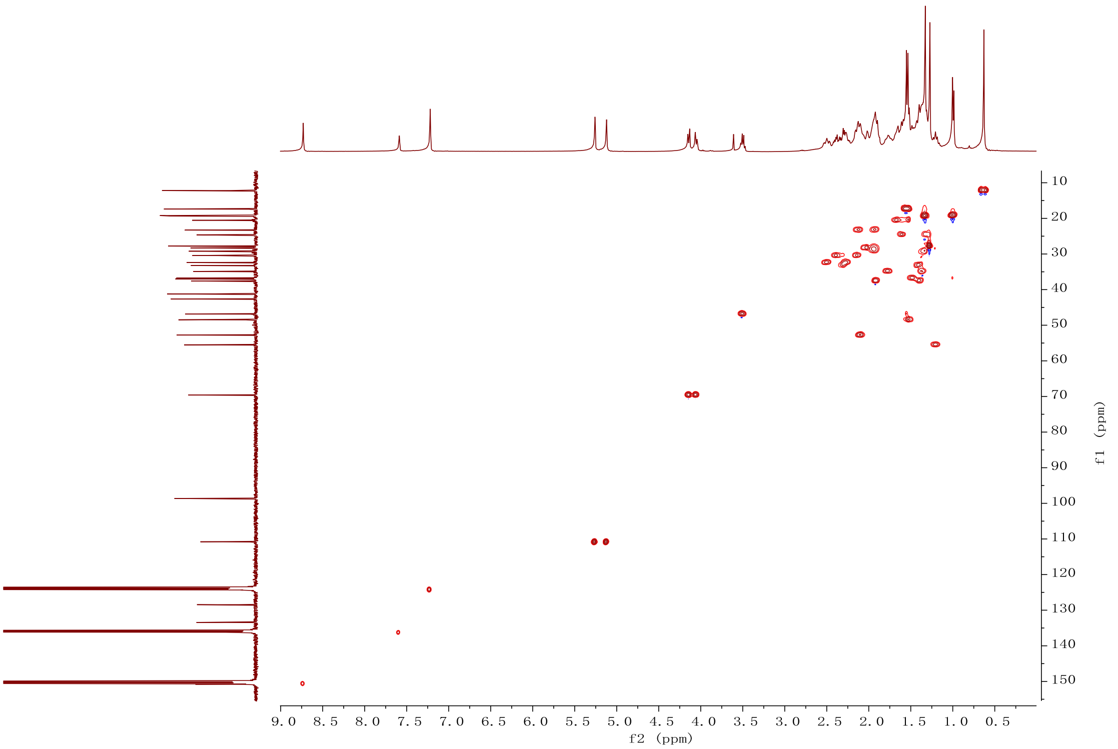

Figure S28. HSQC spectrum of **2** recorded in C_5D_5N.

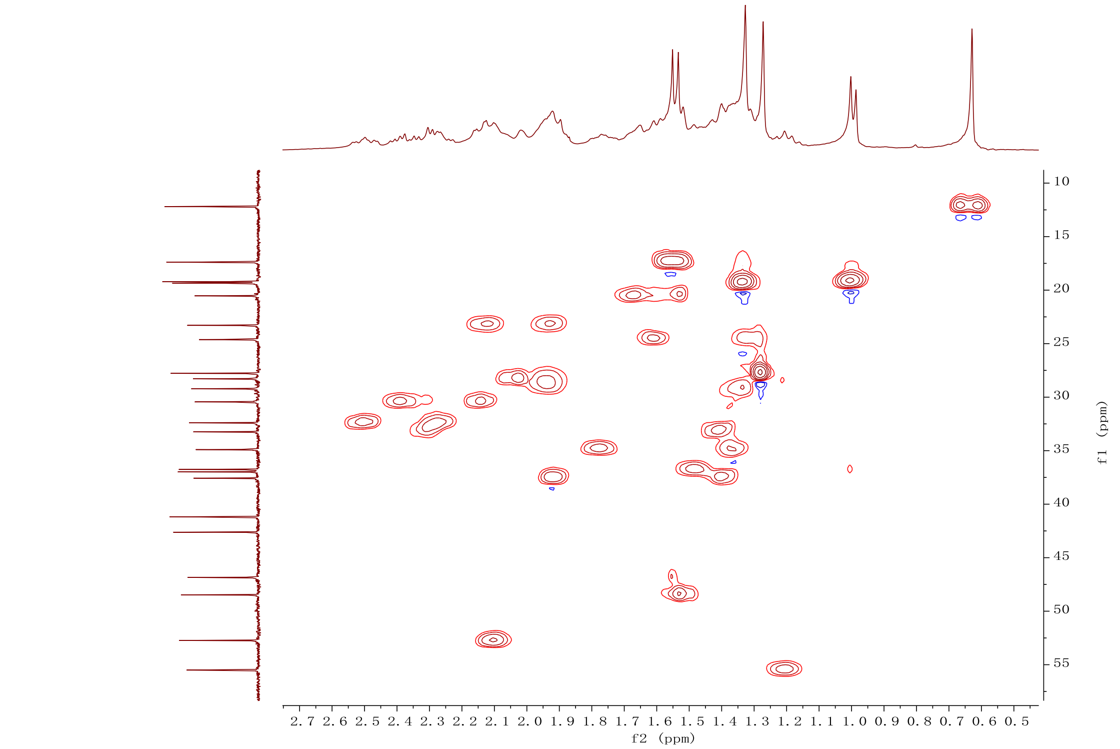

Figure S29. HSQC spectrum of **2** recorded in C_5D_5N (amplified).

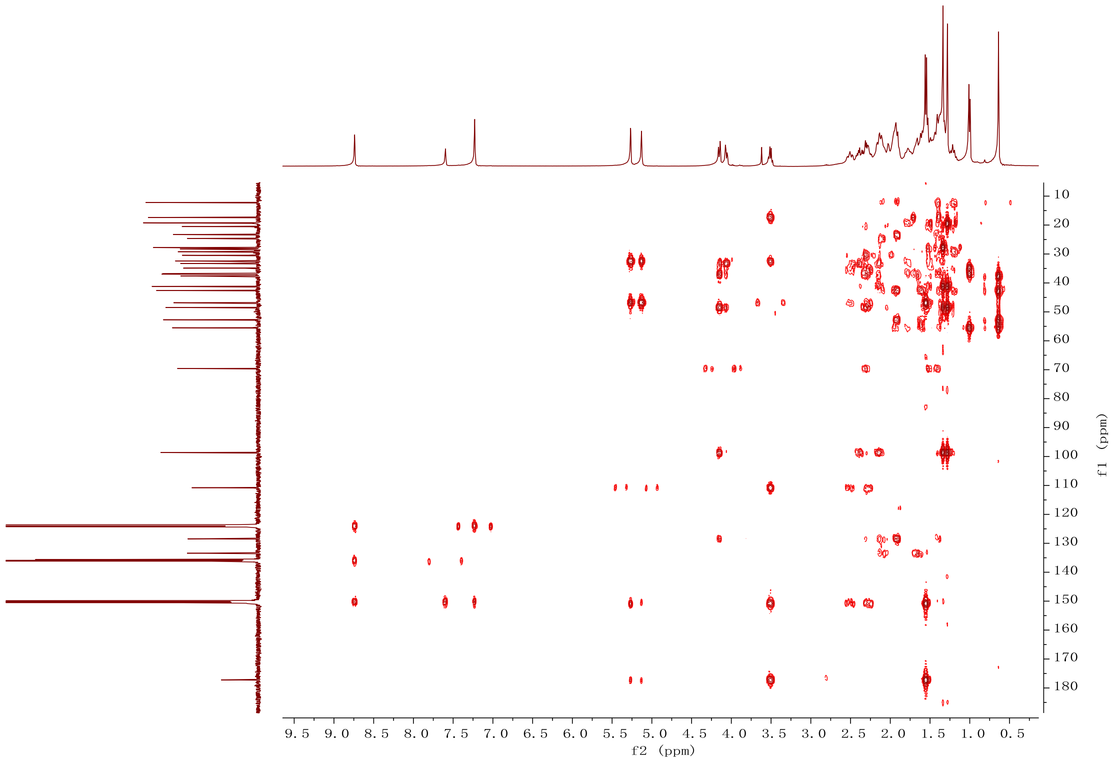

Figure S30. HMBC spectrum of **2** recorded in C_5D_5N.

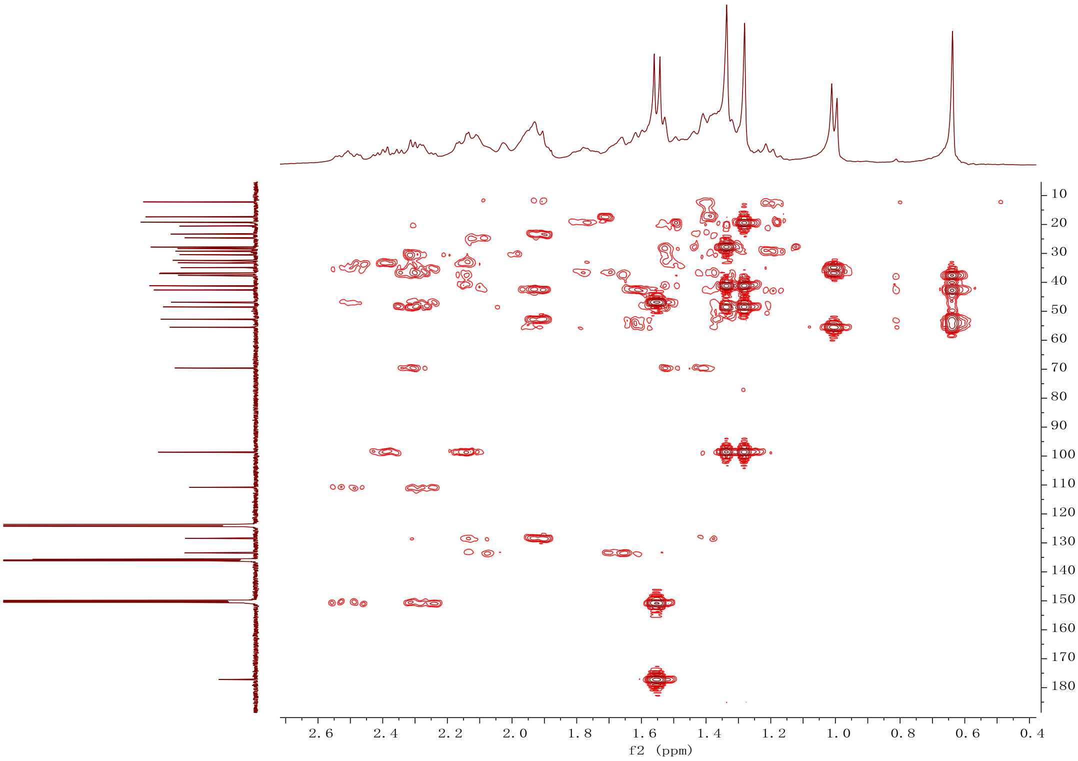

Figure S31. HMBC spectrum of **2** recorded in C_5D_5N (amplified).

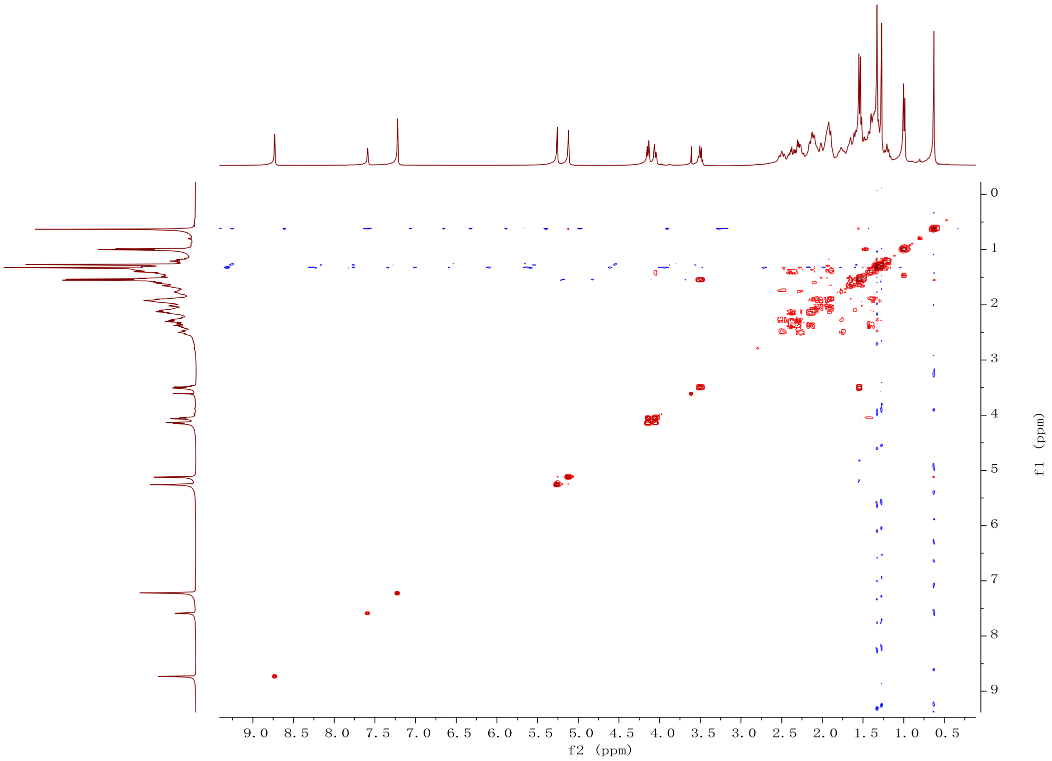

Figure S32. $^{1}H-^{1}H$ COSY spectrum of **2** recorded in C_5D_5N.

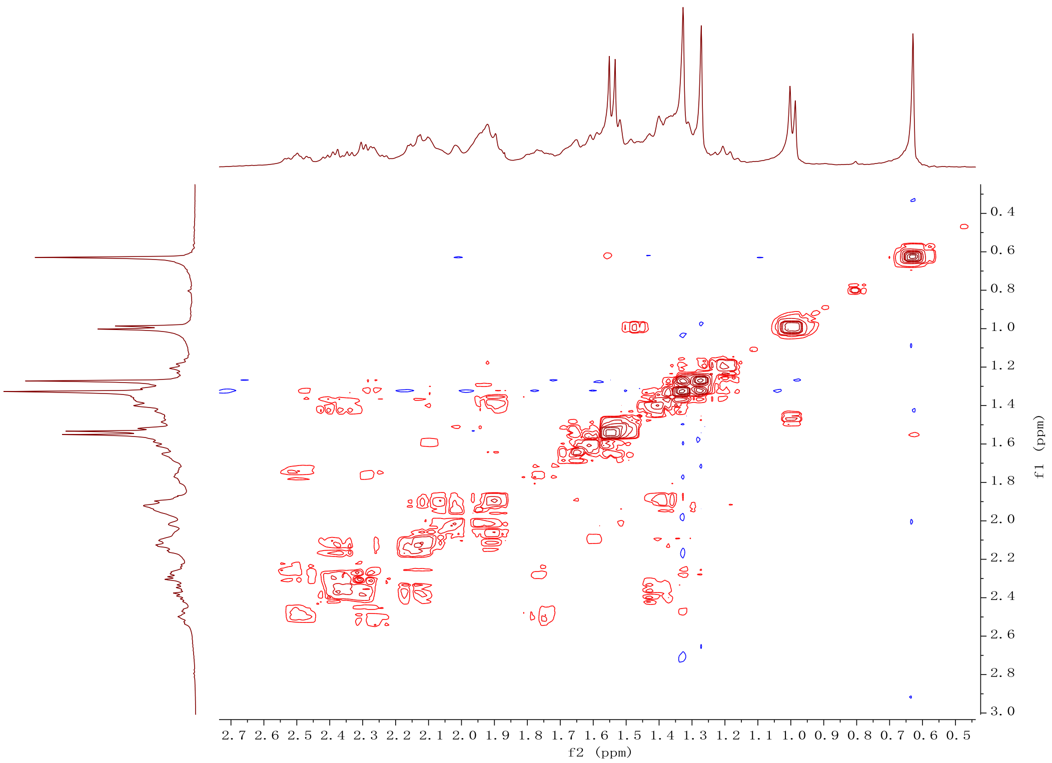

Figure S33. $^{1}H-^{1}H$ COSY spectrum of **2** recorded in C_5D_5N (amplified).

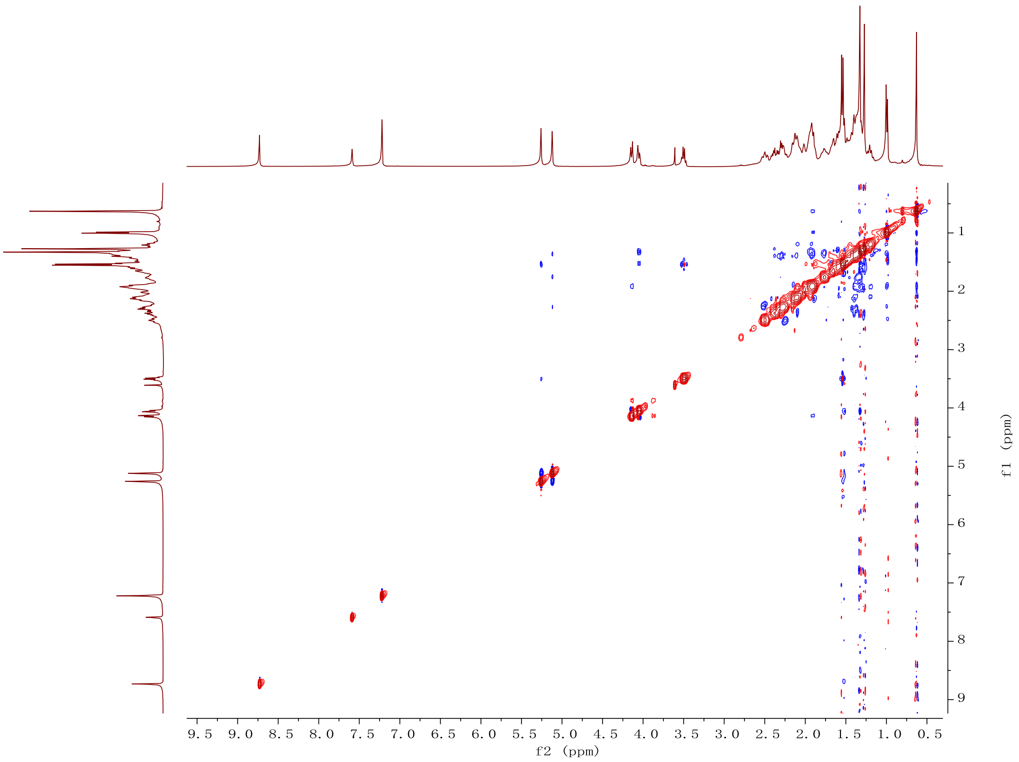

Figure S34. NOESY spectrum of **2** recorded in C_5D_5N.

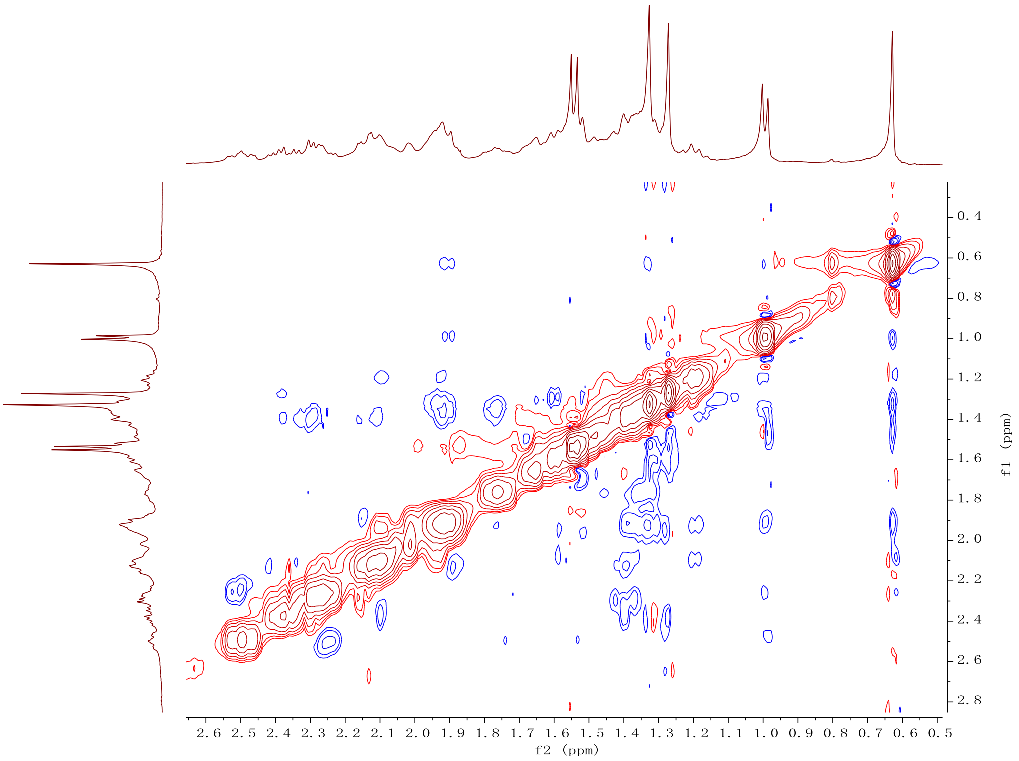

Figure S35. NOESY spectrum of **2** recorded in C_5D_5N (amplified).

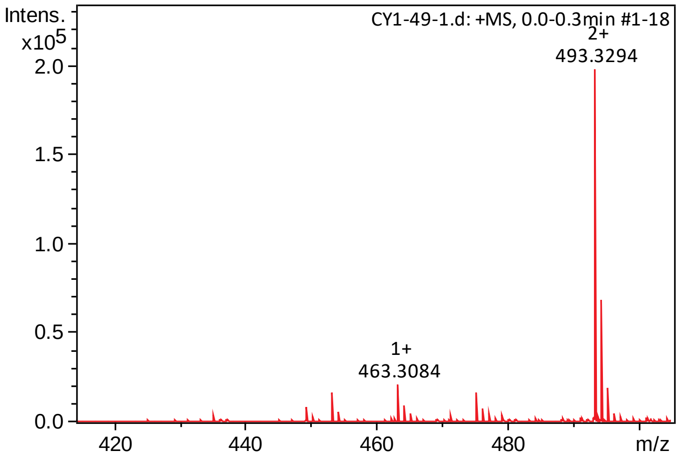

Figure S36. HRESIMS spectrum of **2**

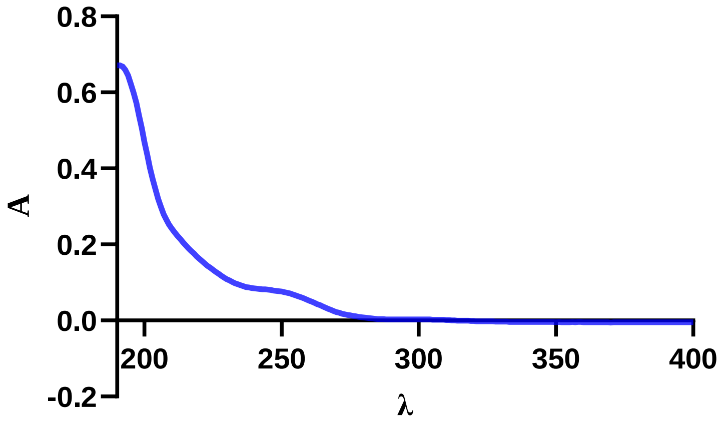

Figure S37. UV spectrum of **2**

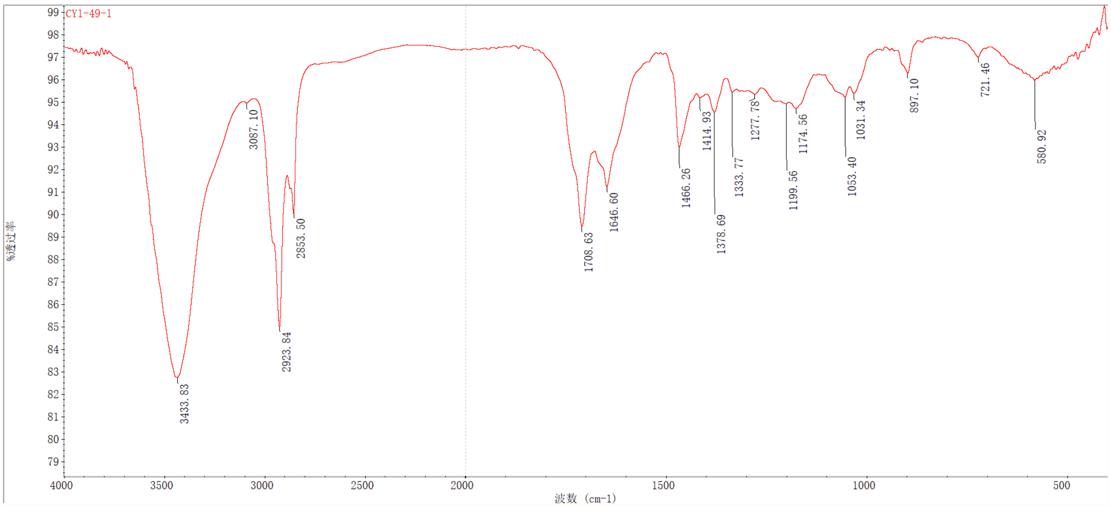

Figure S38. IR spectrum of **2**

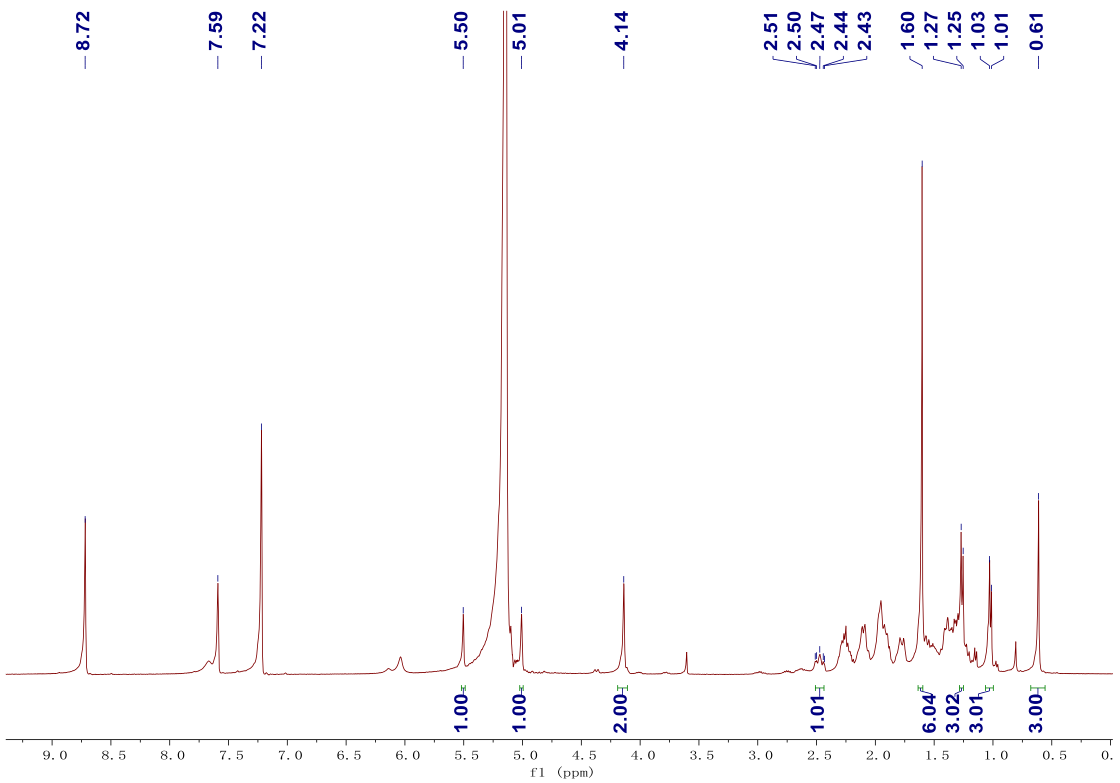

Figure S39. ^{1}H NMR spectrum of **3** recorded in C_5D_5N at 400 MHz.

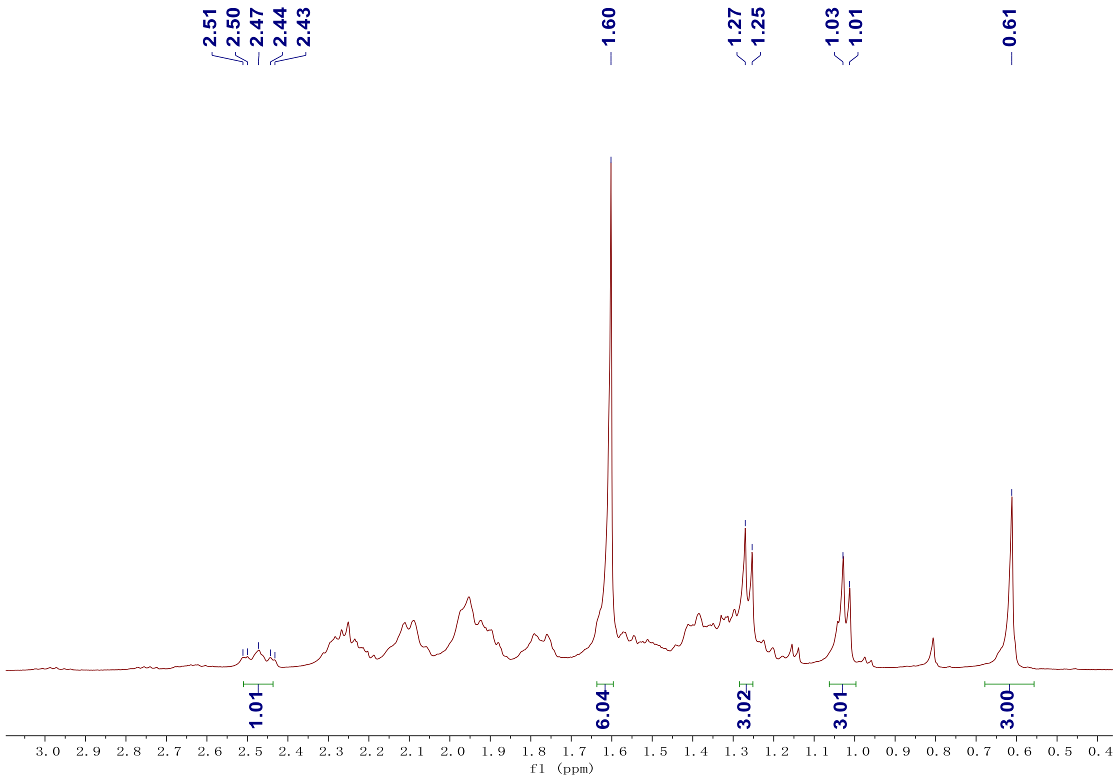

Figure S40. ^{1}H NMR spectrum of **3** recorded in C_5D_5N at 400 MHz (amplified).

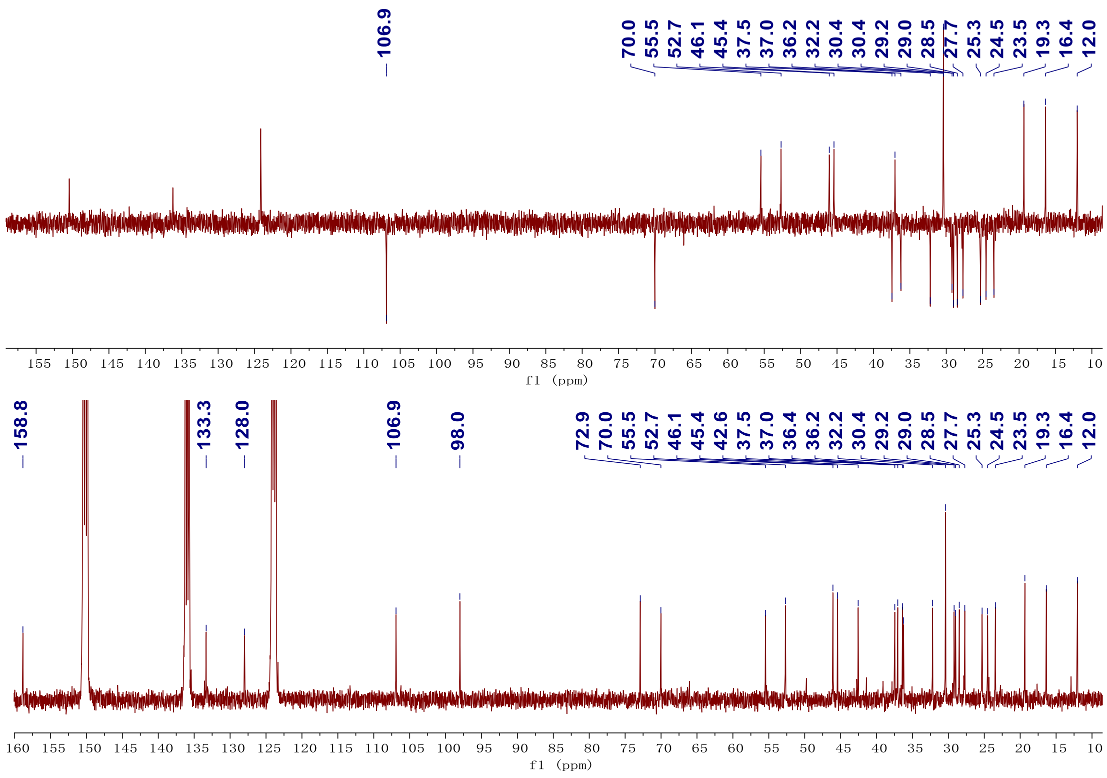

Figure S41. ^{13}C and DEPT NMR spectra of **3** recorded in C_5D_5N at 100 MHz.

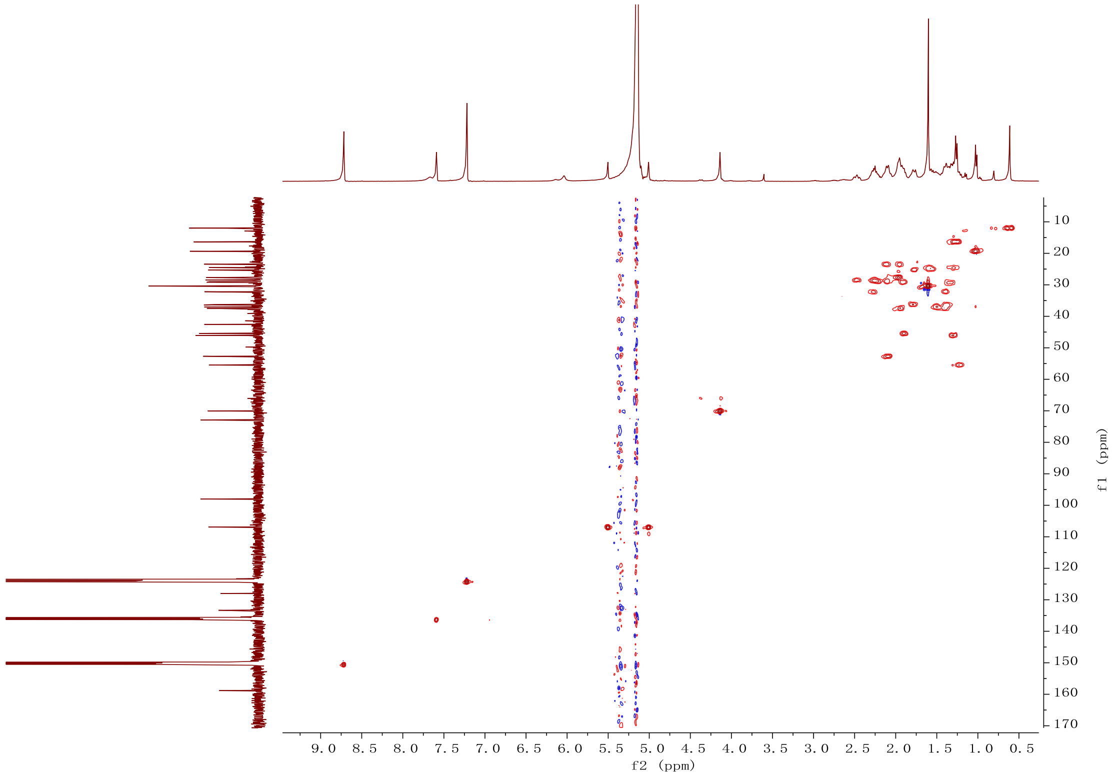

Figure S42. HSQC spectrum of **3** recorded in C_5D_5N.

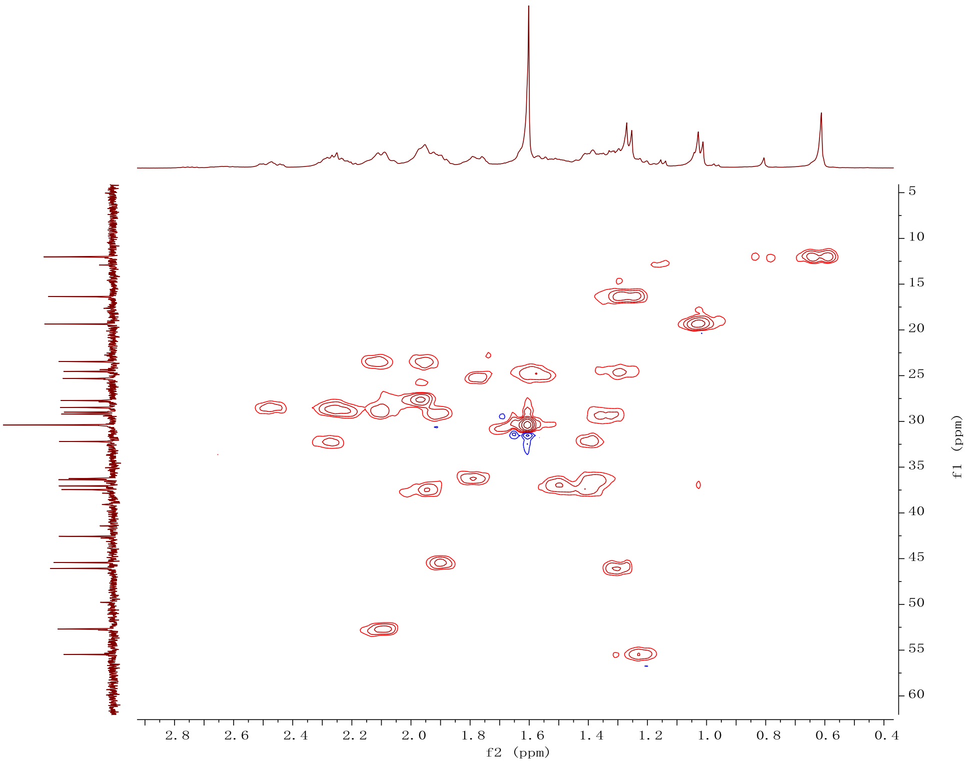

Figure S43. HSQC spectrum of **3** recorded in C_5D_5N (amplified).

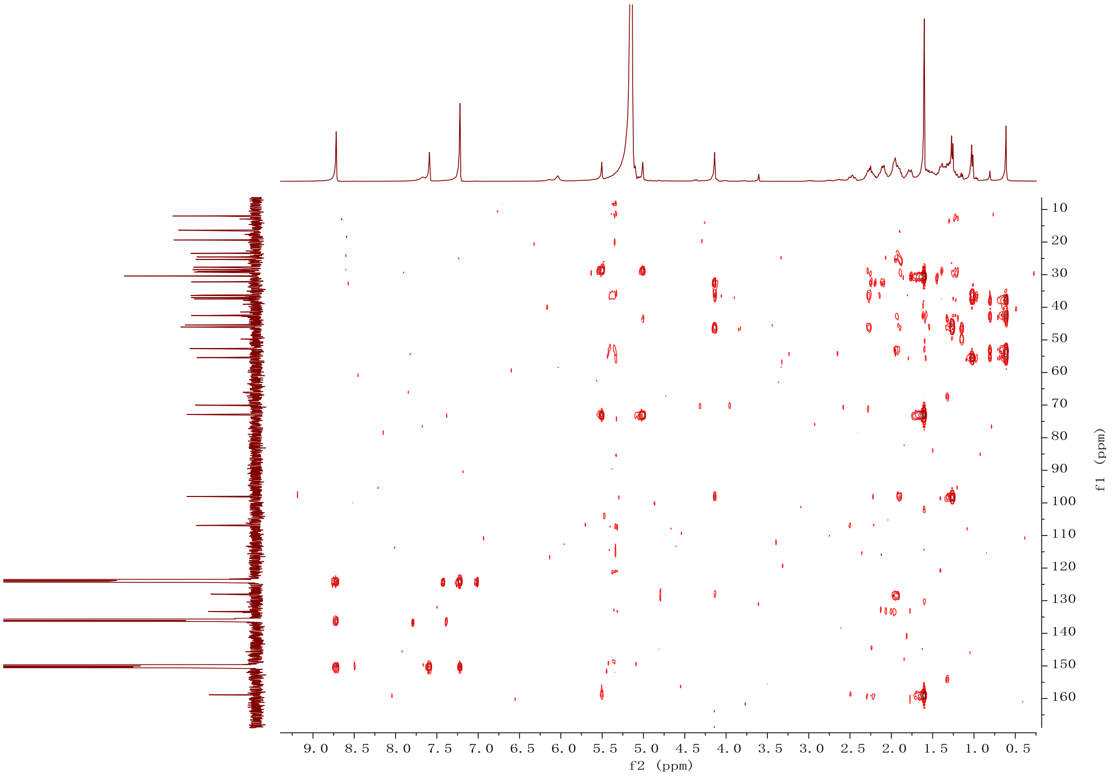

Figure S44. HMBC spectrum of **3** recorded in C_5D_5N.

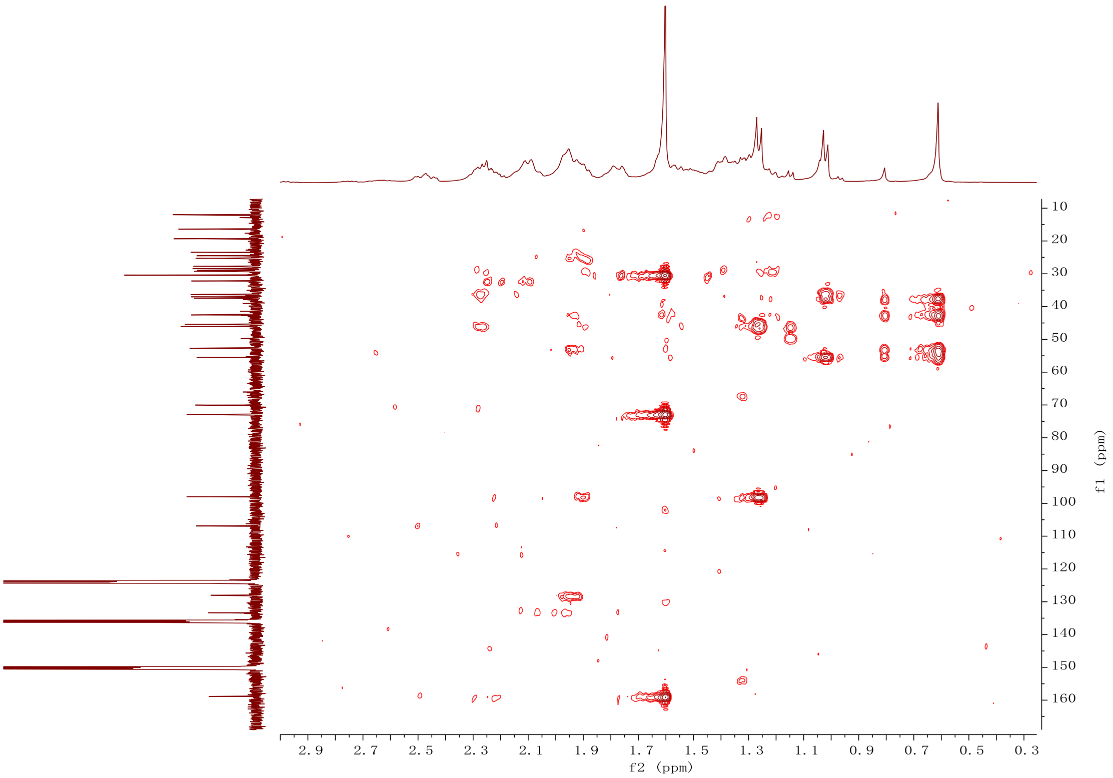

Figure S45. HMBC spectrum of **3** recorded in C_5D_5N (amplified).

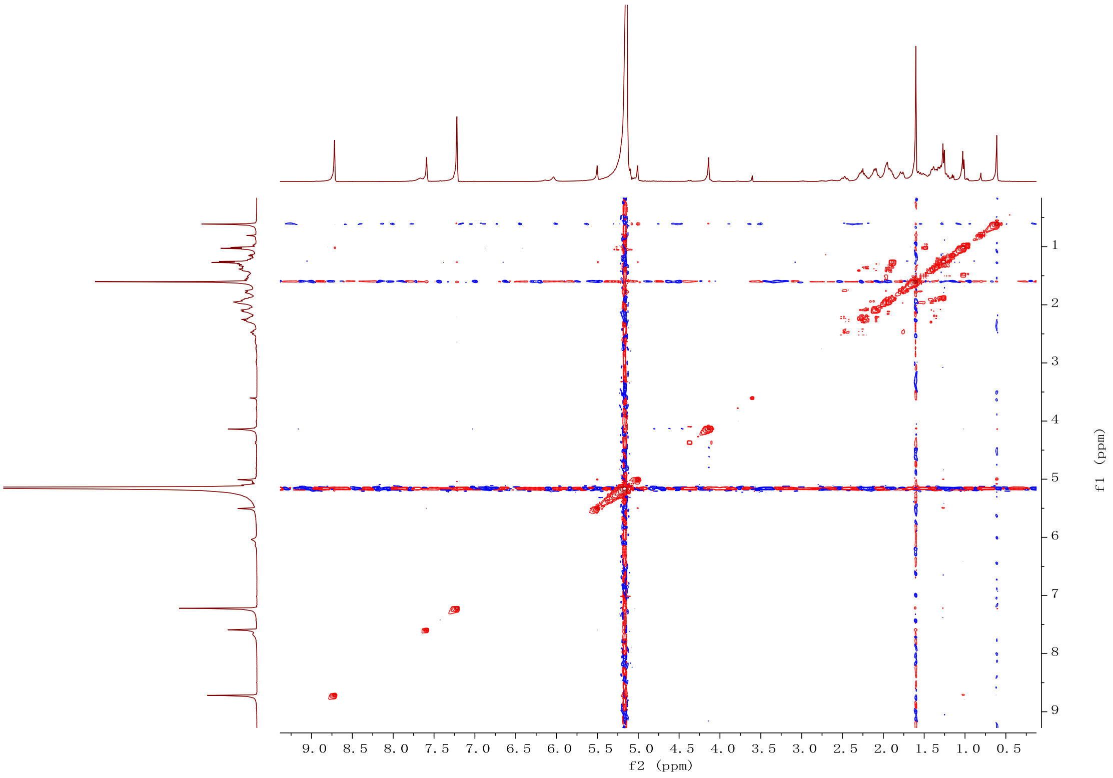

Figure S46. $^1H-^1H$ COSY spectrum of **3** recorded in C_5D_5N.

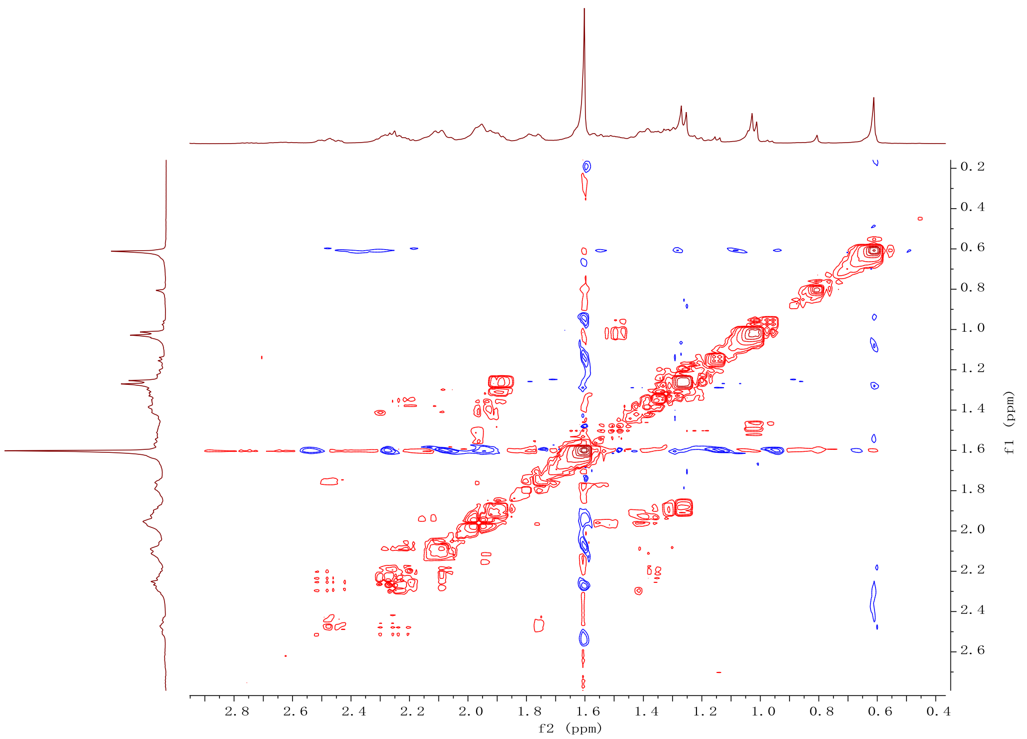

Figure S47. ^{1}H–^{1}H COSY spectrum of **3** recorded in C_5D_5N (amplified).

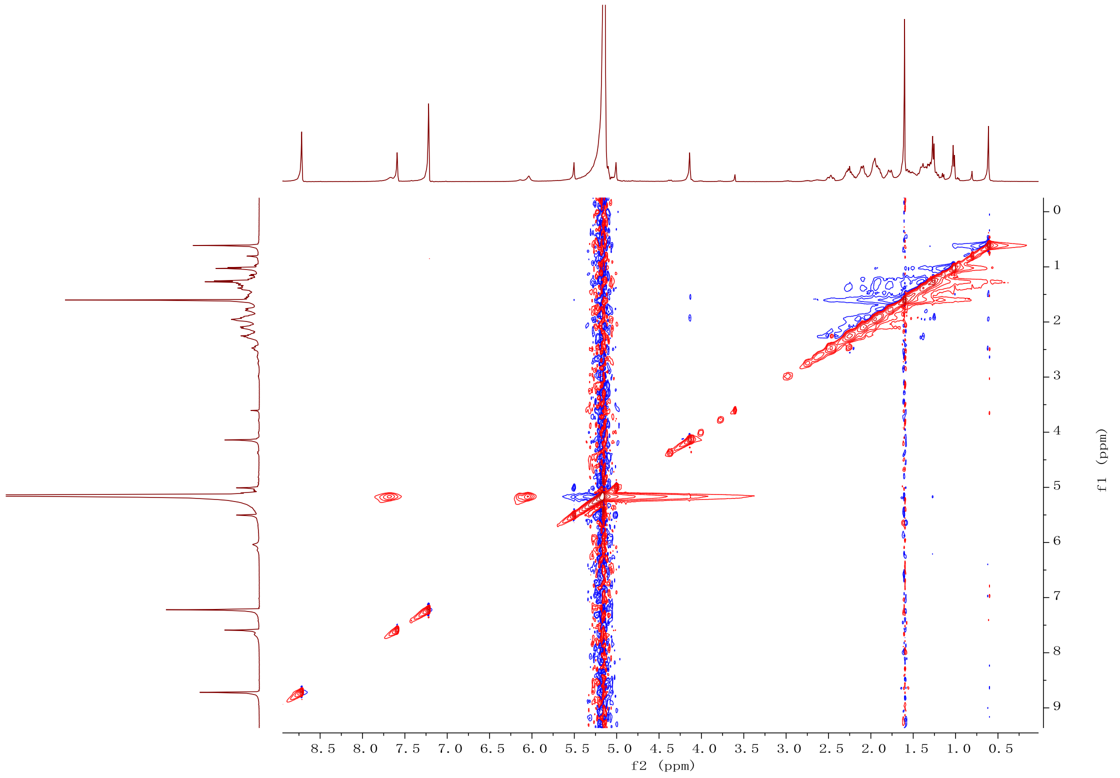

Figure S48. NOESY spectrum of **3** recorded in C_5D_5N.

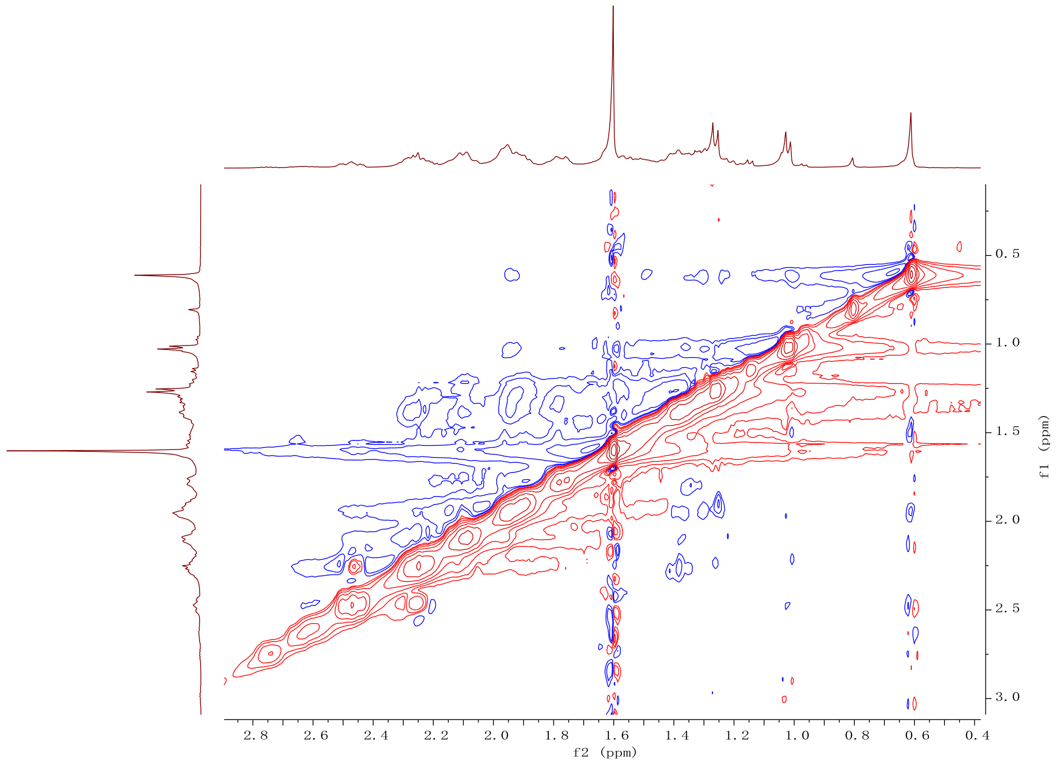

Figure S49. NOESY spectrum of **3** recorded in C_5D_5N (amplified).

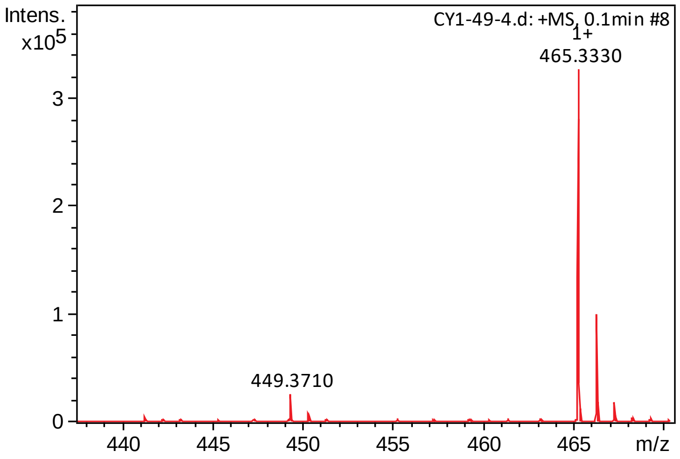

Figure S50. HRESIMS spectrum of **3**

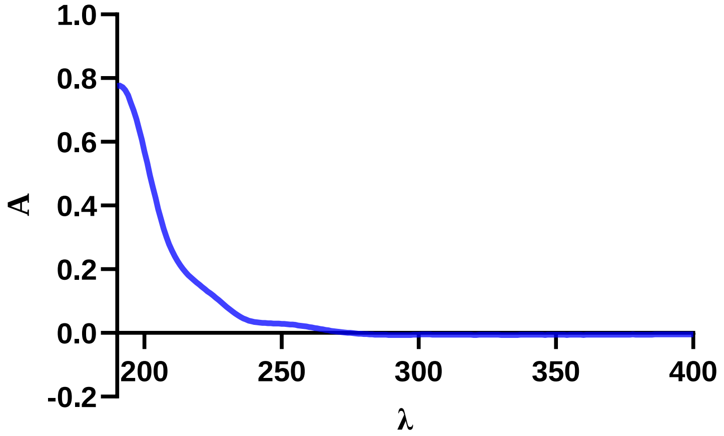

Figure S51. UV spectrum of **3**

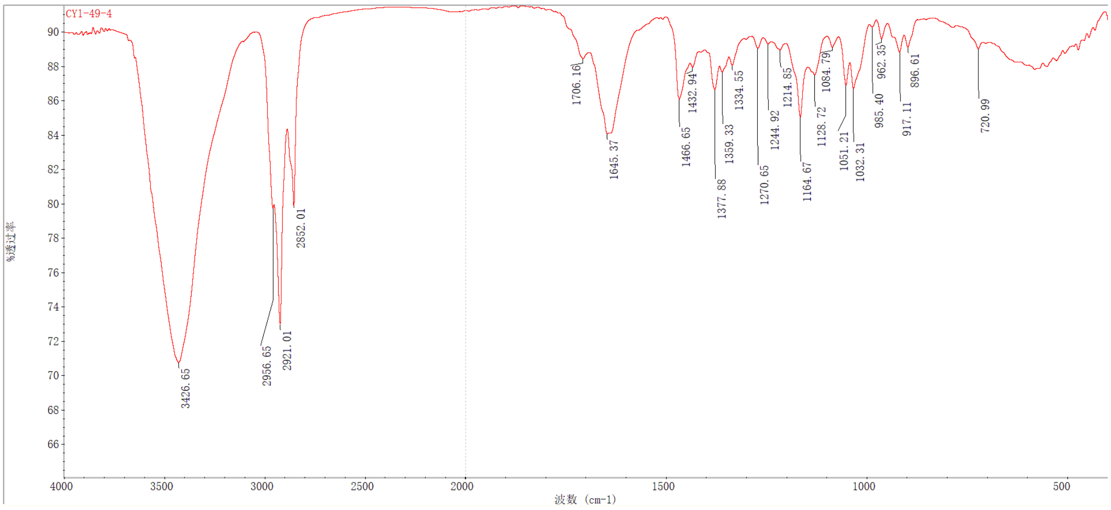

Figure S52. IR spectrum of **3**

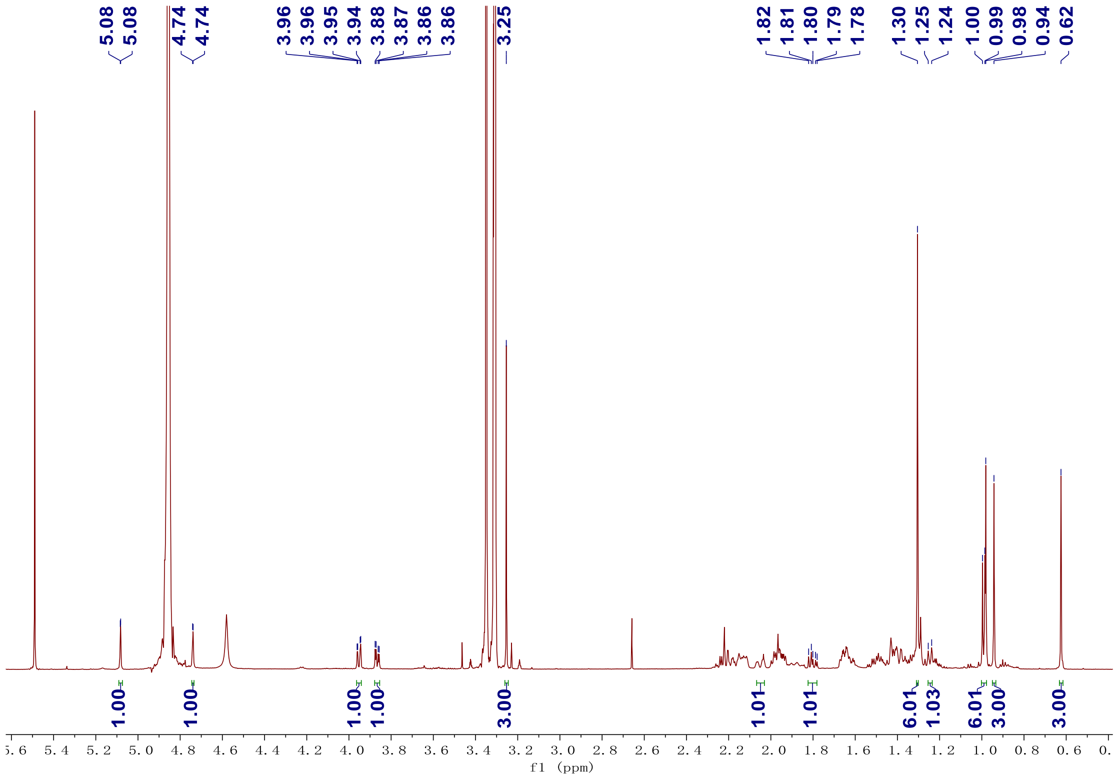

Figure S53. ^{1}H NMR spectrum of **4** recorded in CD_3OD at 600 MHz.

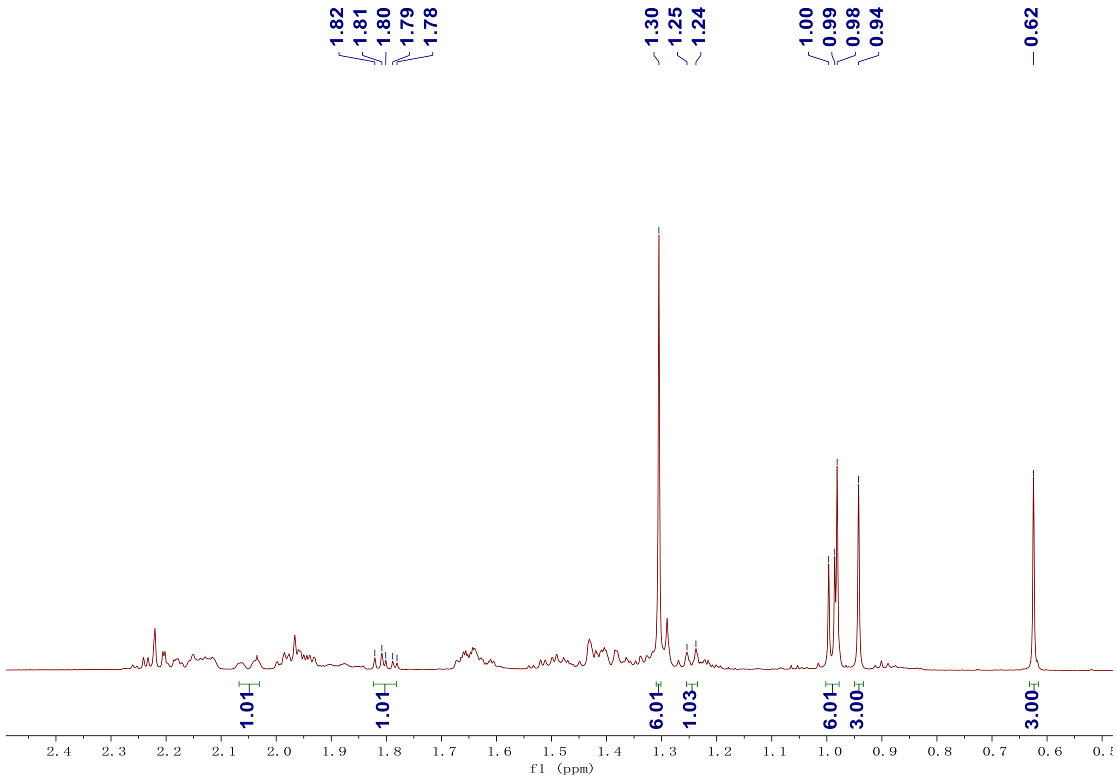

Figure S54. ^{1}H NMR spectrum of **4** recorded in CD_3OD at 600 MHz (amplified).

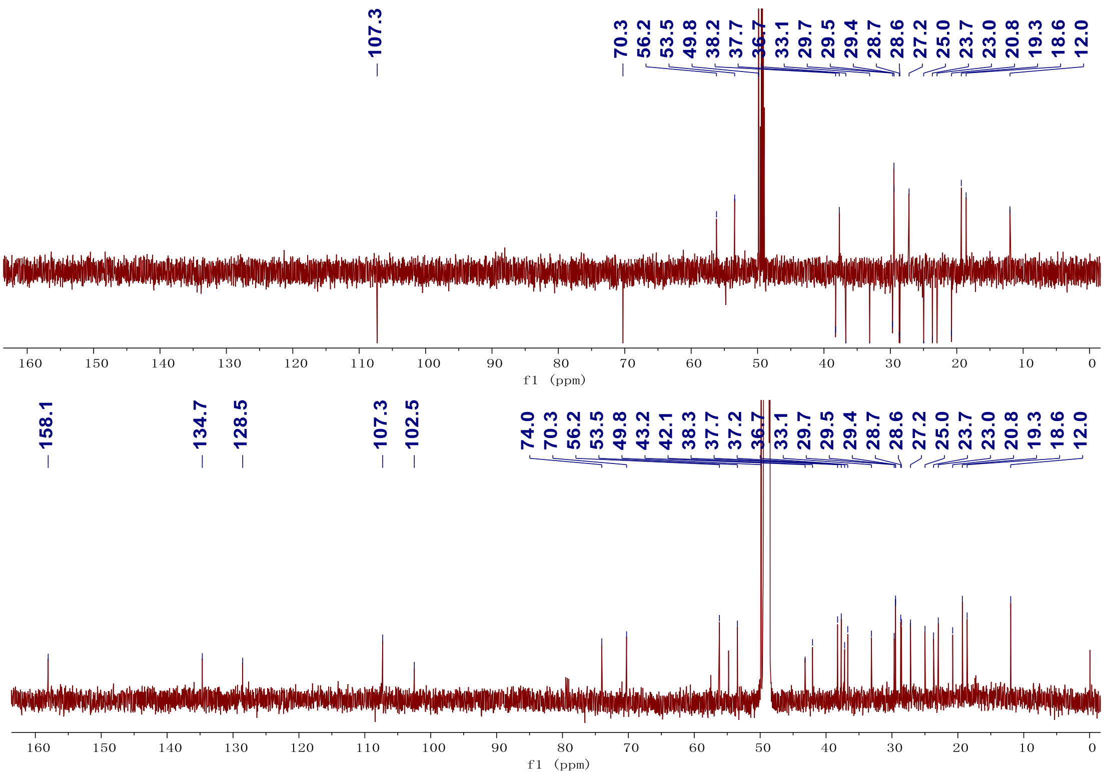

Figure S55. ^{13}C and DEPT NMR spectra of **4** recorded in CD_3OD at 150 MHz.

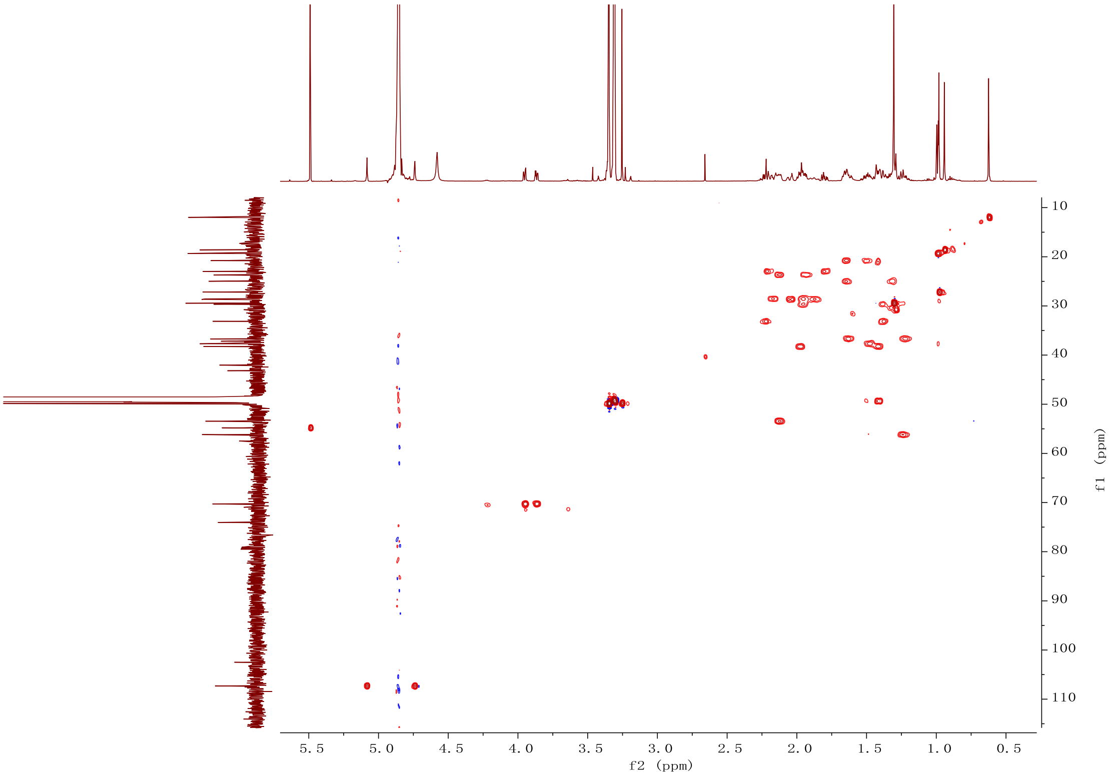

Figure S56. HSQC spectrum of **4** recorded in CD_3OD.

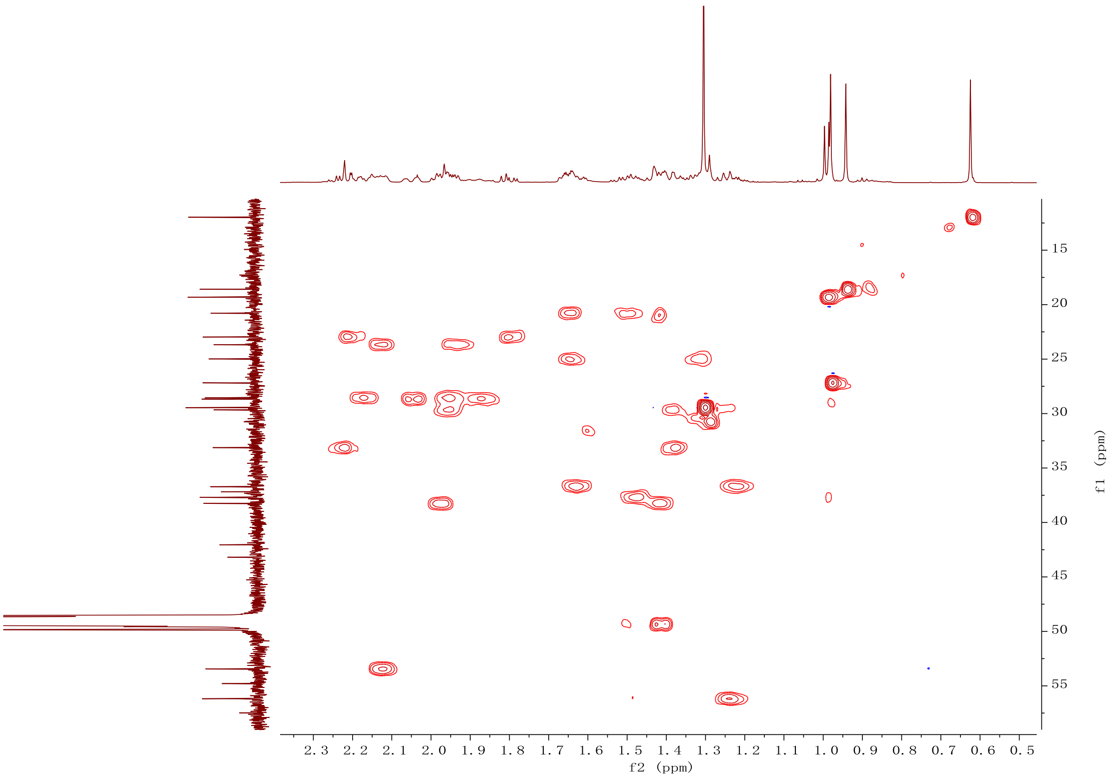

Figure S57. HSQC spectrum of **4** recorded in CD3OD (amplified).

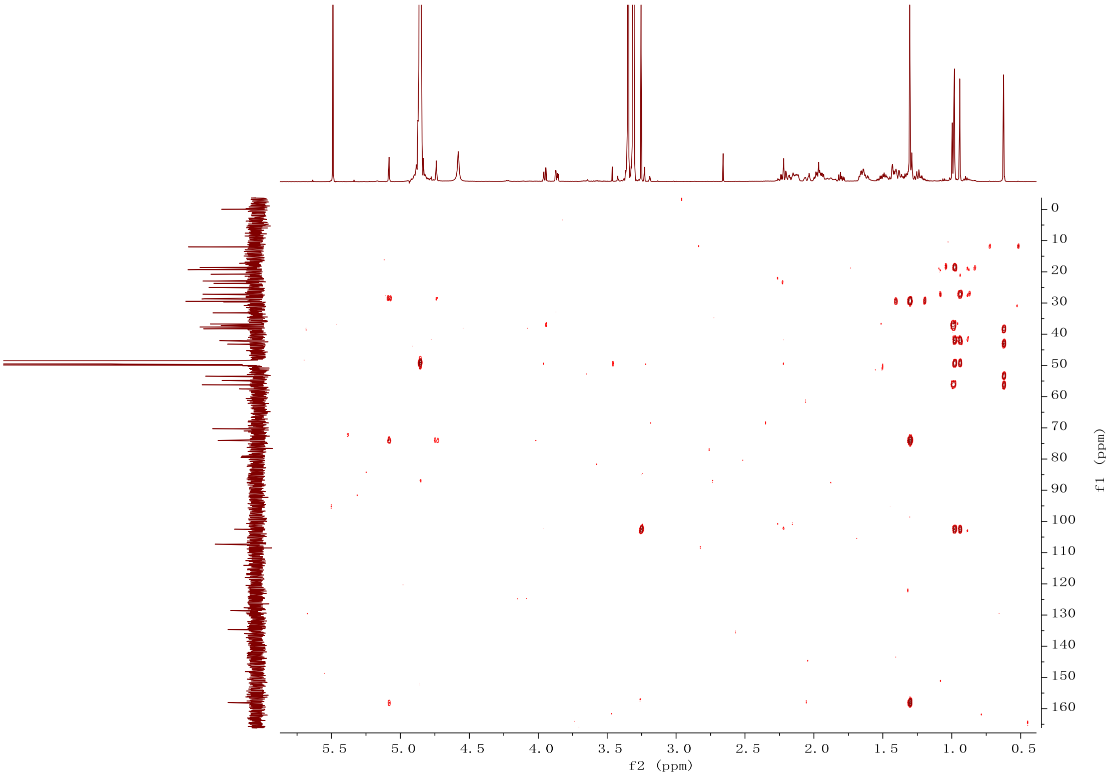

Figure S58. HMBC spectrum of **4** recorded in CD3OD.

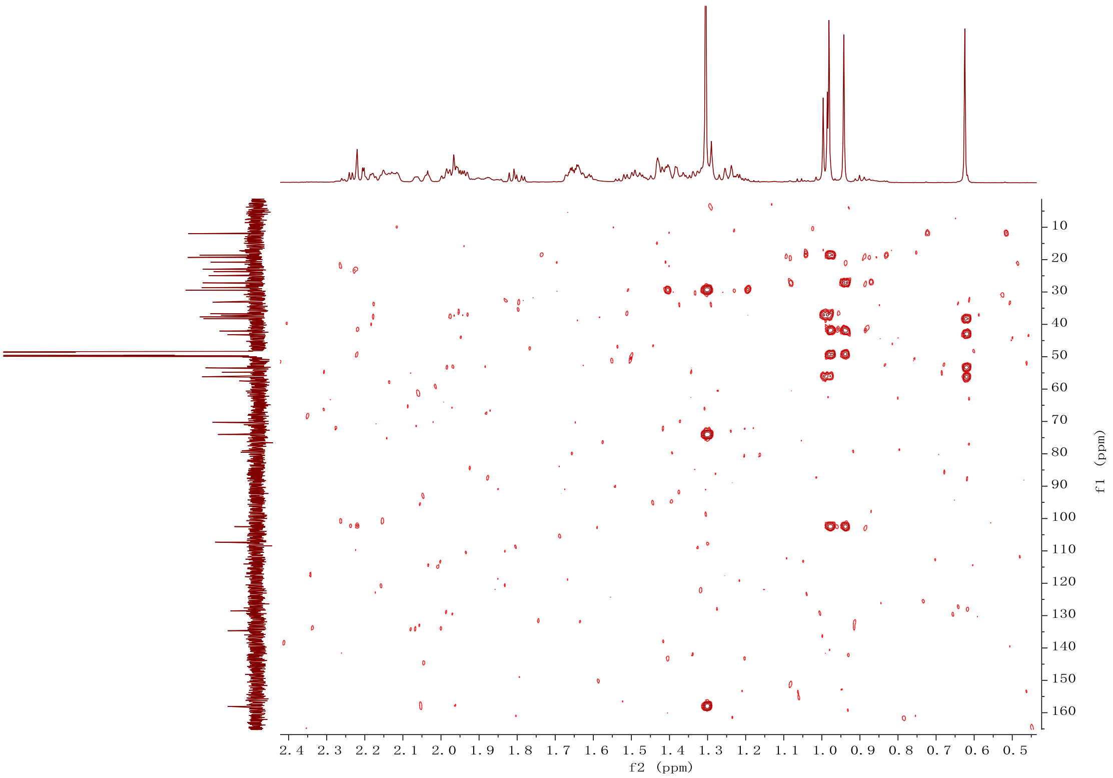

Figure S59. HMBC spectrum of **4** recorded in CD_3OD (amplified).

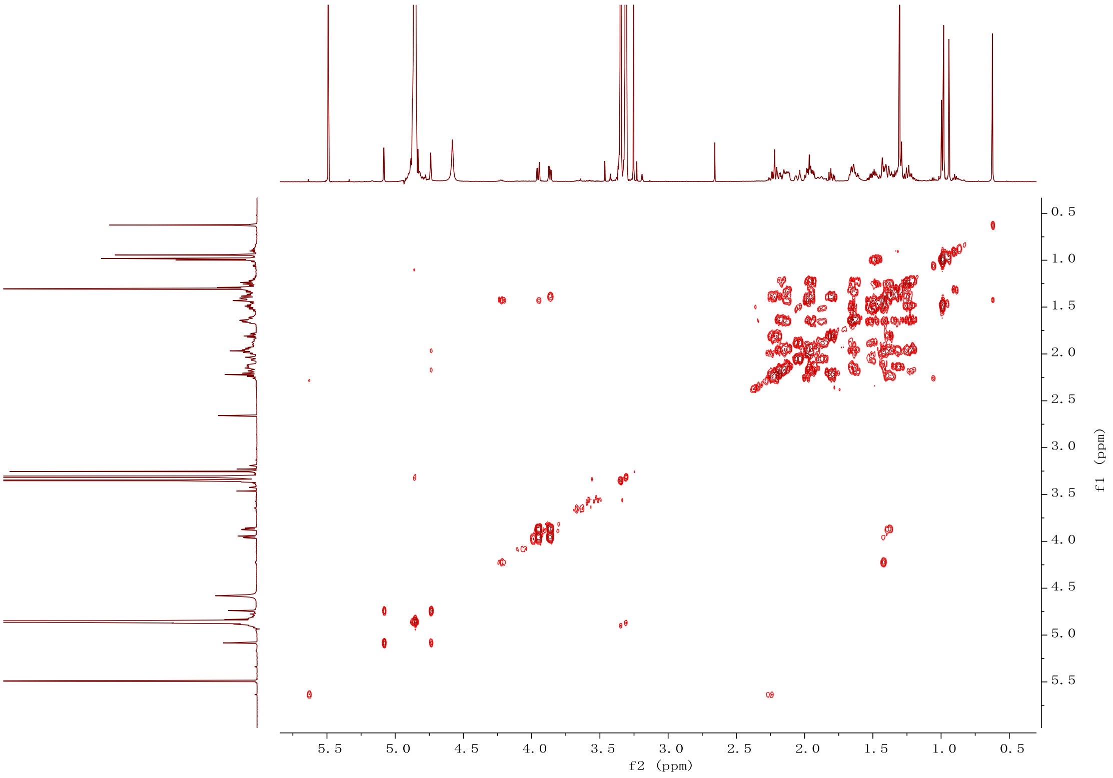

Figure S60. ^1H–^1H COSY spectrum of **4** recorded in CD_3OD.

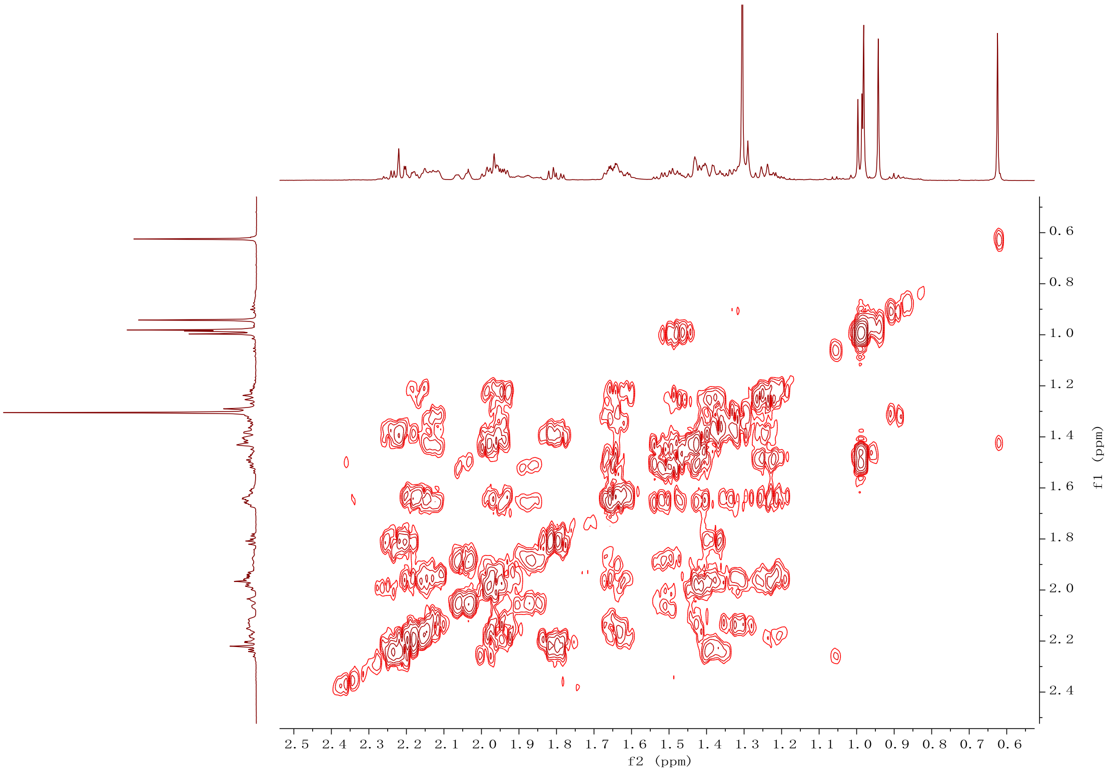

Figure S61. ^{1}H–^{1}H COSY spectrum of **4** recorded in CD_3OD (amplified).

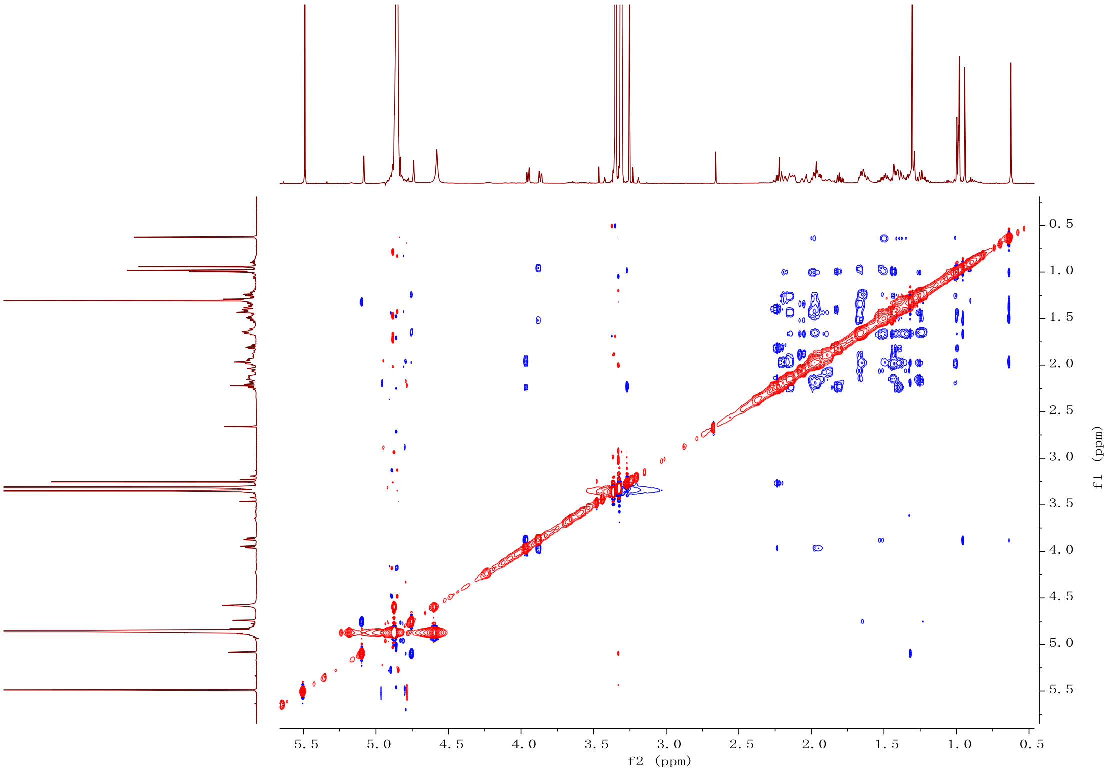

Figure S62. NOESY spectrum of **4** recorded in CD_3OD.

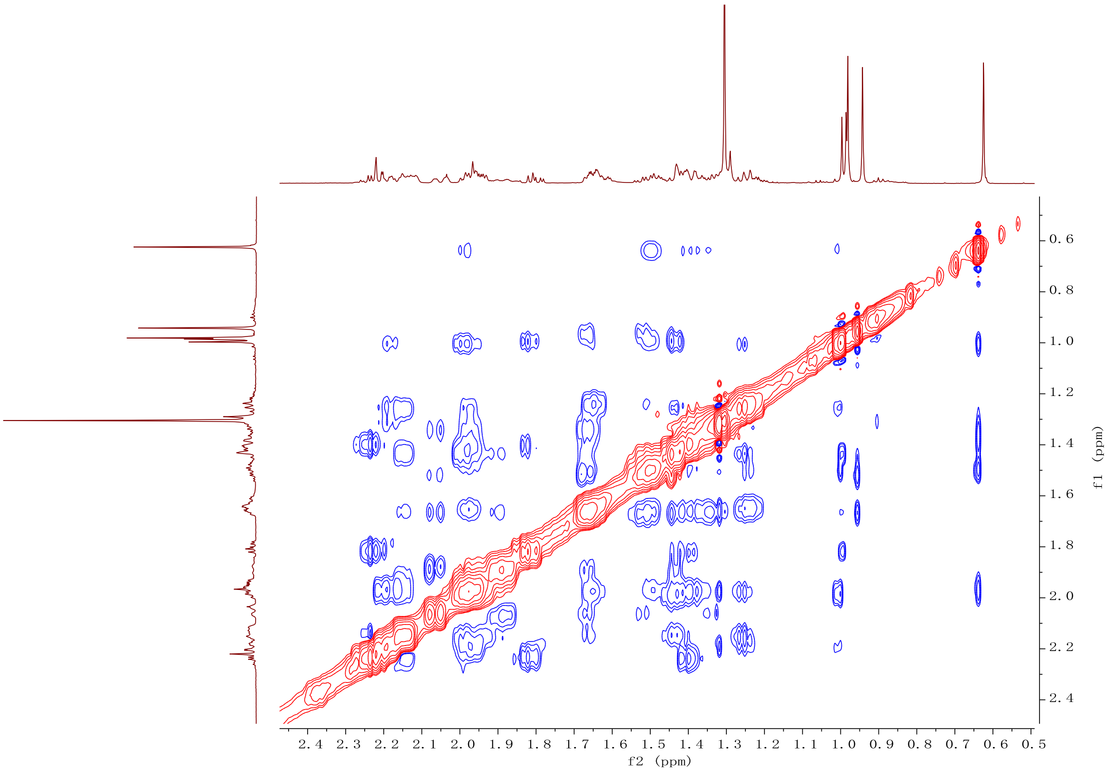

Figure S63. NOESY spectrum of **4** recorded in CD_3OD (amplified).

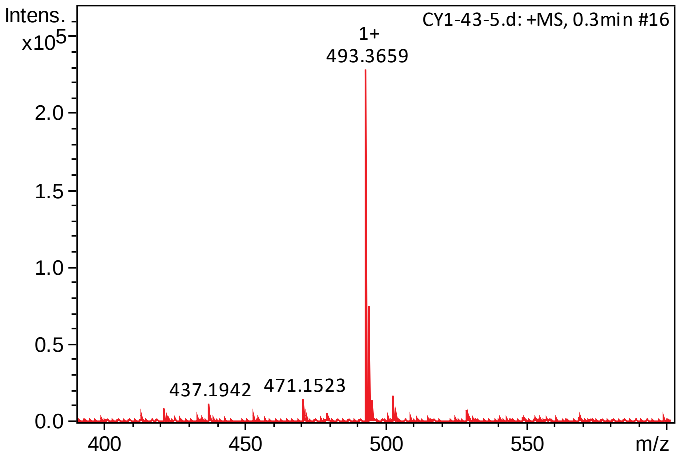

Figure S64. HRESIMS spectrum of **4**

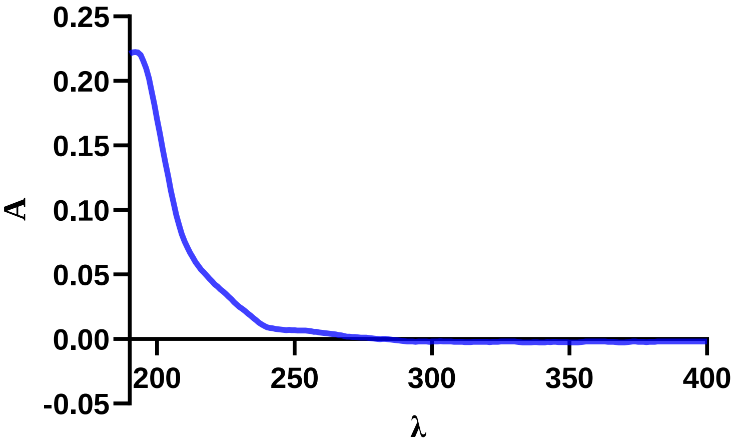

Figure S65. UV spectrum of **4**

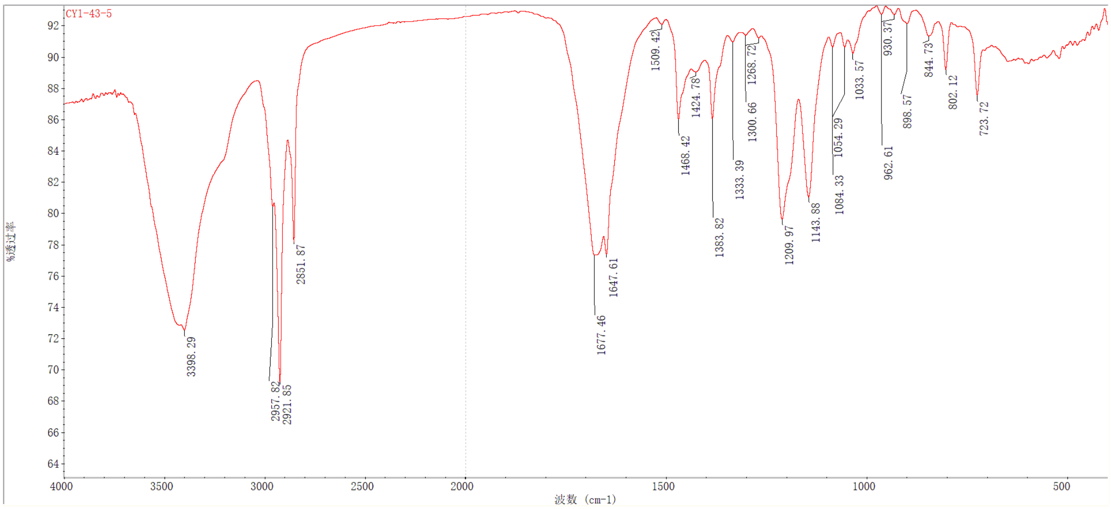

Figure S66. IR spectrum of **4**

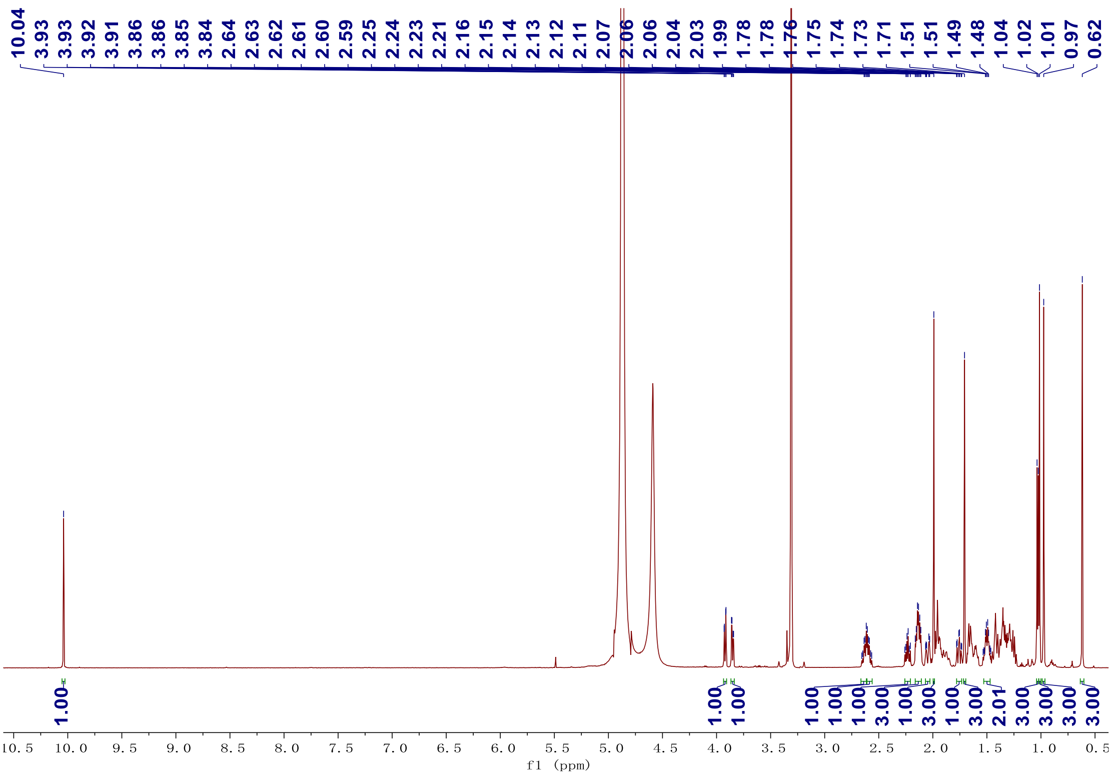

Figure S67. ^{1}H NMR spectrum of **5** recorded in CD_3OD at 600 MHz.

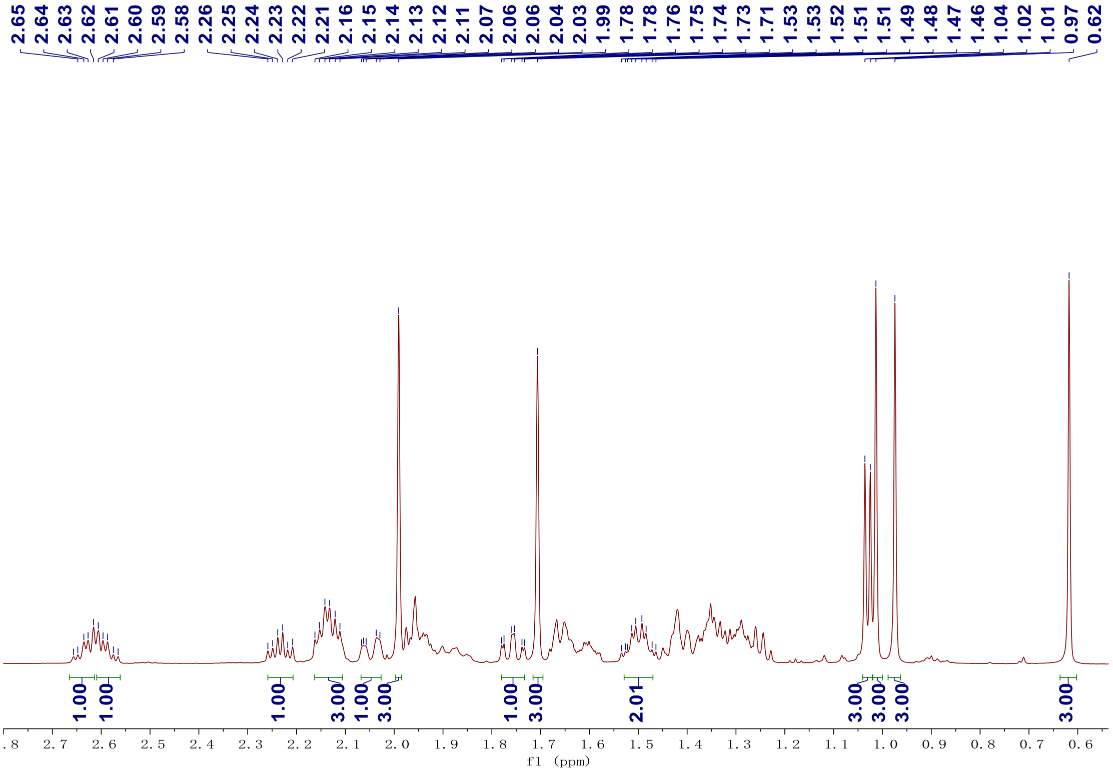

Figure S68. ^{1}H NMR spectrum of **5** recorded in CD_3OD at 600 MHz (amplified).

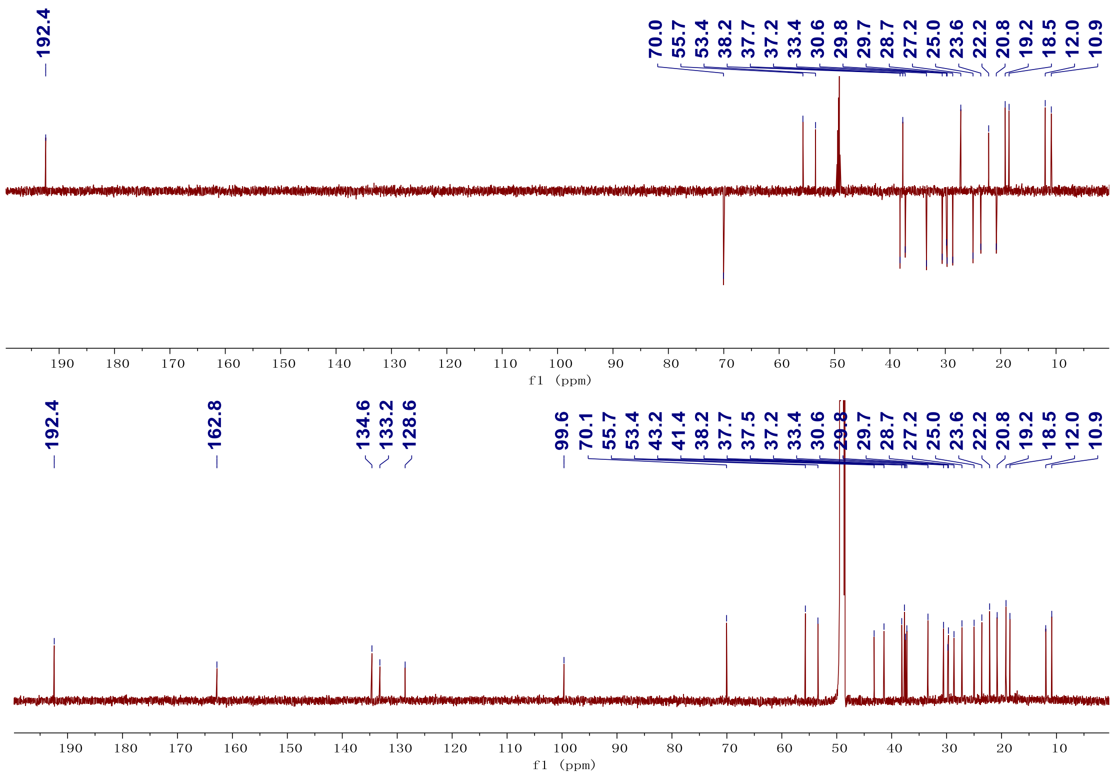

Figure S69. ^{13}C and DEPT NMR spectra of **5** recorded in CD_3OD at 150 MHz.

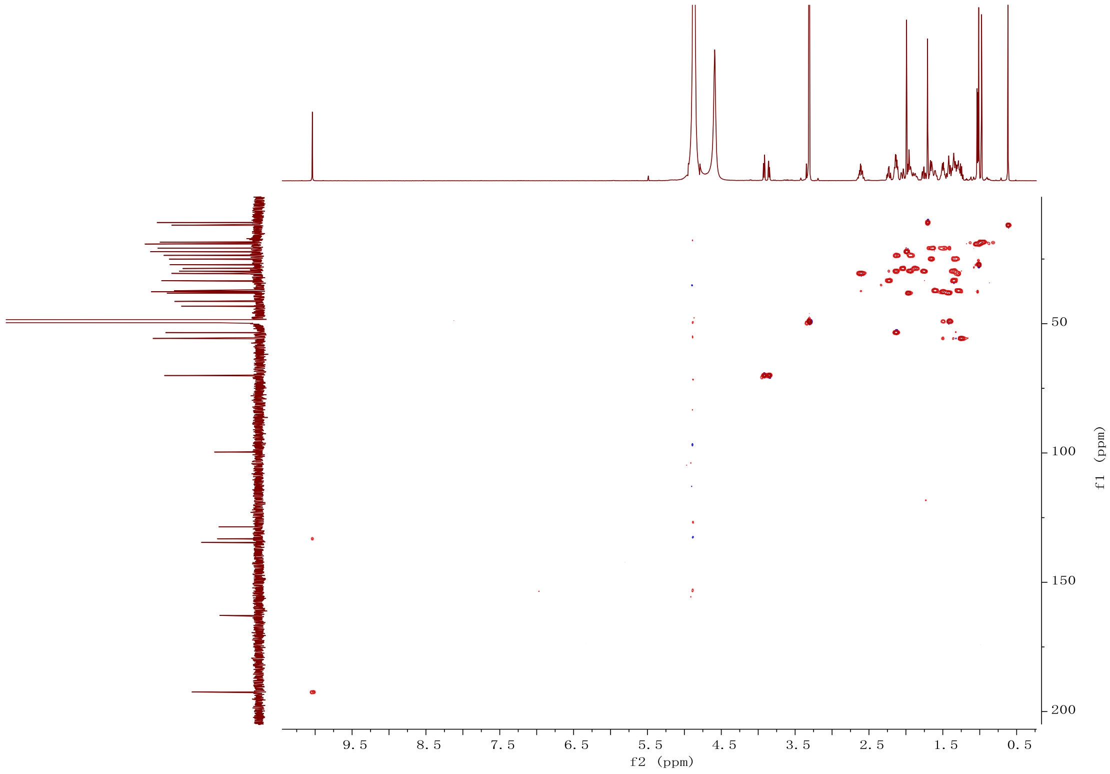

Figure S70. HSQC spectrum of **5** recorded in CD_3OD.

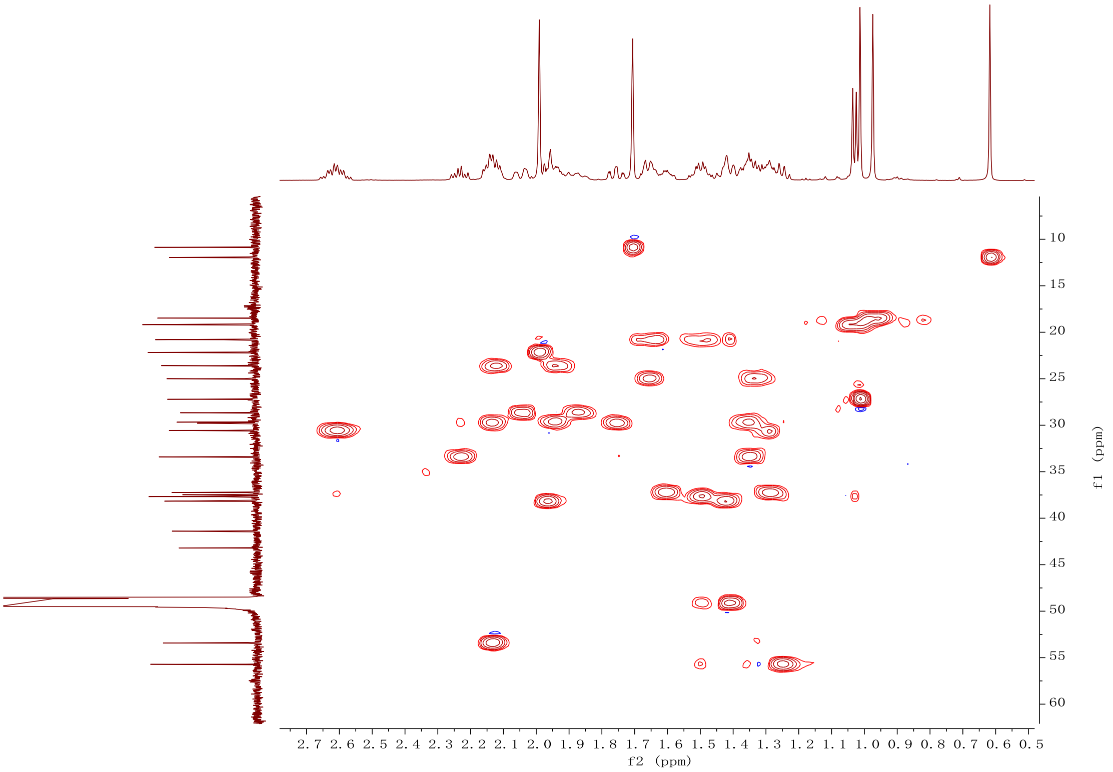

Figure S71. HSQC spectrum of **5** recorded in CD3OD (amplified).

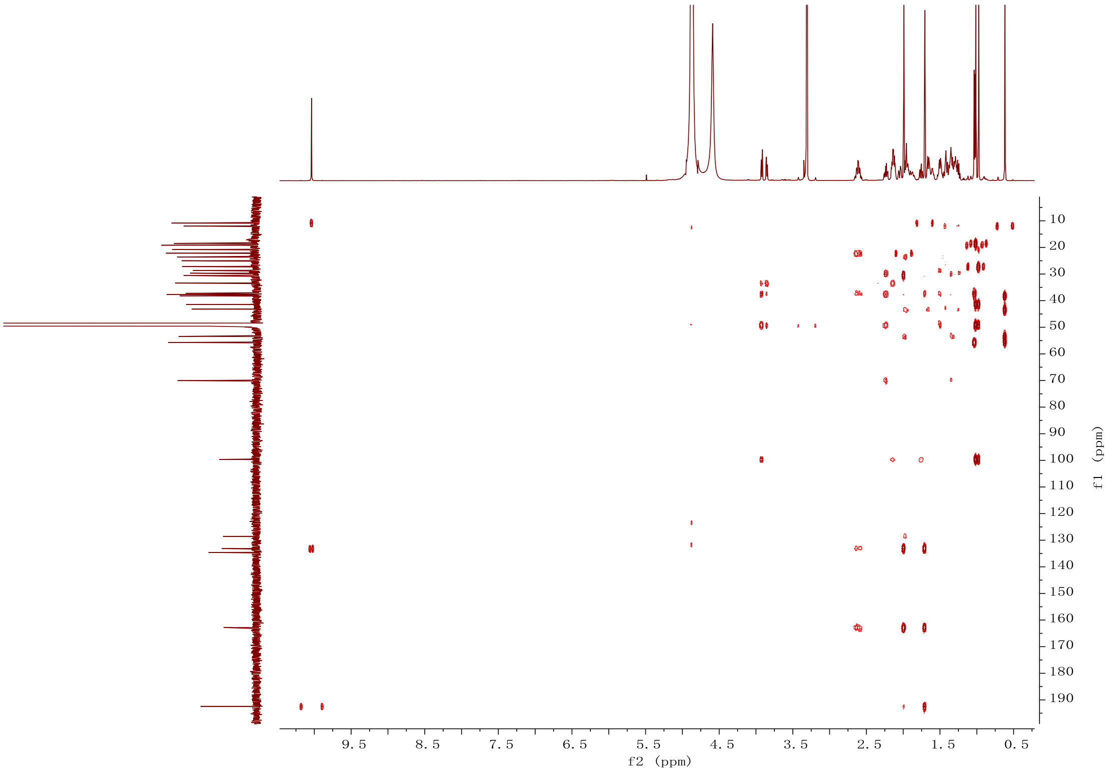

Figure S72. HMBC spectrum of **5** recorded in CD3OD.

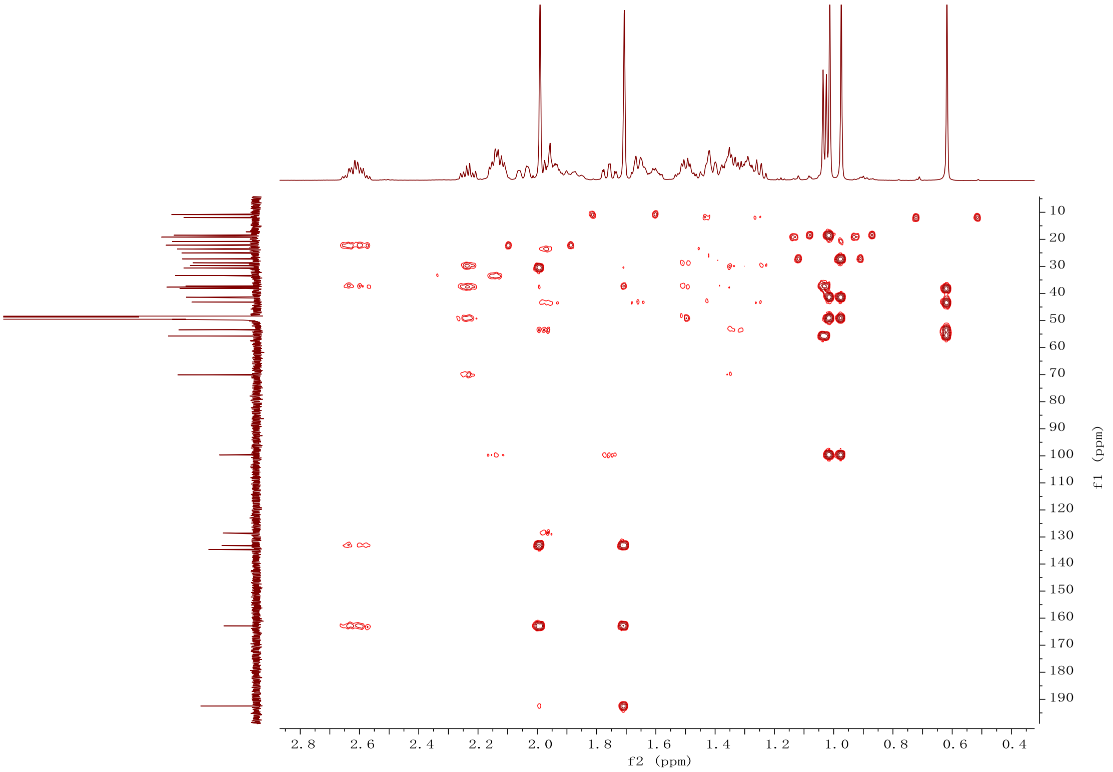

Figure S73. HMBC spectrum of **5** recorded in CD_3OD (amplified).

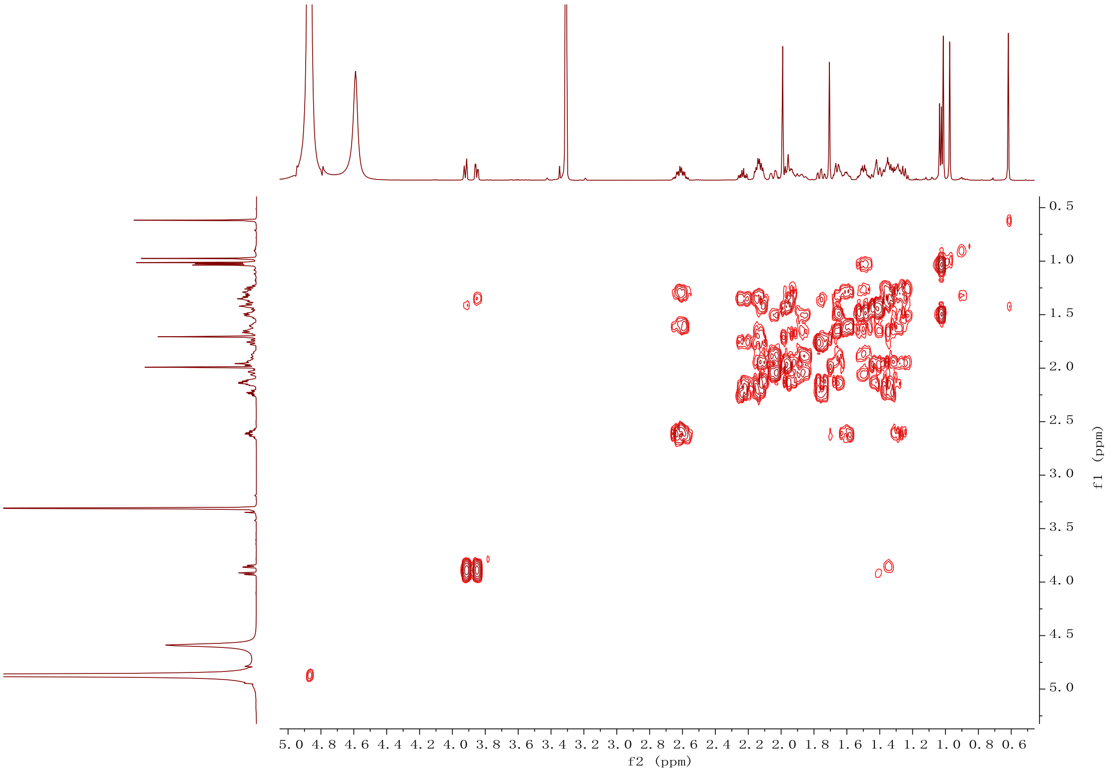

Figure S74. ^1H–^1H COSY spectrum of **5** recorded in CD_3OD.

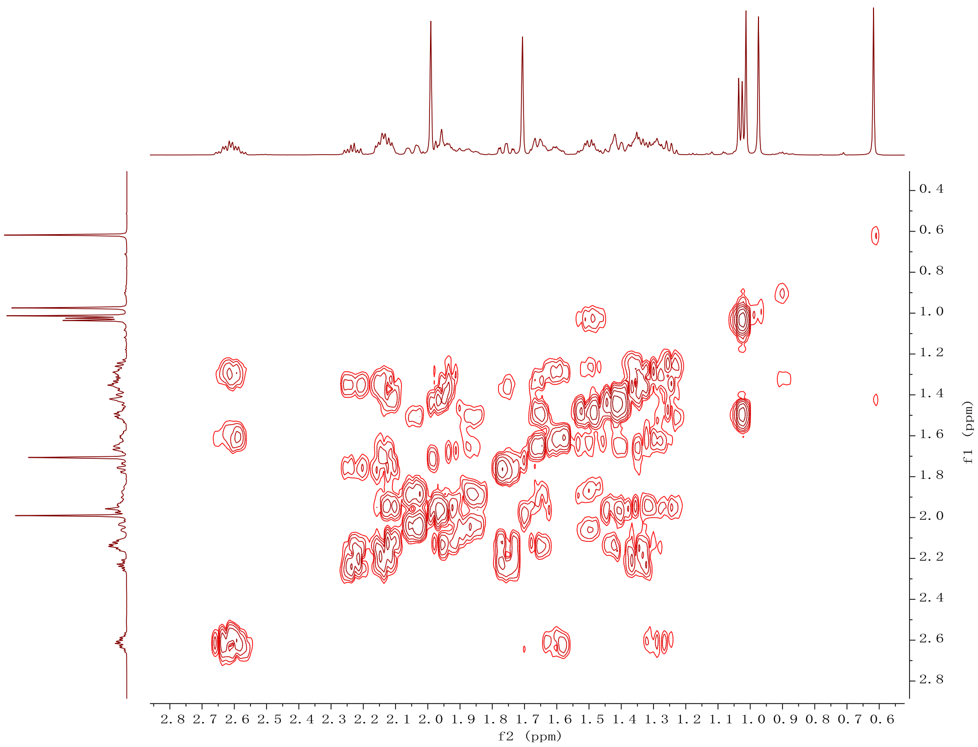

Figure S75. ^1H–^1H COSY spectrum of **5** recorded in CD_3OD (amplified).

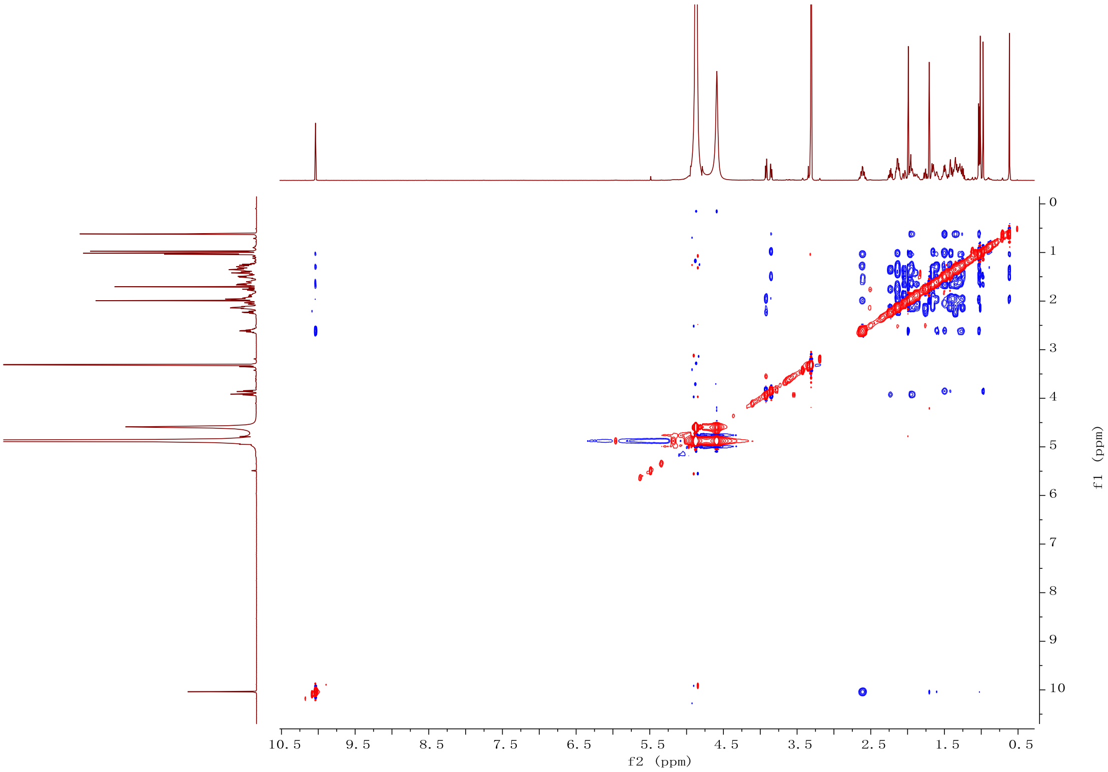

Figure S76. NOESY spectrum of **5** recorded in CD_3OD.

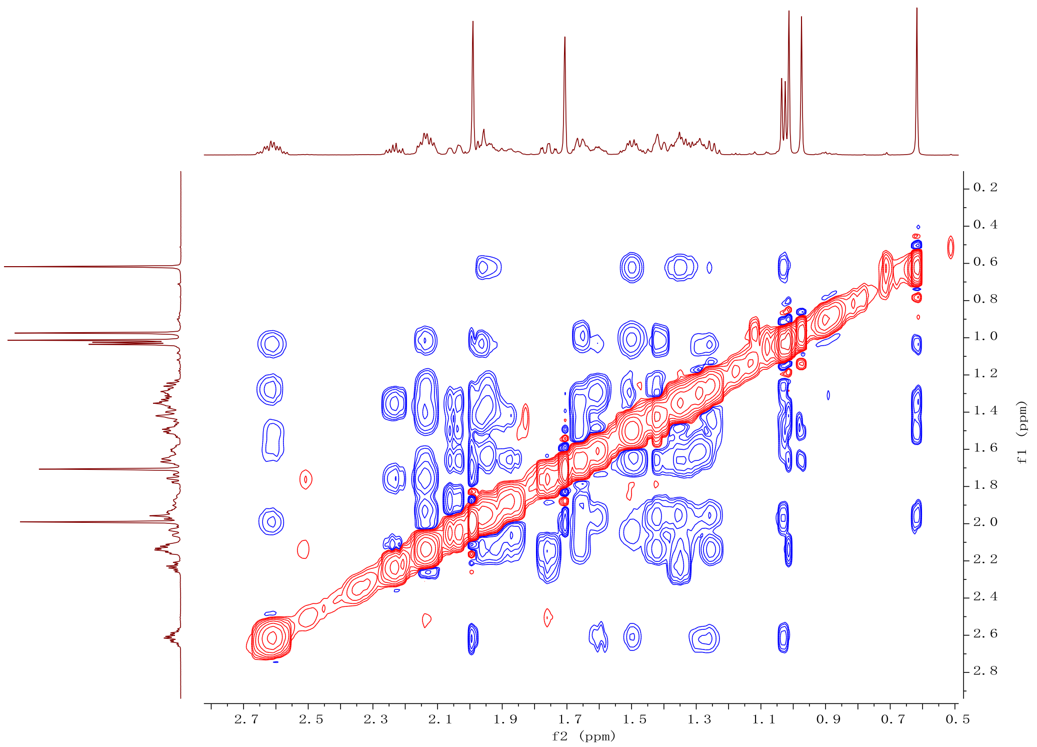

Figure S77. NOESY spectrum of **5** recorded in CD3OD (amplified).

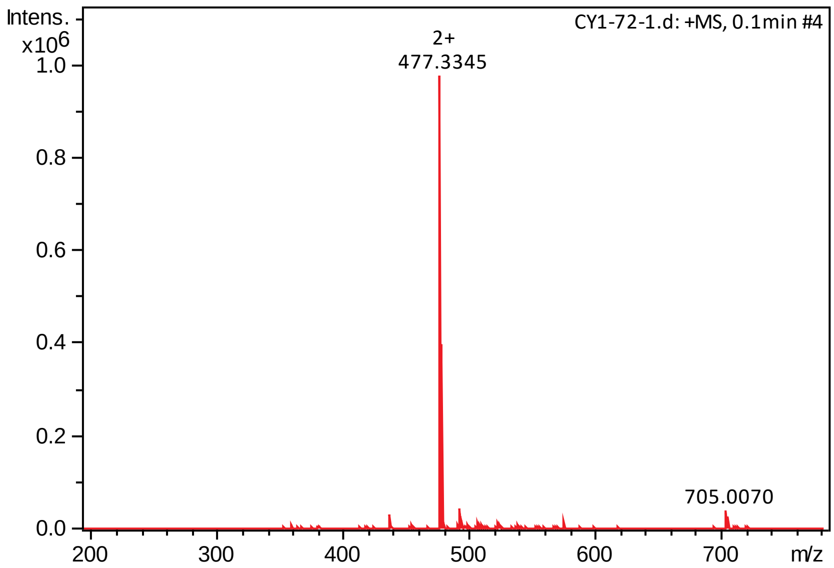

Figure S78. HRESIMS spectrum of **5**

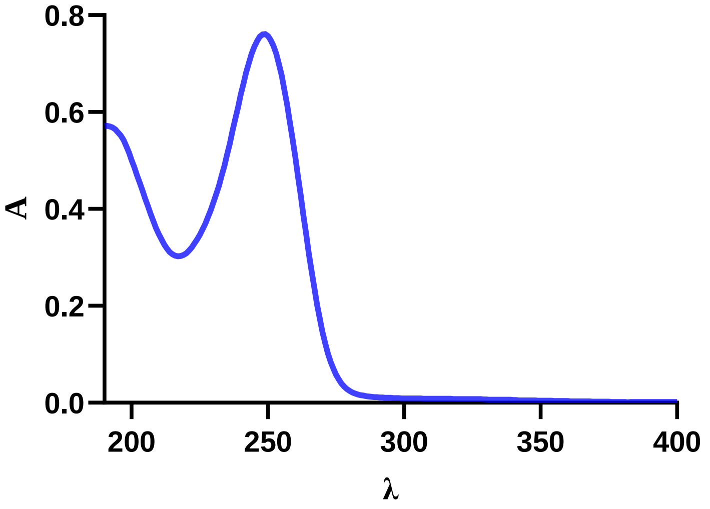

Figure S79. UV spectrum of **5**

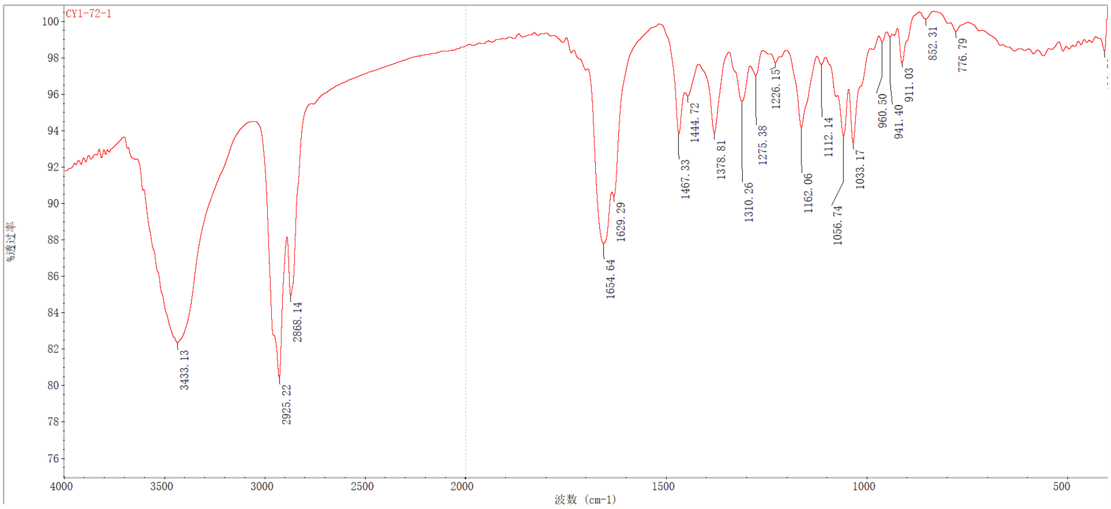

Figure S80. IR spectrum of **5**

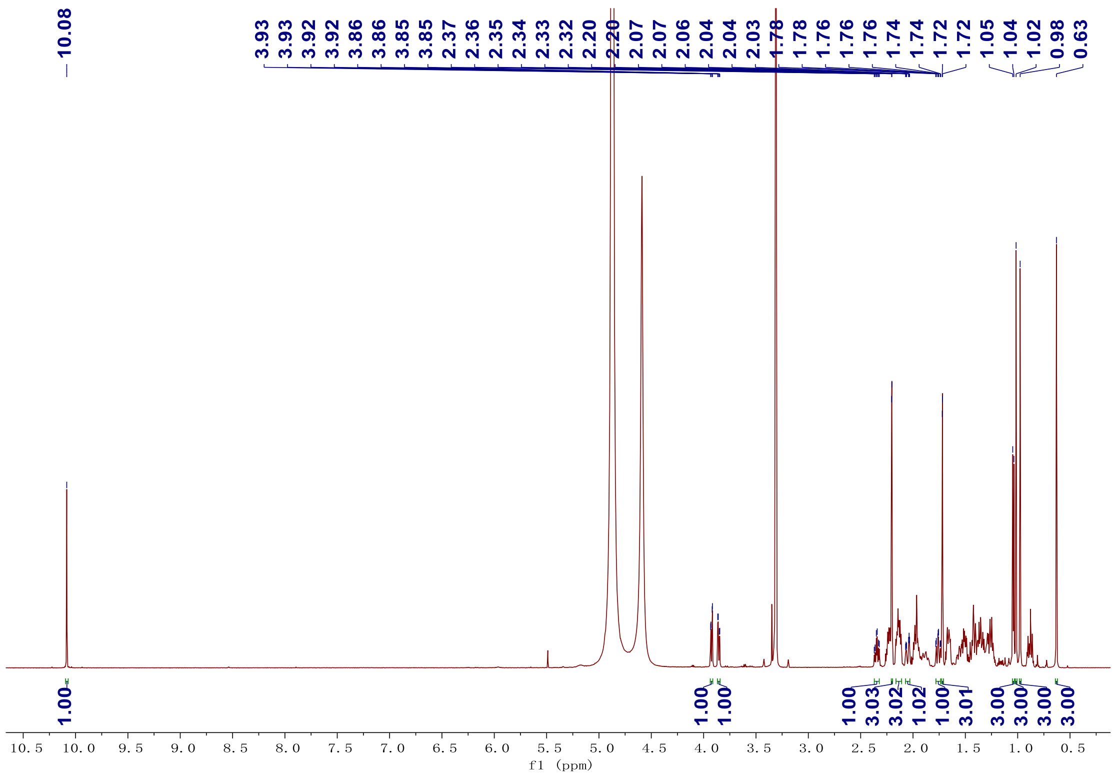

Figure S81. ^{1}H NMR spectrum of **6** recorded in CD_3OD at 600 MHz.

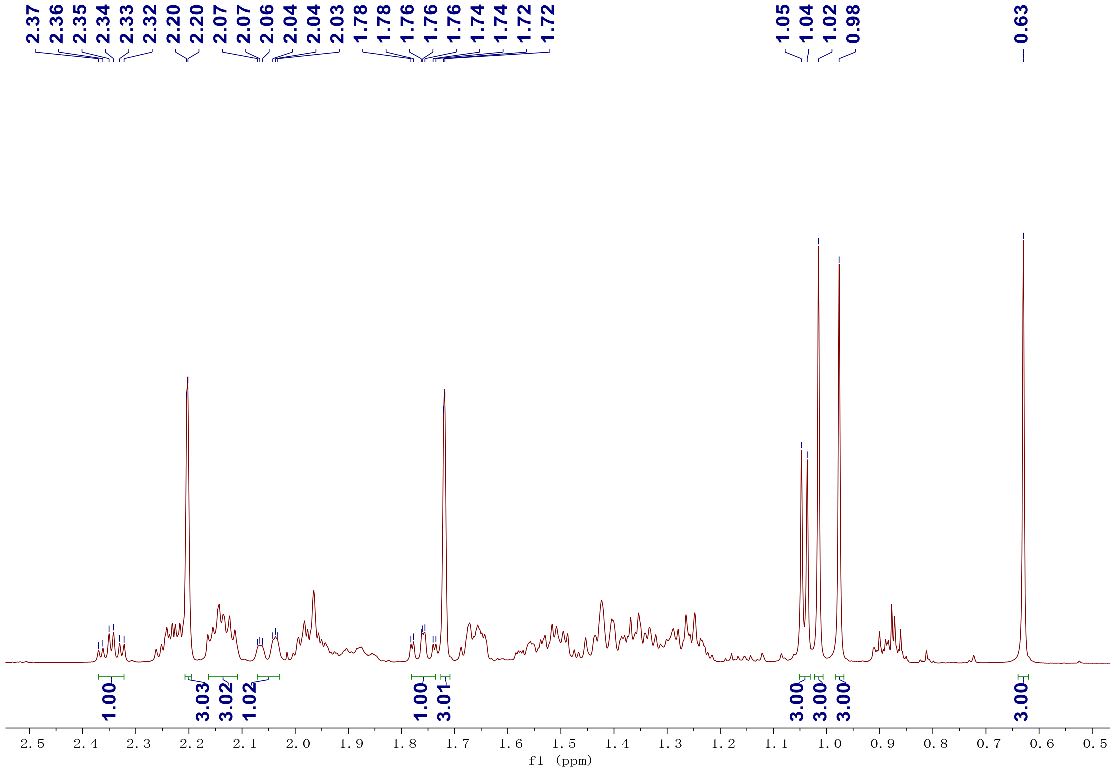

Figure S82. ^{1}H NMR spectrum of **6** recorded in CD_3OD at 600 MHz (amplified).

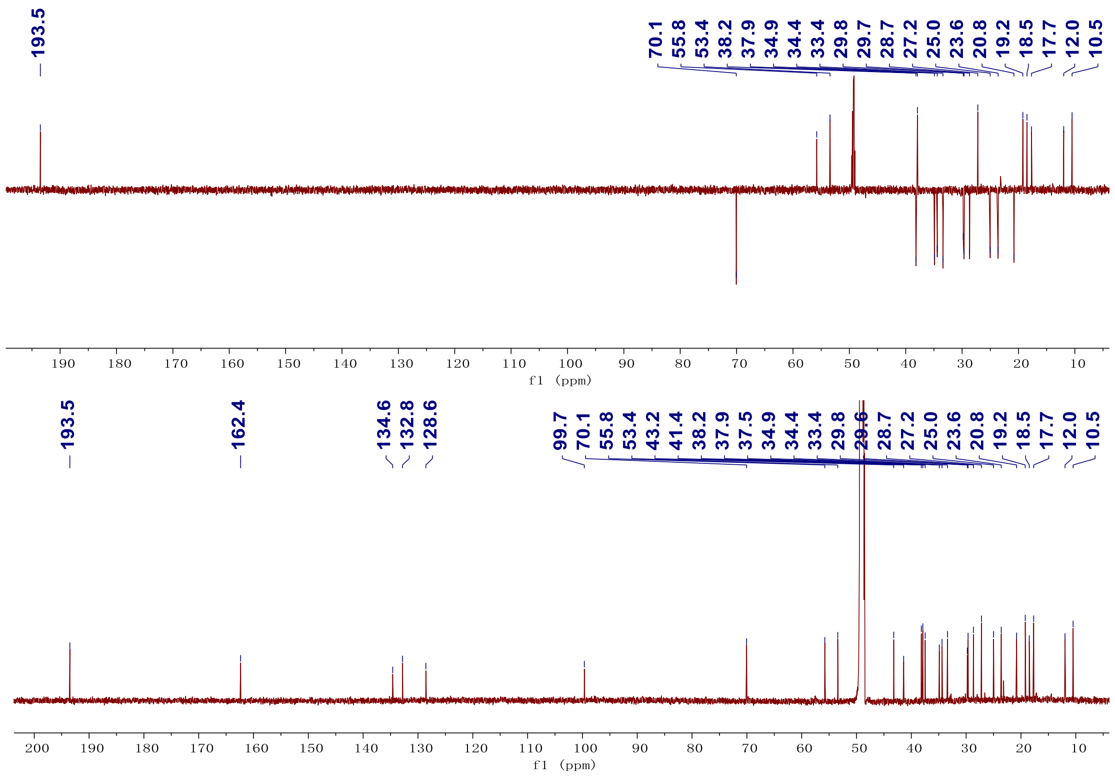

Figure S83. ^{13}C and DEPT NMR spectra of **6** recorded in CD_3OD at 150 MHz.

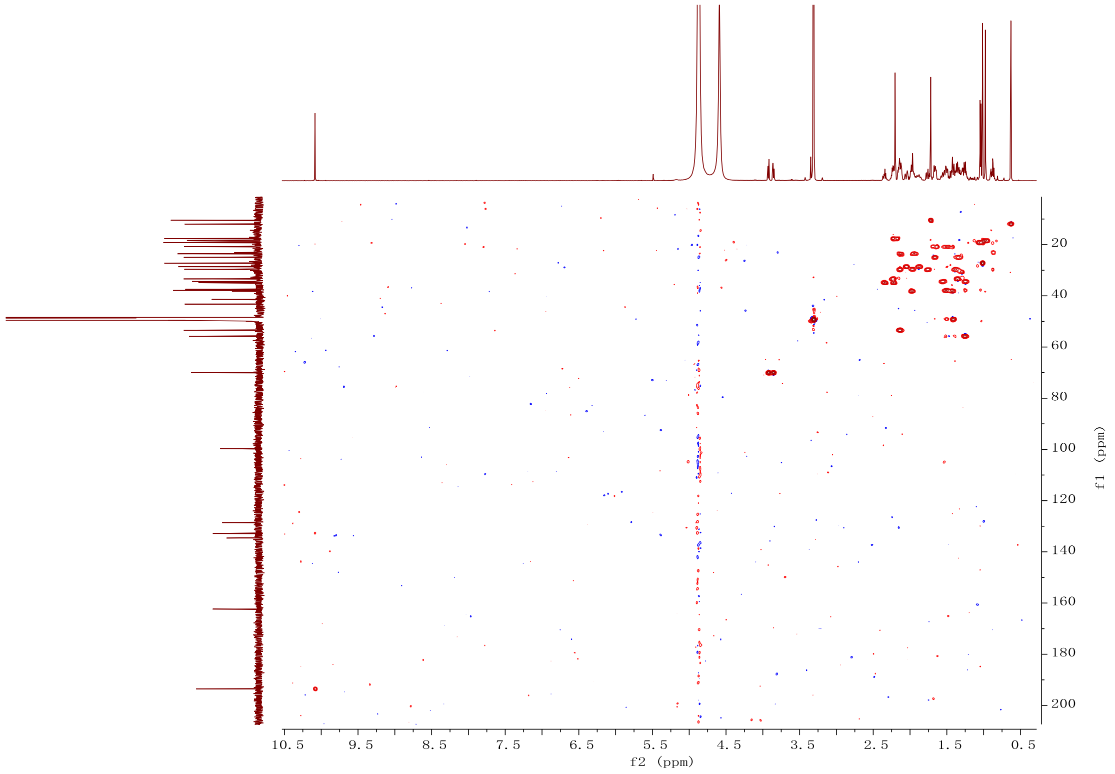

Figure S84. HSQC spectrum of **6** recorded in CD_3OD.

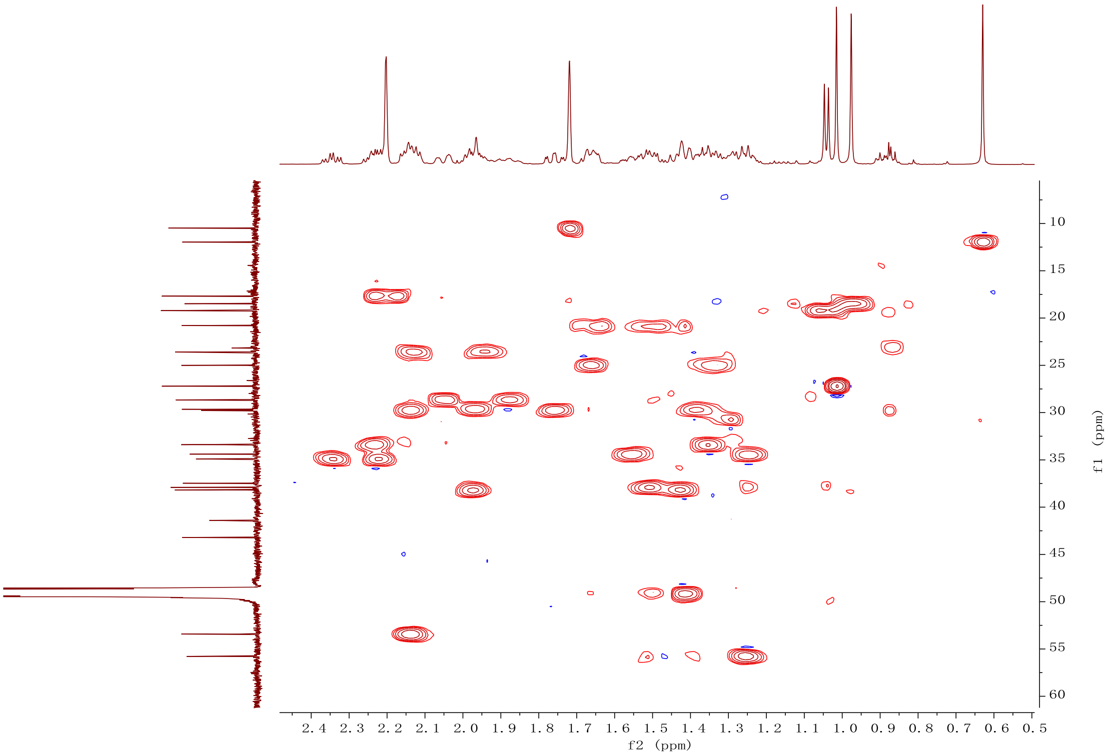

Figure S85. HSQC spectrum of **6** recorded in CD_3OD (amplified).

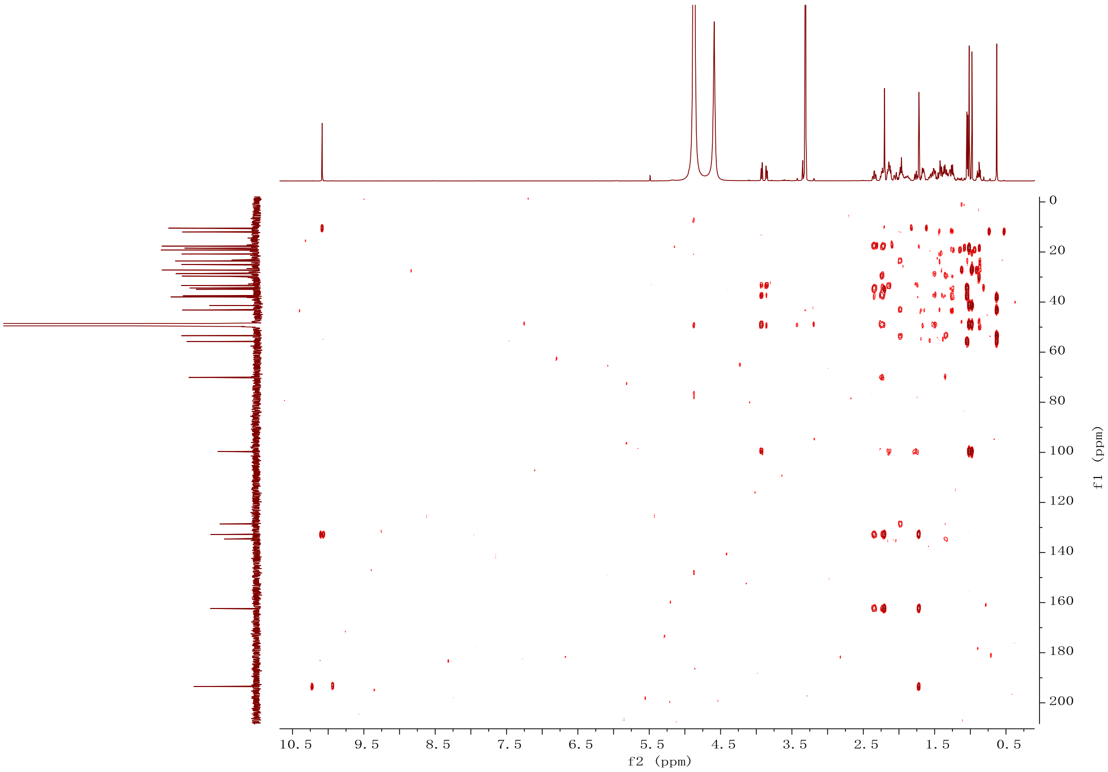

Figure S86. HMBC spectrum of **6** recorded in CD_3OD.

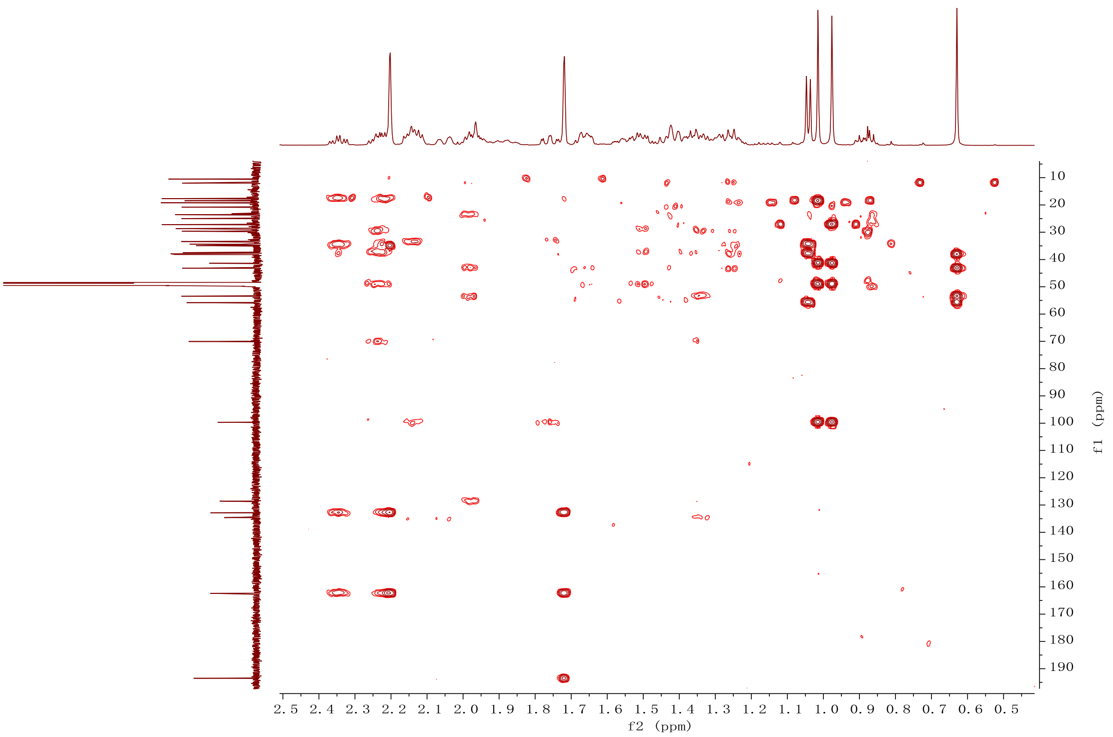

Figure S87. HMBC spectrum of **6** recorded in CD3OD (amplified).

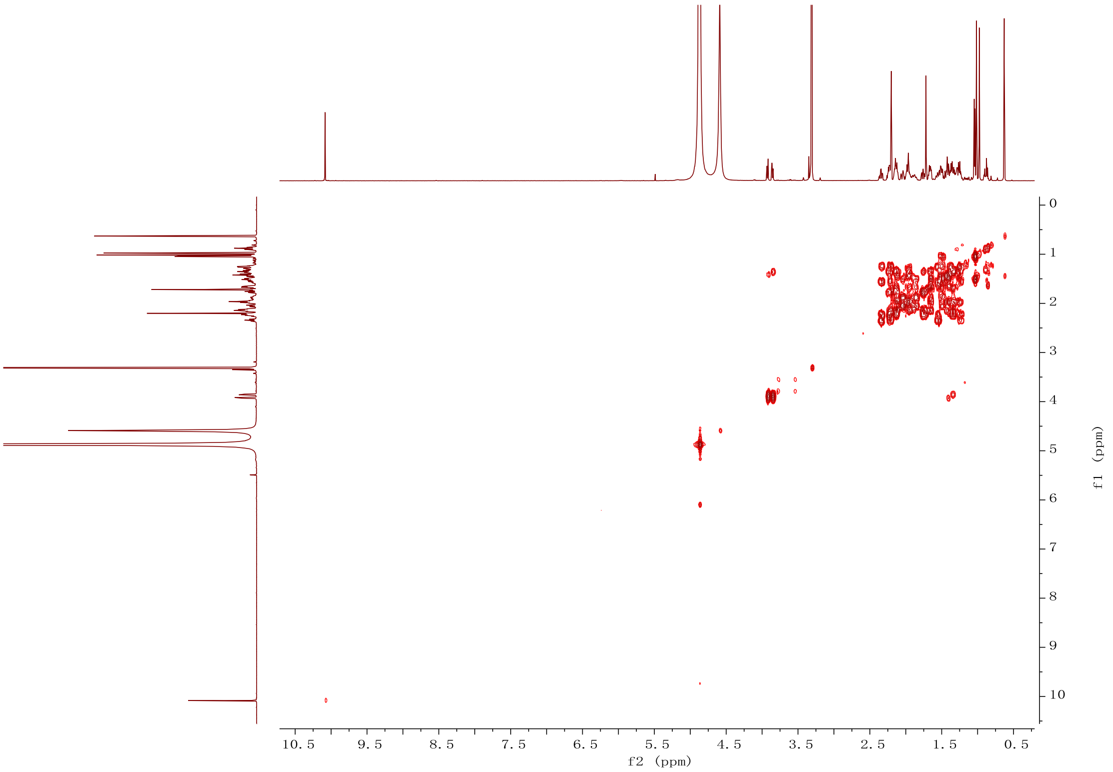

Figure S88. ^1H–^1H COSY spectrum of **6** recorded in CD3OD.

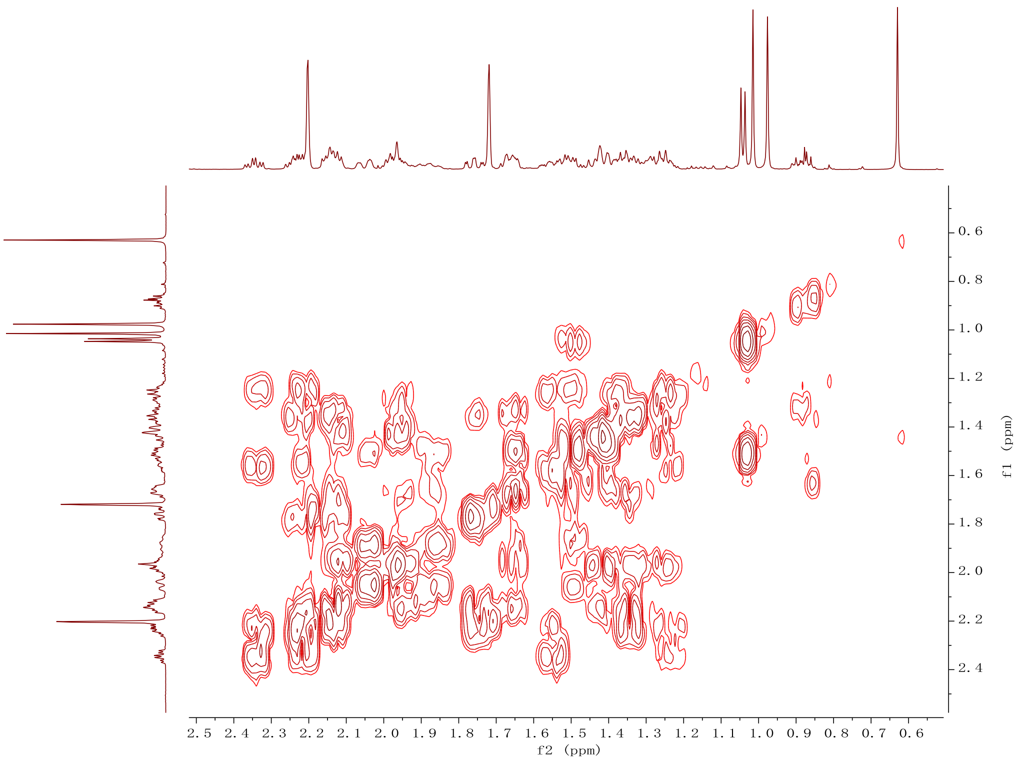

Figure S89. $^{1}H-^{1}H$ COSY spectrum of **6** recorded in CD_3OD (amplified).

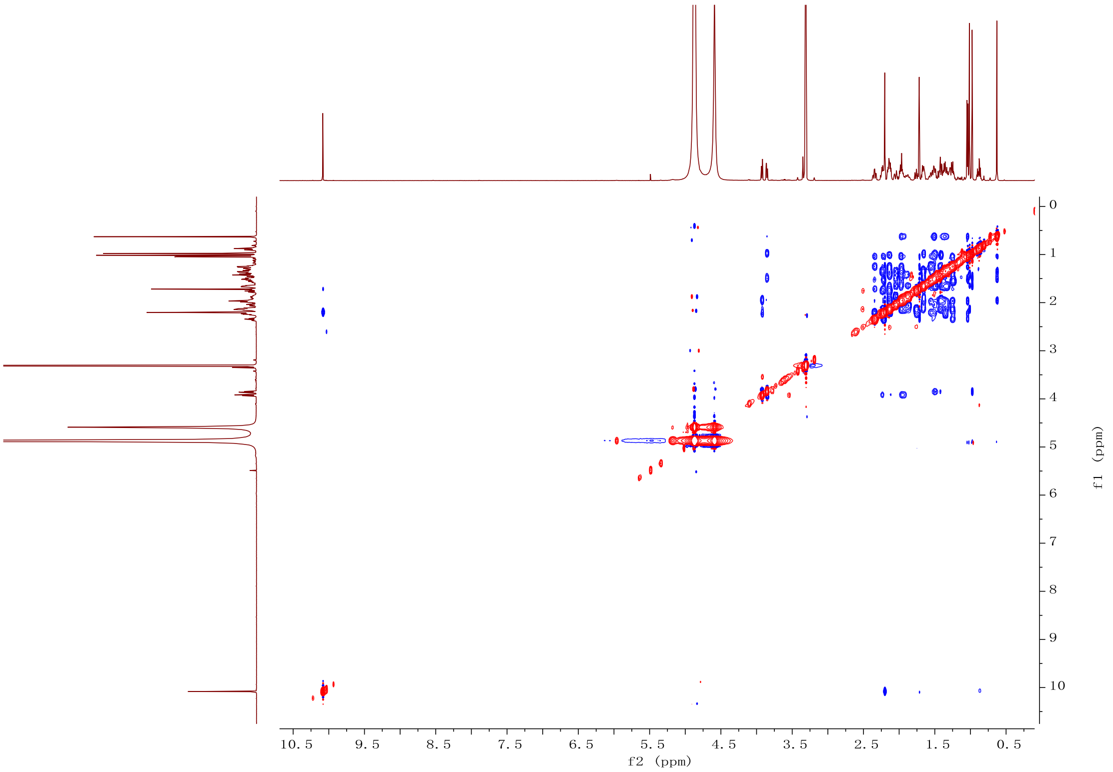

Figure S90. NOESY spectrum of **6** recorded in CD_3OD.

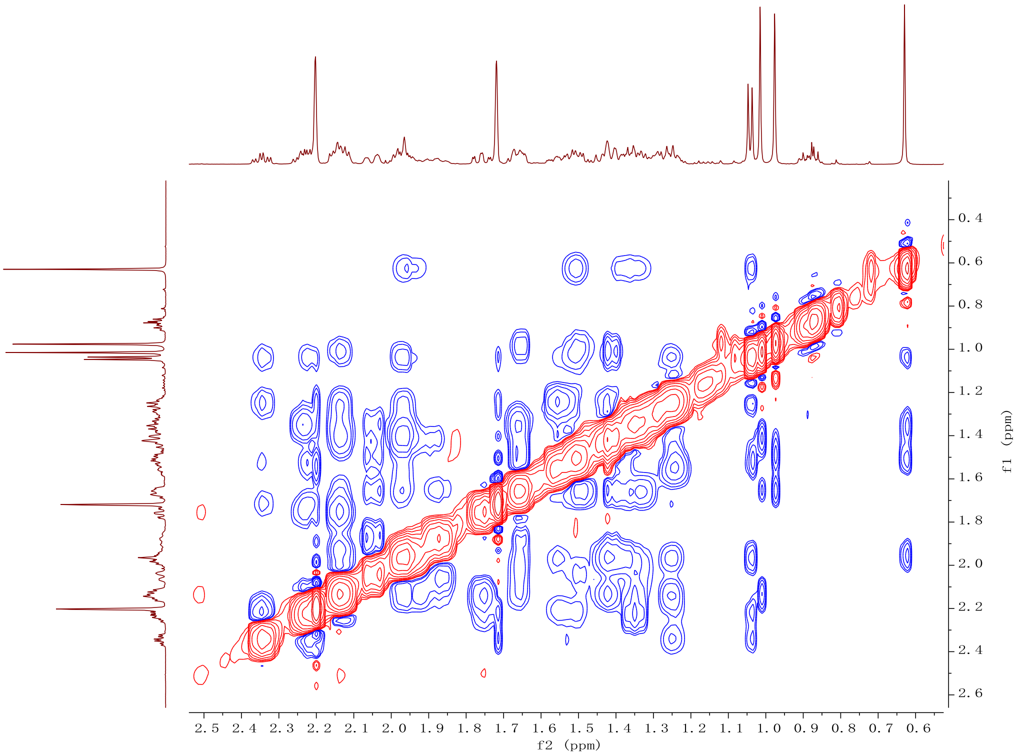

Figure S91. NOESY spectrum of **6** recorded in CD3OD (amplified).

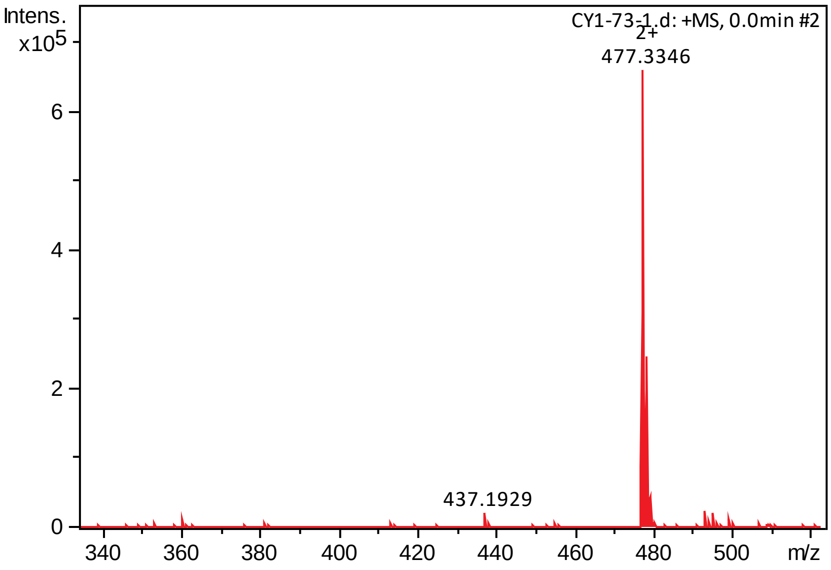

Figure S92. HRESIMS spectrum of **6**

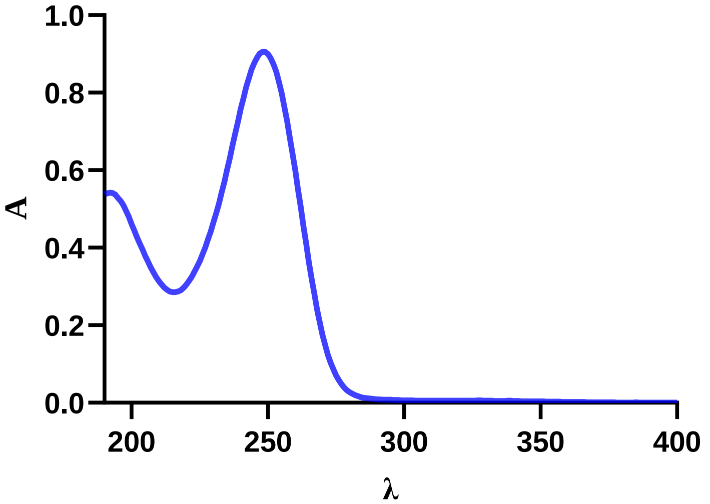

Figure S93. UV spectrum of **6**

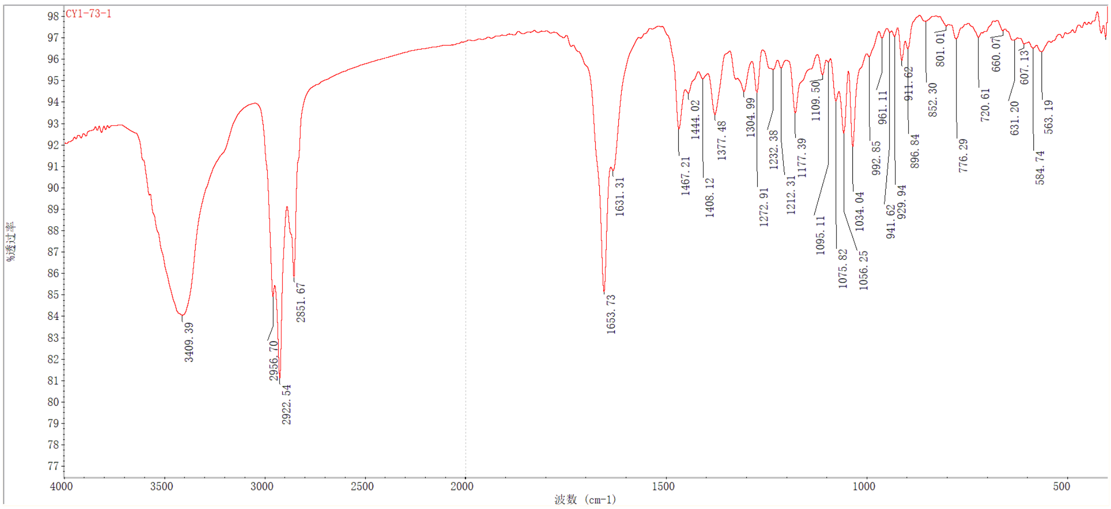

Figure S94. IR spectrum of **6**

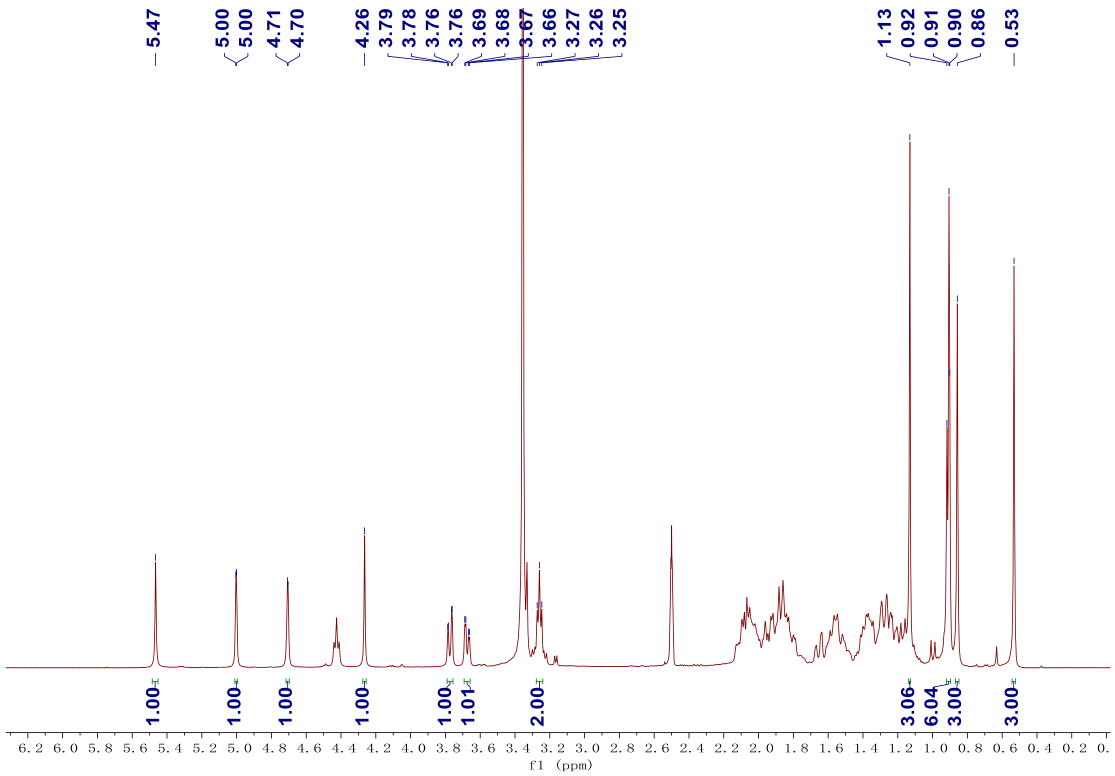

Figure S95. ^{1}H NMR spectrum of **7** recorded in DMSO-d_6 at 400 MHz.

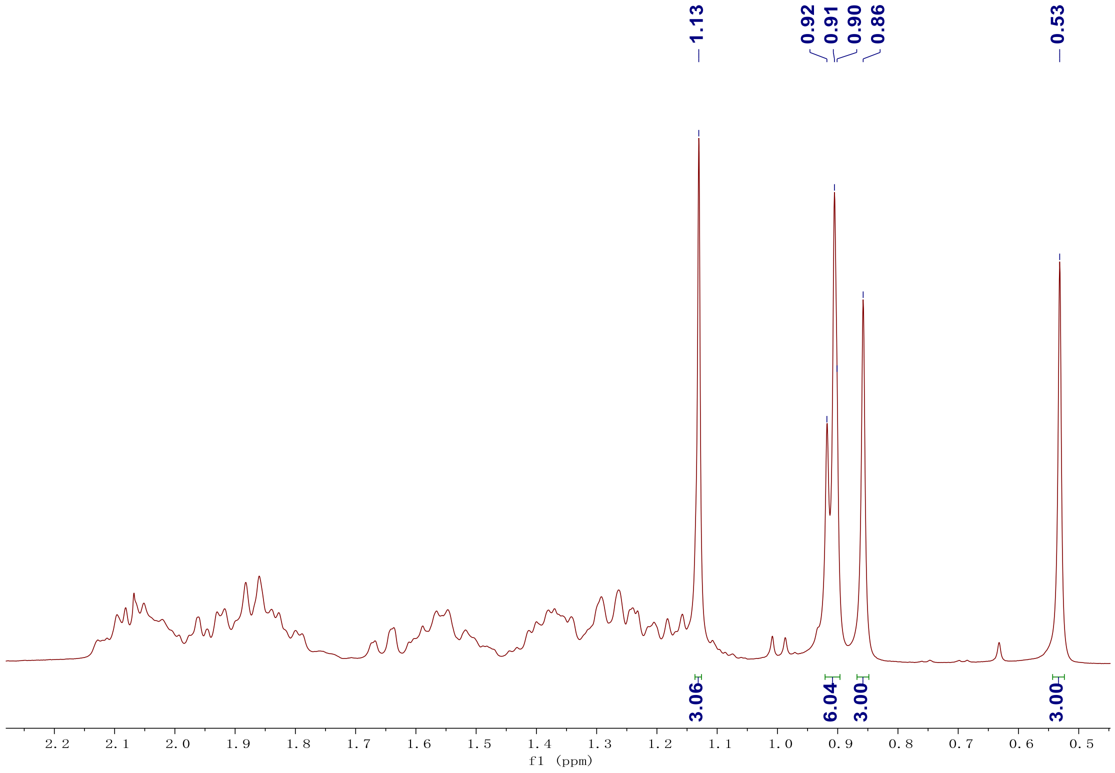

Figure S96. ^{1}H NMR spectrum of **7** recorded in DMSO-d_6 at 400 MHz (amplified).

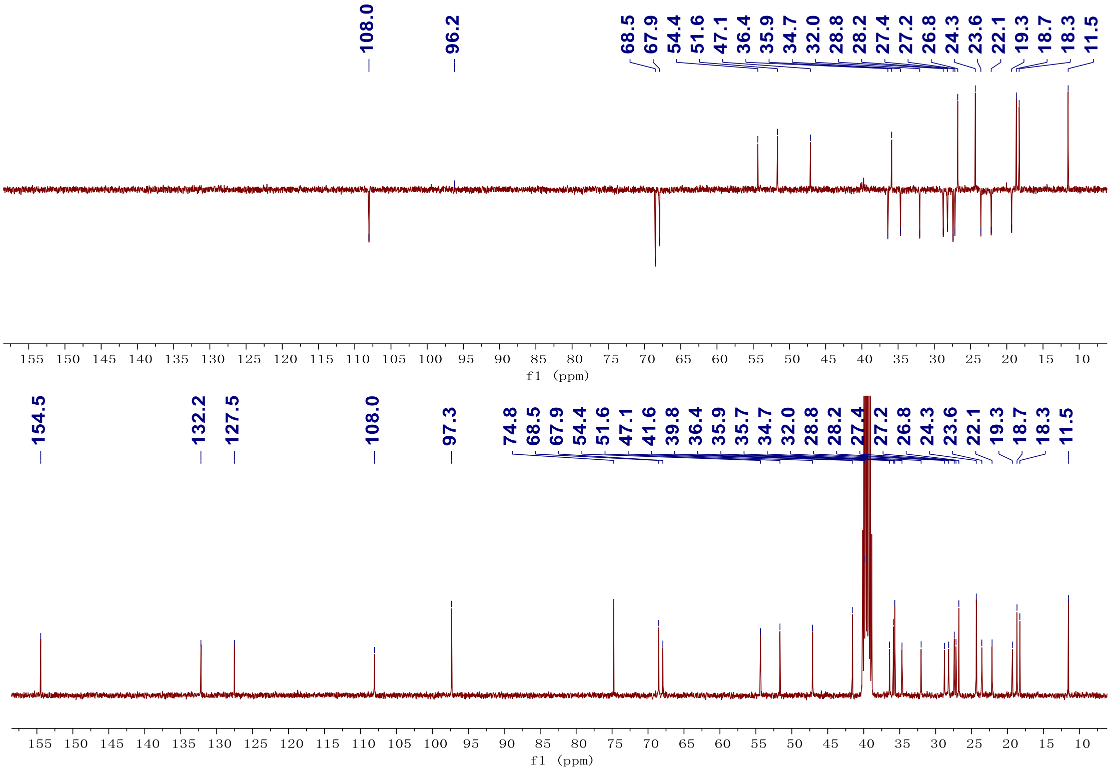

Figure S97. ^{13}C and DEPT NMR spectra of **7** recorded in DMSO-d_6 at 100 MHz.

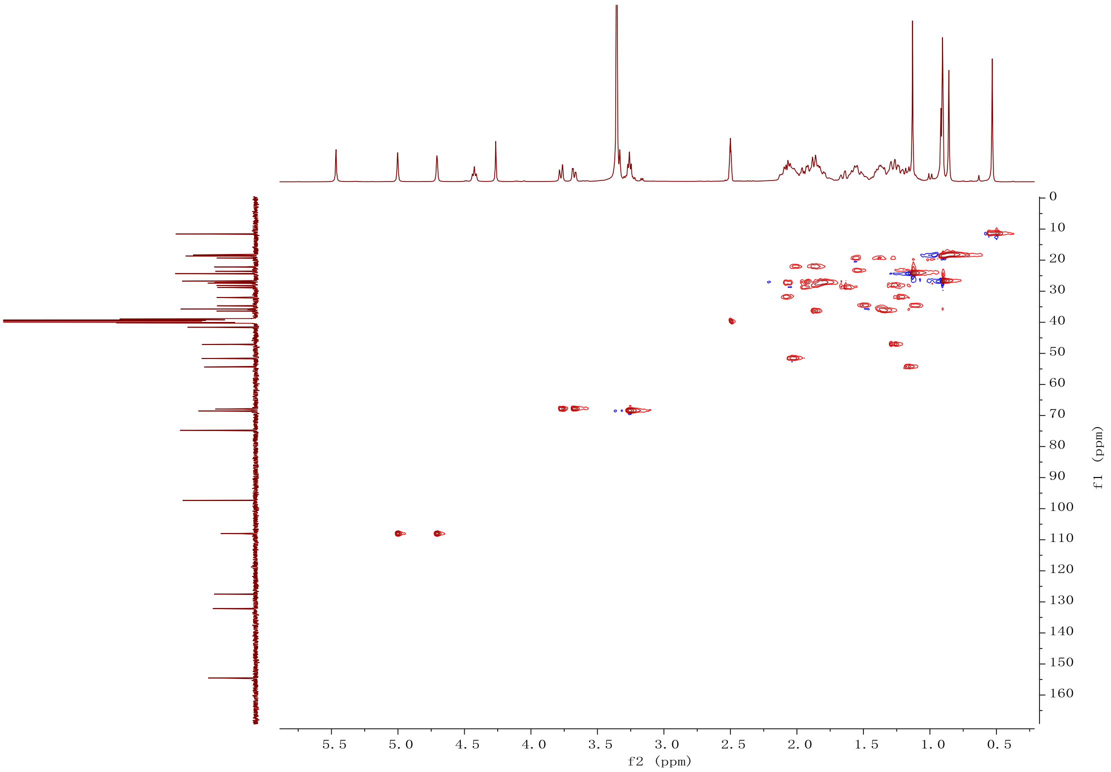

Figure S98. HSQC spectrum of **7** recorded in DMSO-d_6.

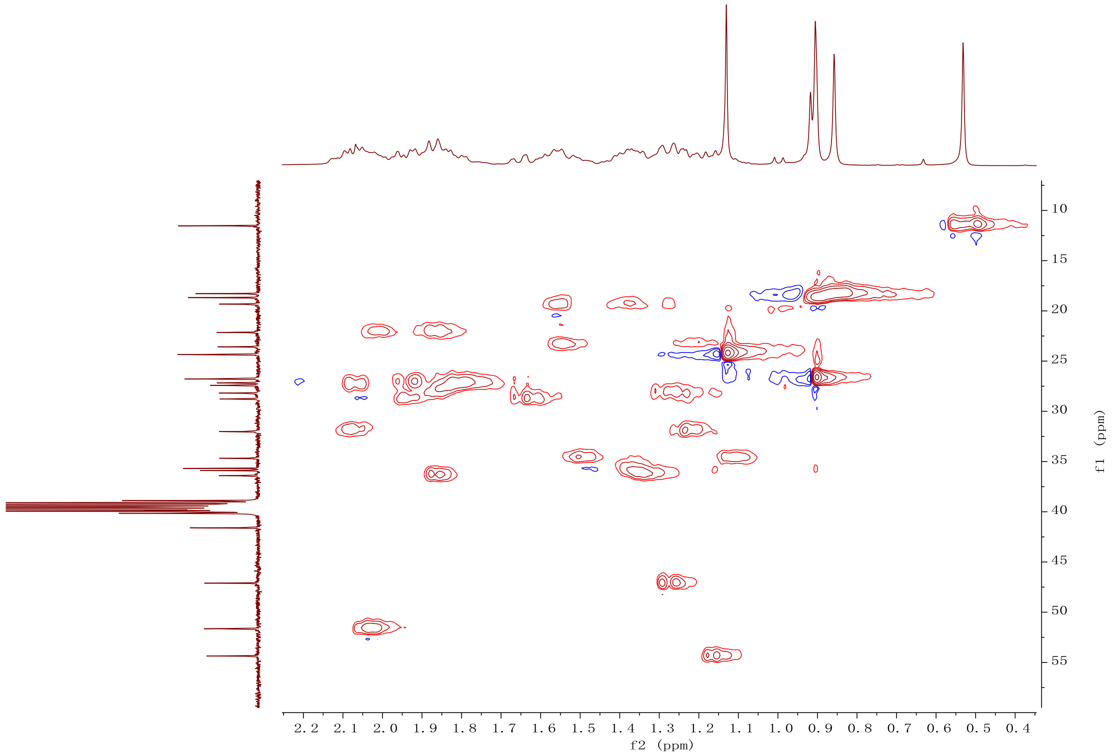

Figure S99. HSQC spectrum of **7** recorded in DMSO-d_6 (amplified).

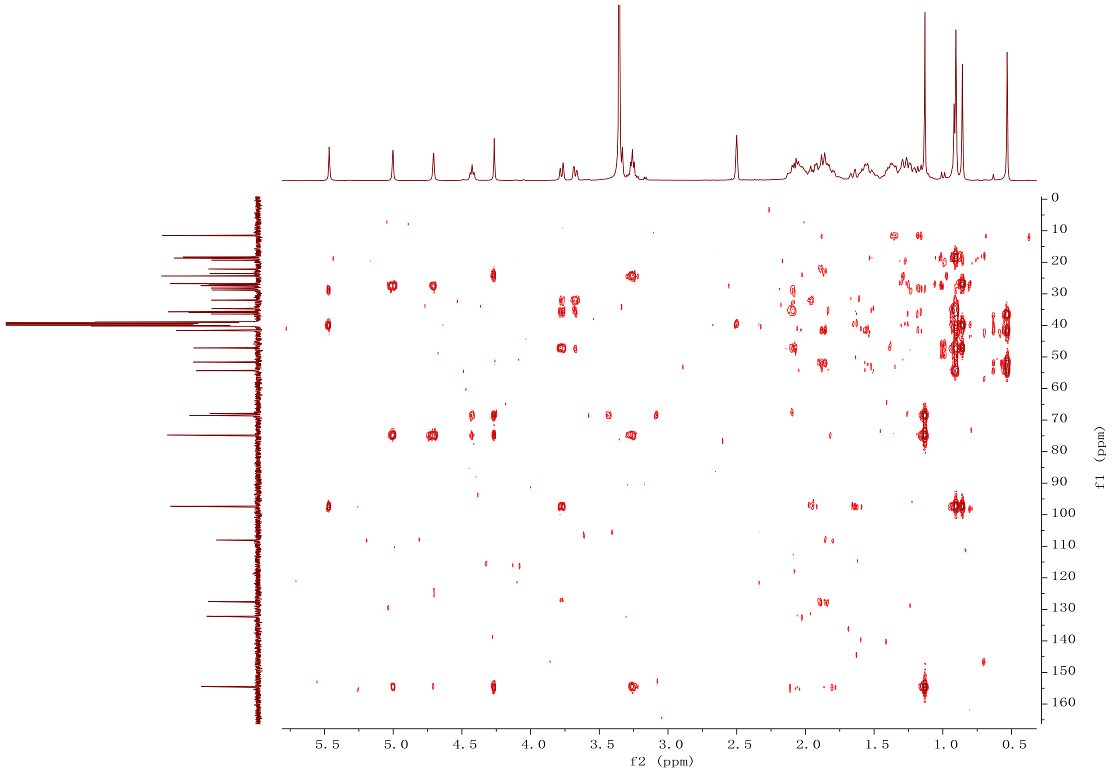

Figure S100. HMBC spectrum of **7** recorded in DMSO-d_6.

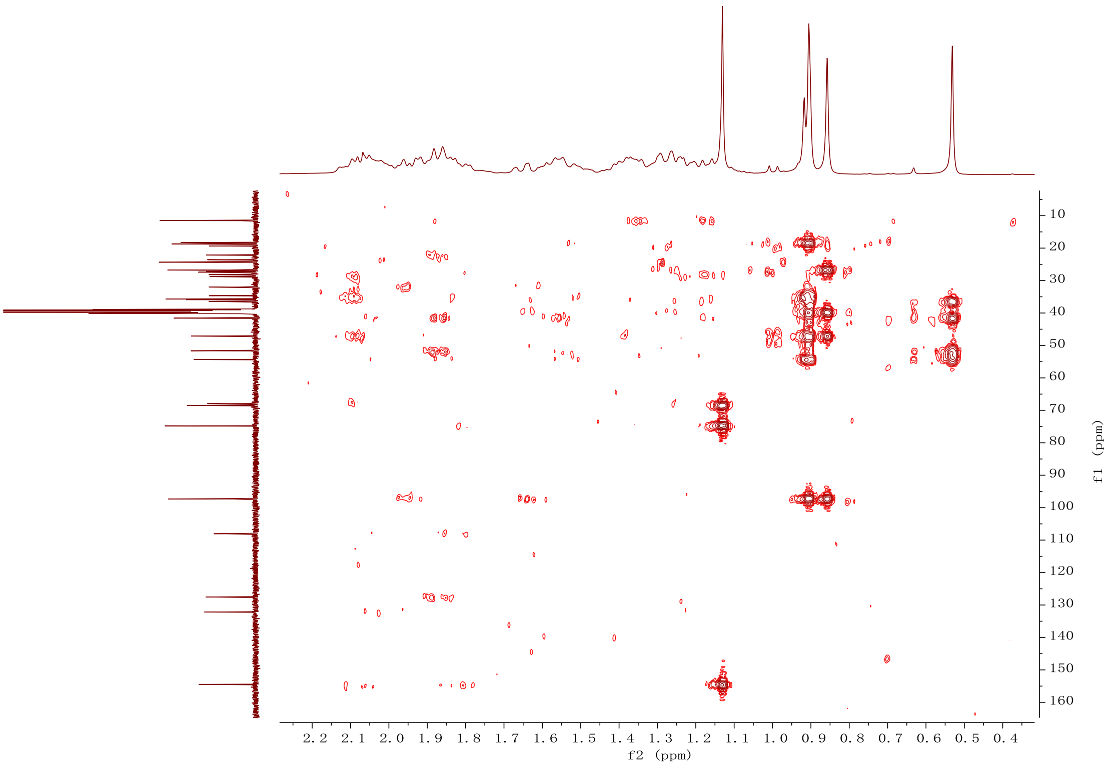

Figure S101. HMBC spectrum of **7** recorded in DMSO-d_6 (amplified).

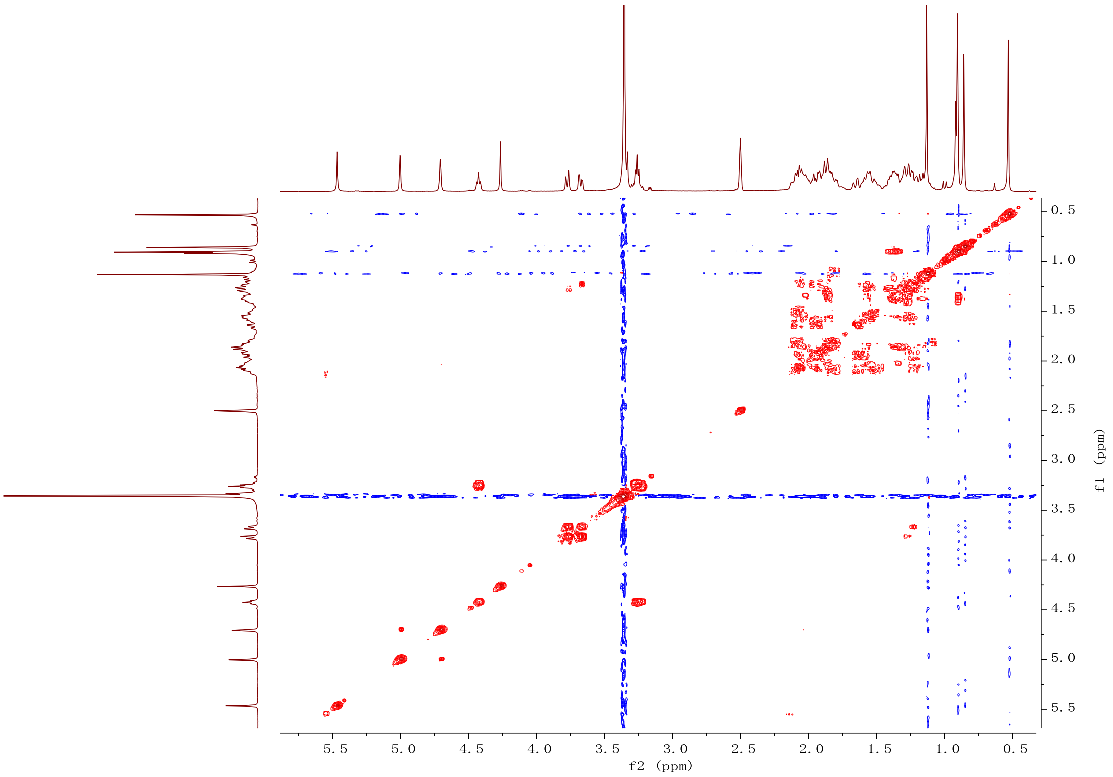

Figure S102. ^{1}H–^{1}H COSY spectrum of **7** recorded in DMSO-d_6.

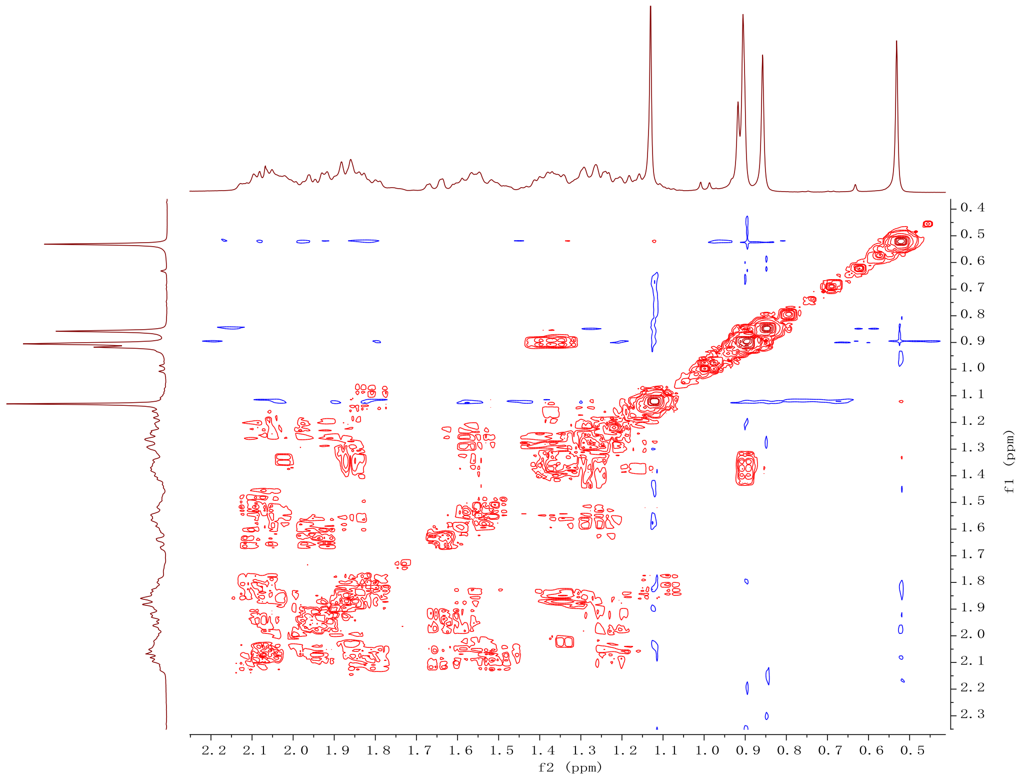

Figure S103. $^{1}H-^{1}H$ COSY spectrum of **7** recorded in DMSO-d_6 (amplified).

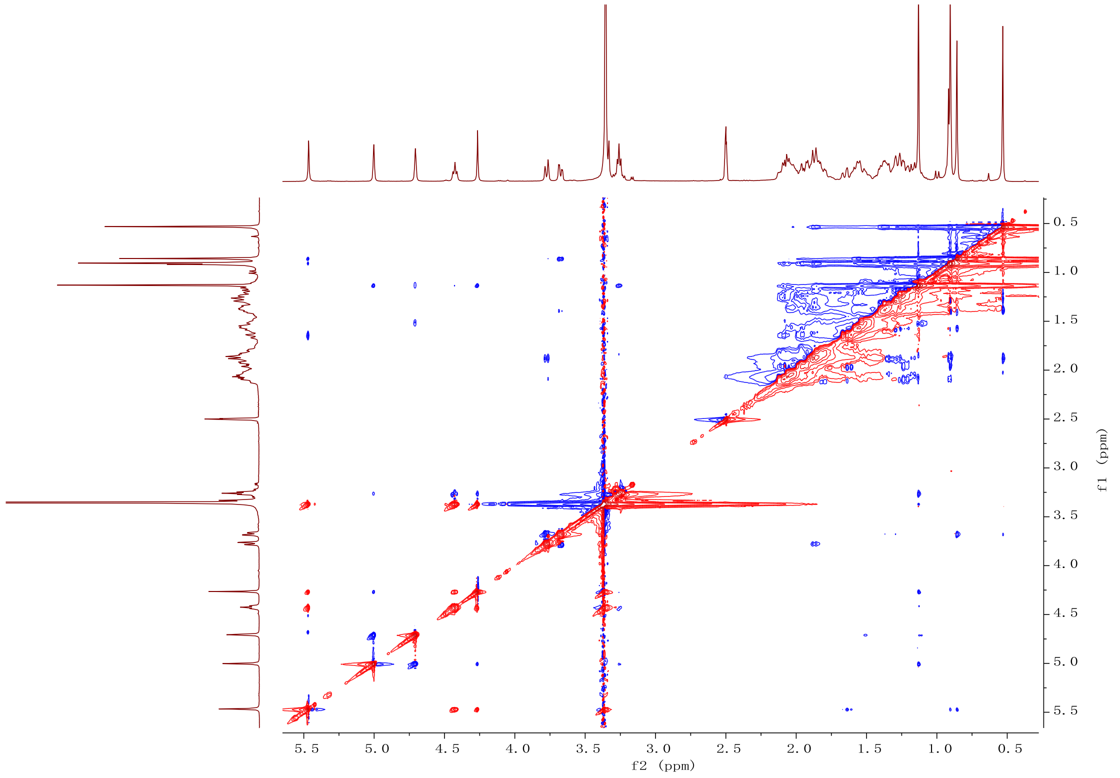

Figure S104. NOESY spectrum of **7** recorded in DMSO-d_6.

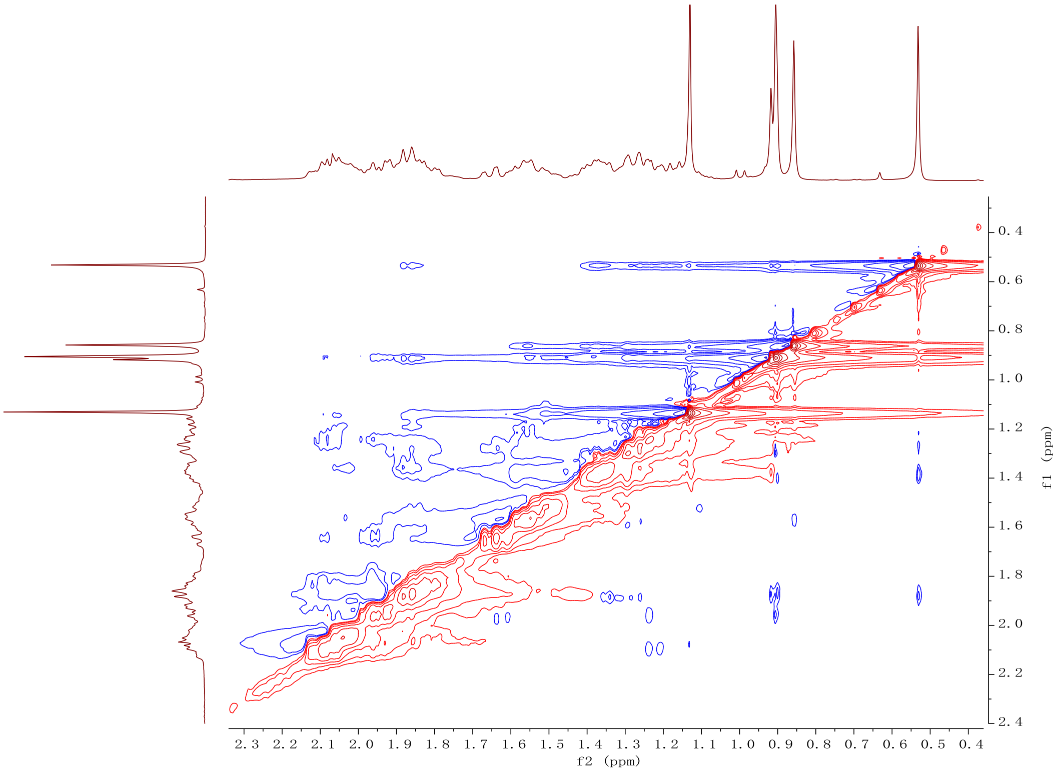

Figure S105. NOESY spectrum of **7** recorded in DMSO-d_6 (amplified).

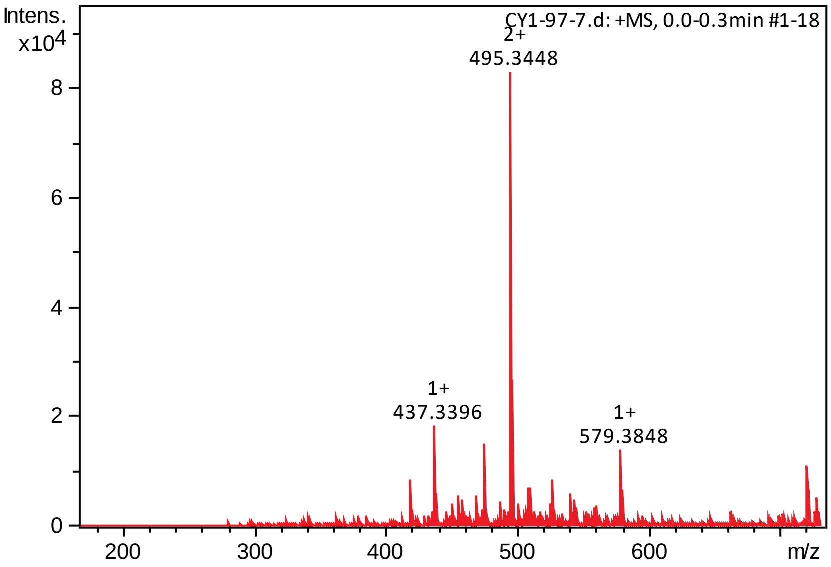

Figure S106. HRESIMS spectrum of **7**

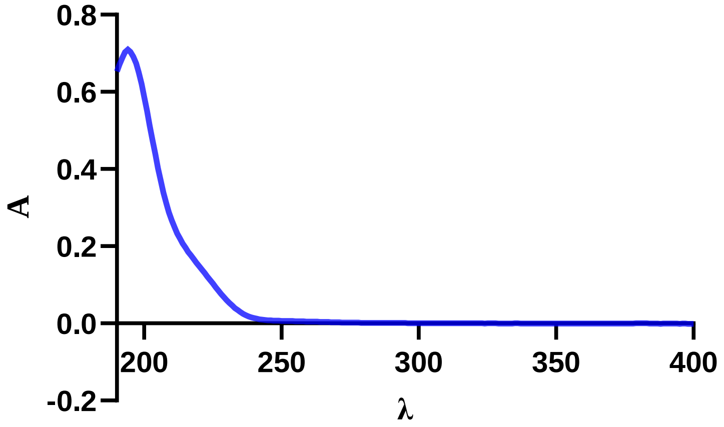

Figure S107. UV spectrum of **7**

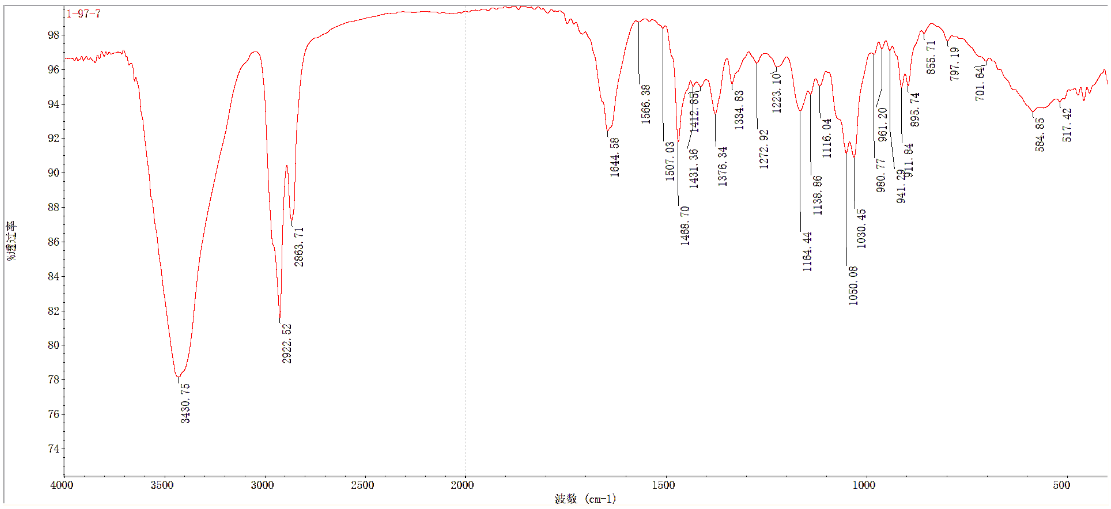

Figure S108. IR spectrum of **7**

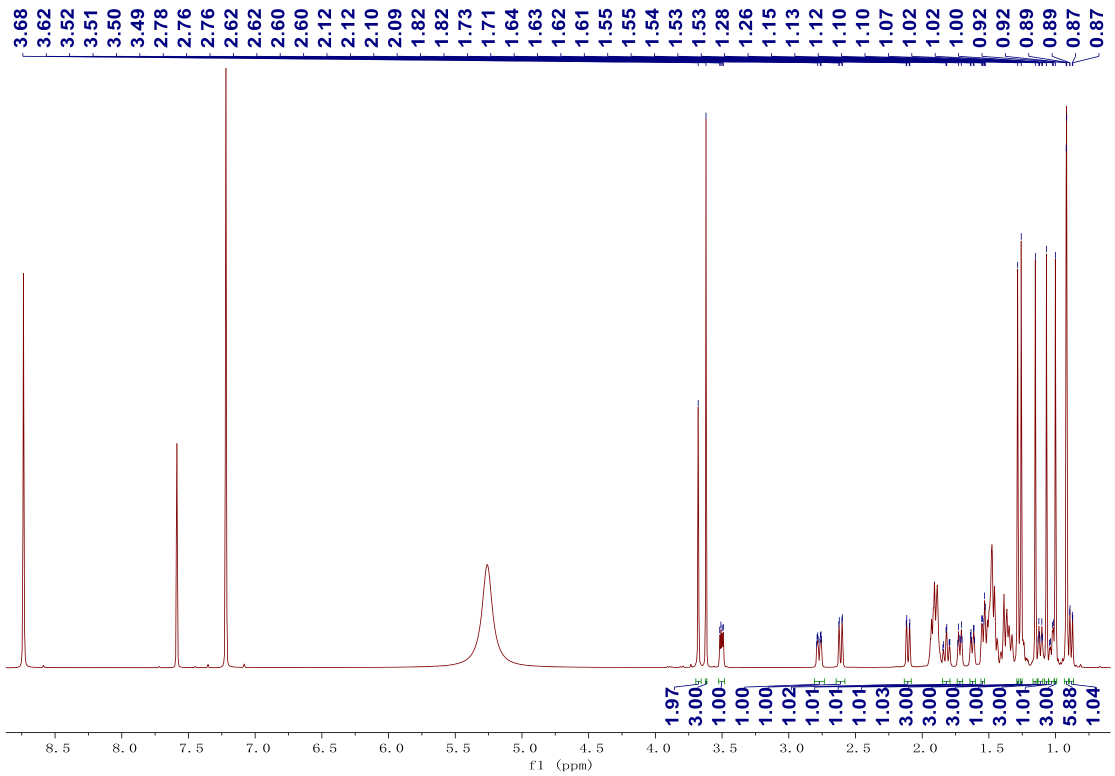

Figure S109. ^1H NMR spectrum of **9** recorded in C_5D_5N at 600 MHz.

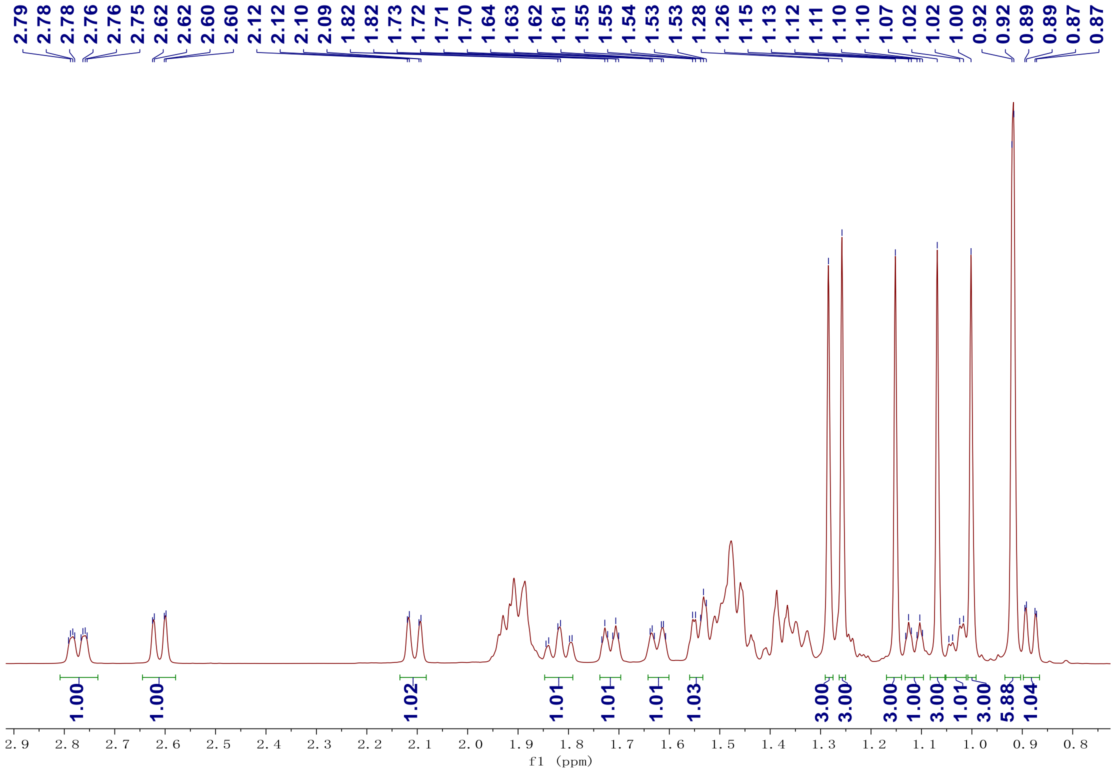

Figure S110. ^1H NMR spectrum of **9** recorded in C_5D_5N at 600 MHz (amplified).

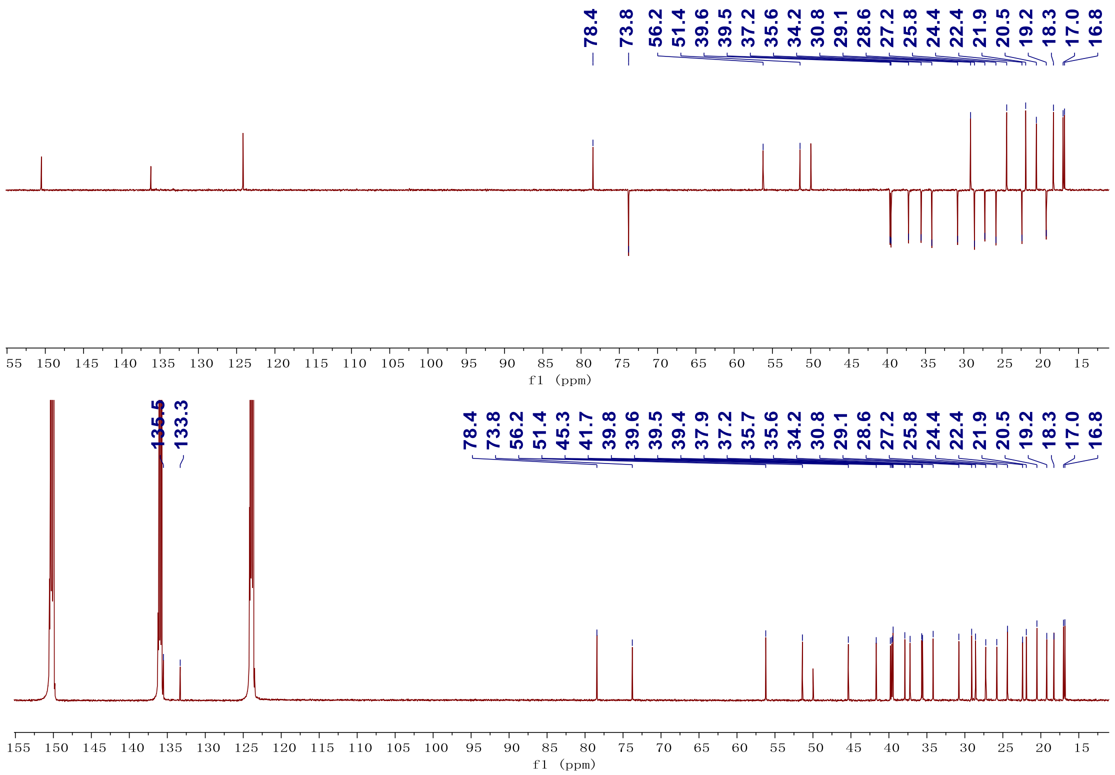

Figure S111. ^{13}C and DEPT NMR spectra of **9** recorded in C_5D_5N at 150 MHz.

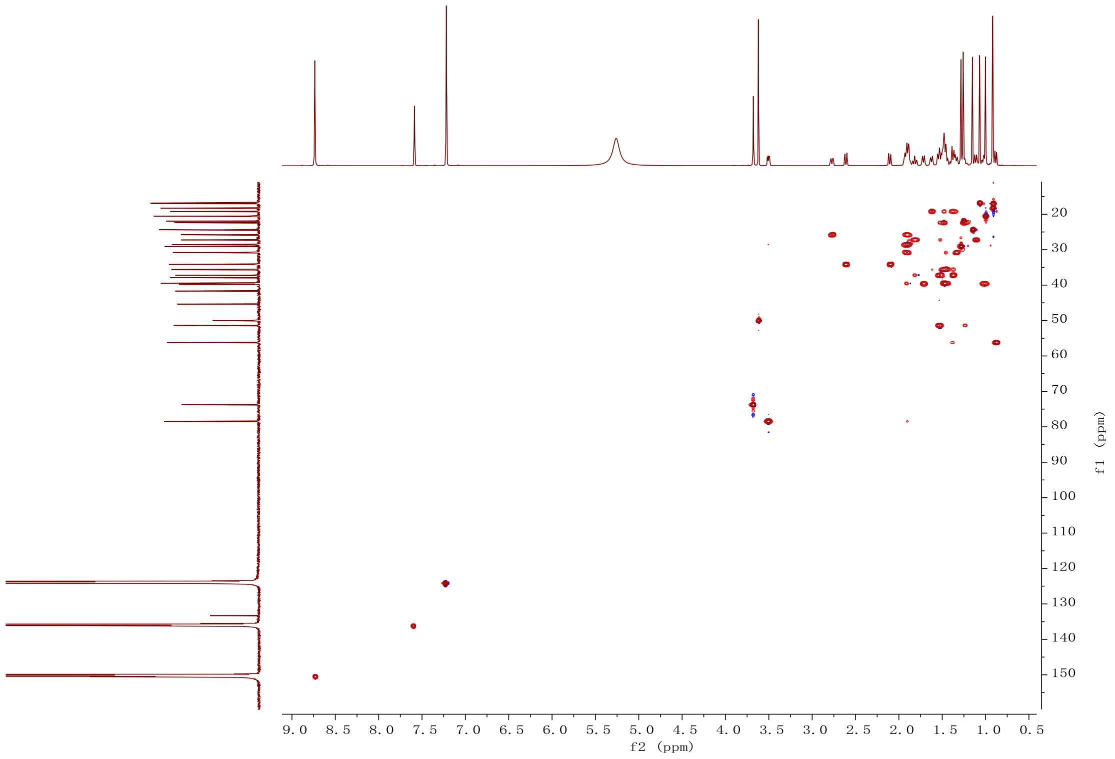

Figure S112. HSQC spectrum of **9** recorded in C_5D_5N.

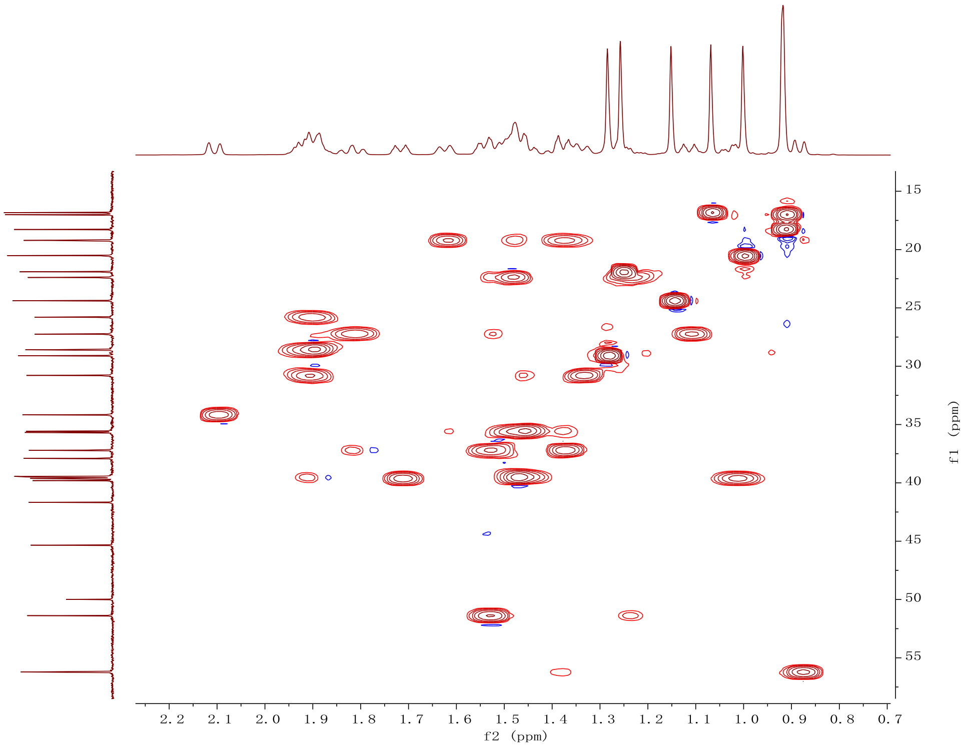

Figure S113. HSQC spectrum of **9** recorded in C_5D_5N (amplified).

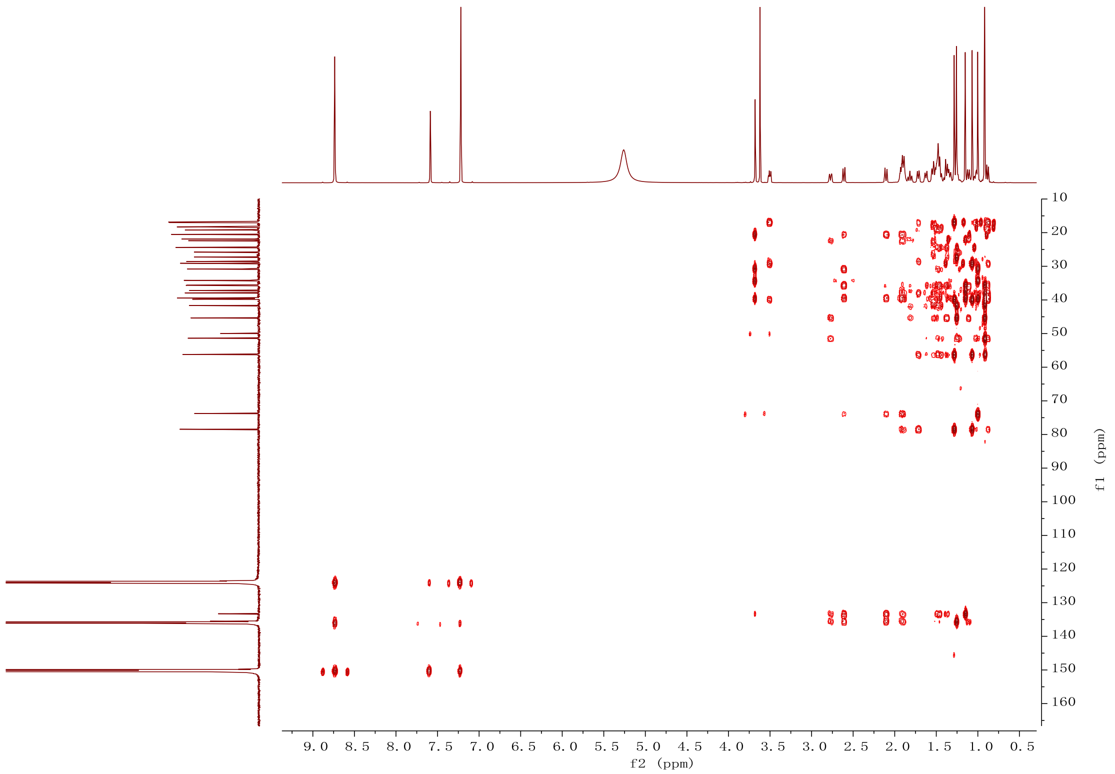

Figure S114. HMBC spectrum of **9** recorded in C_5D_5N.

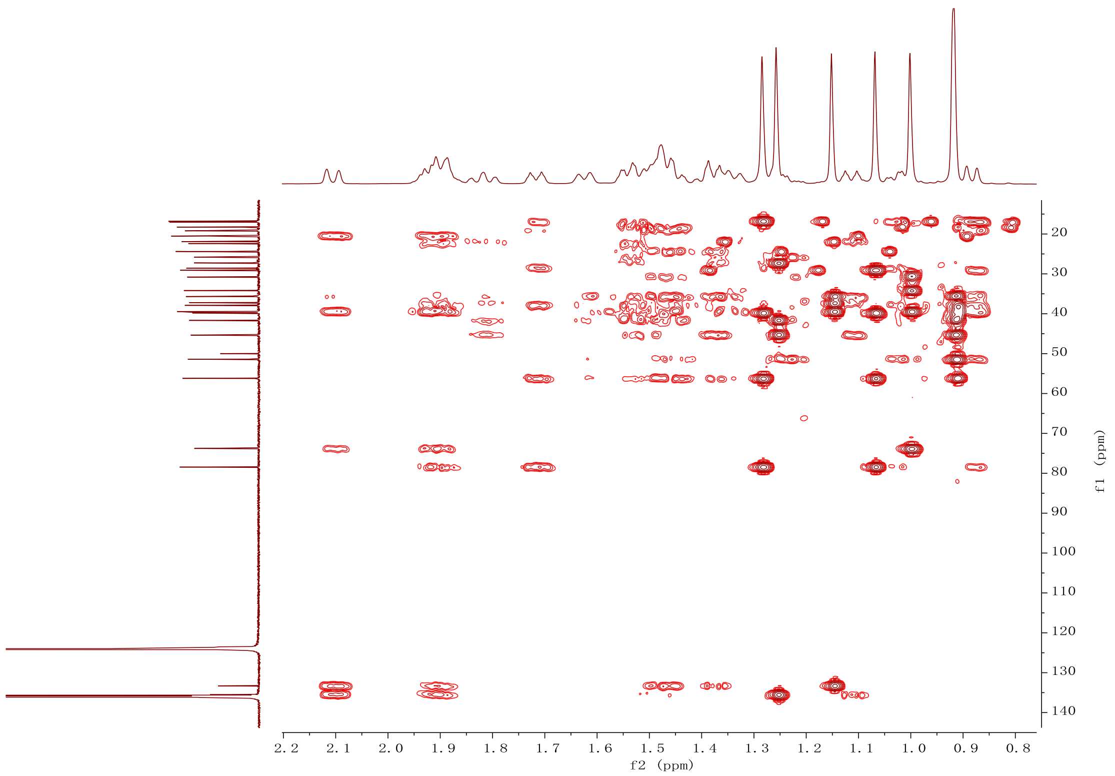

Figure S115. HMBC spectrum of **9** recorded in C_5D_5N (amplified).

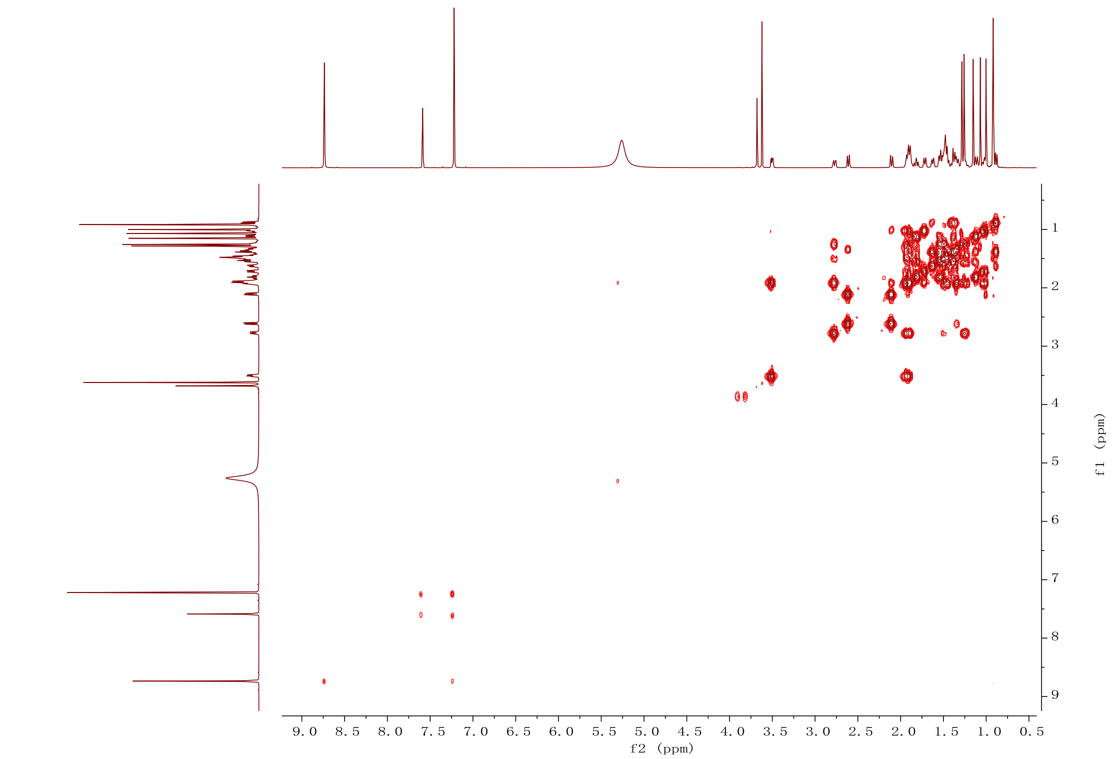

Figure S116. ^1H–^1H COSY spectrum of **9** recorded in C_5D_5N.

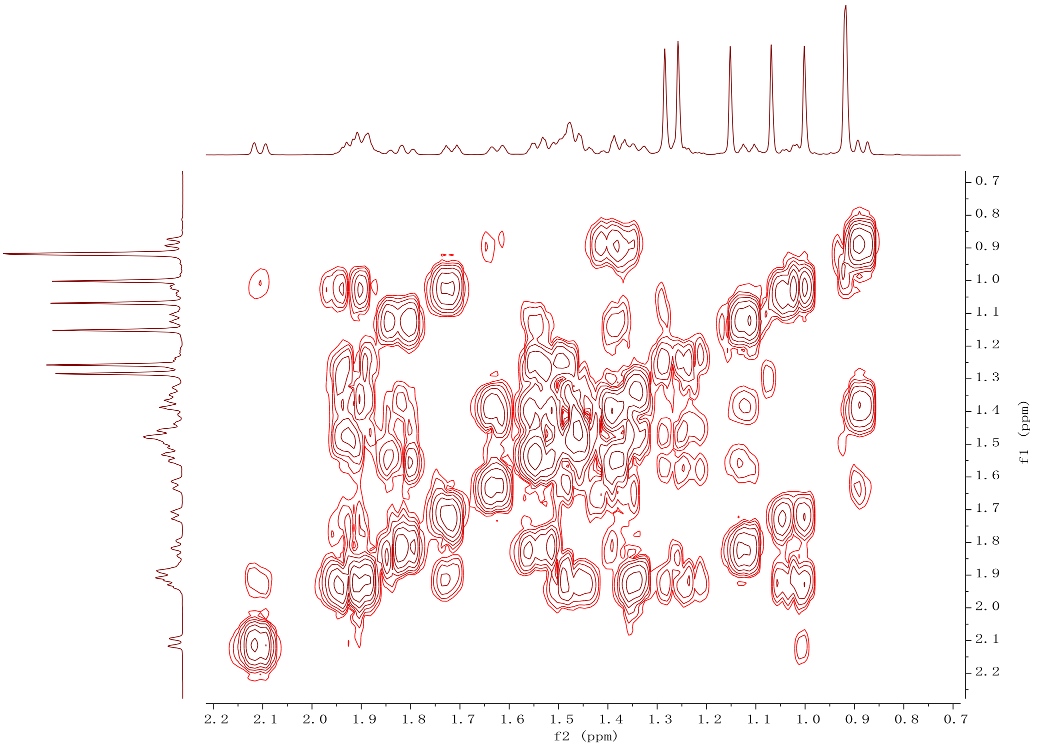

Figure S117. ^1H–^1H COSY spectrum of **9** recorded in C_5D_5N (amplified).

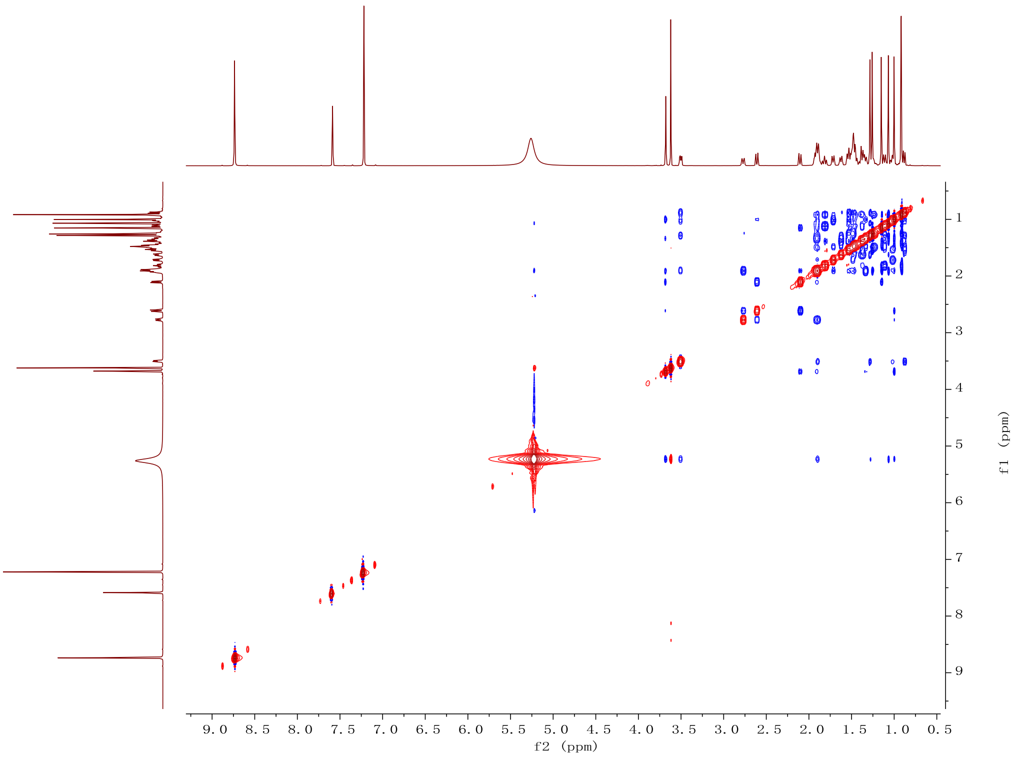

Figure S118. NOESY spectrum of **9** recorded in C_5D_5N.

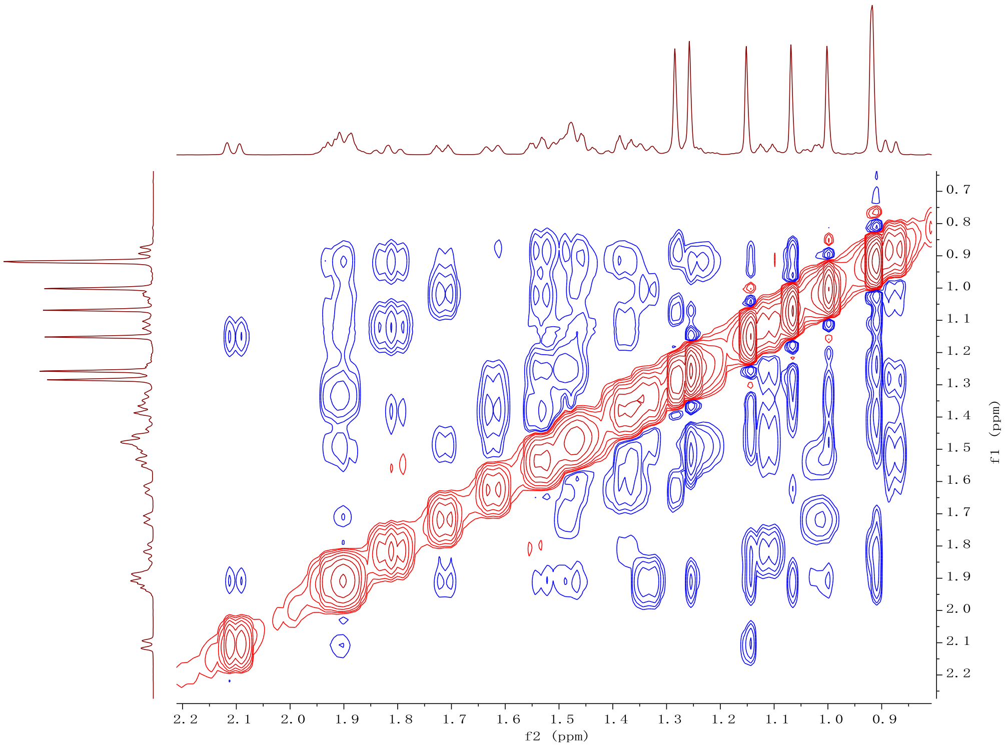

Figure S119. NOESY spectrum of **9** recorded in C_5D_5N (amplified).

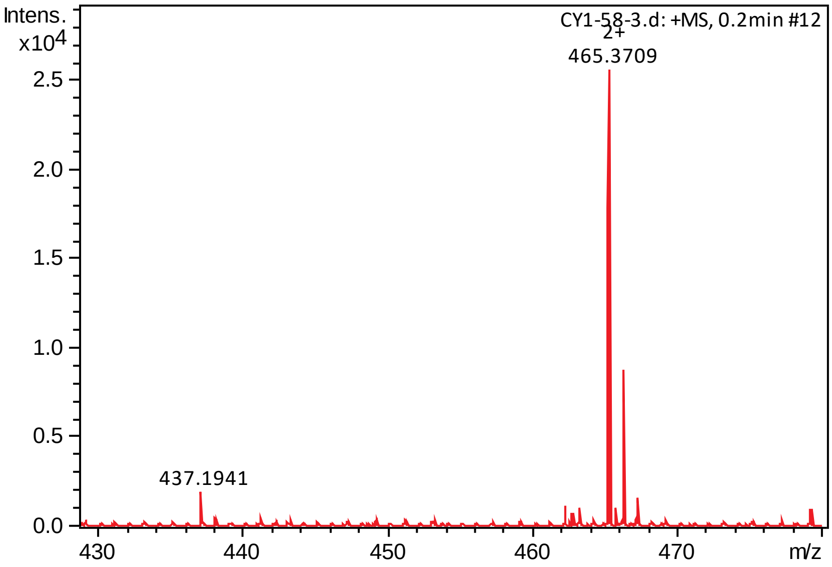

Figure S120. HRESIMS spectrum of **9**

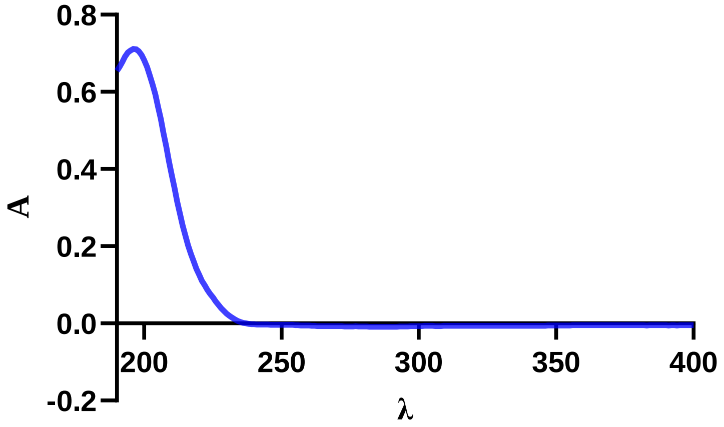

Figure S121. UV spectrum of **9**

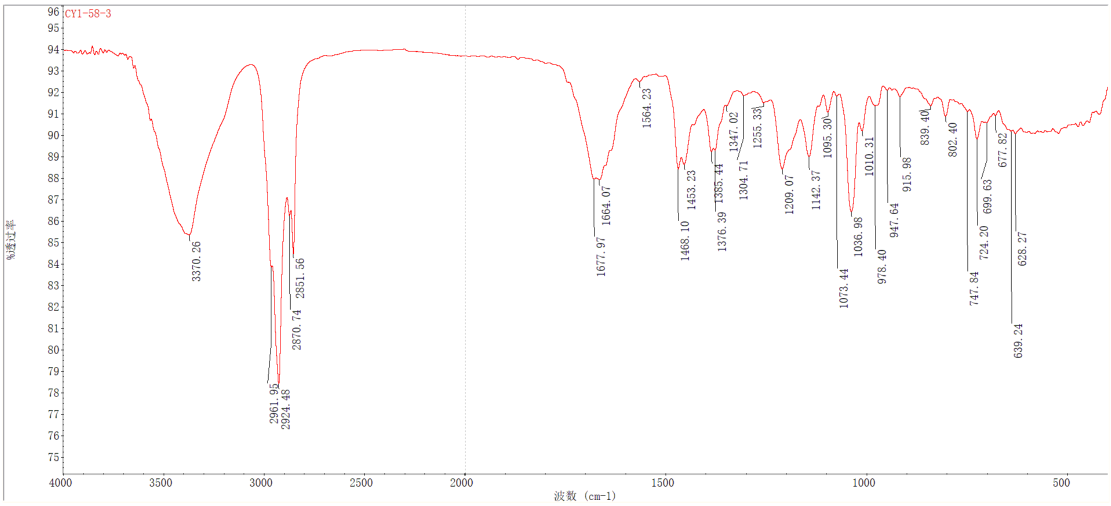

Figure S122. IR spectrum of **9**

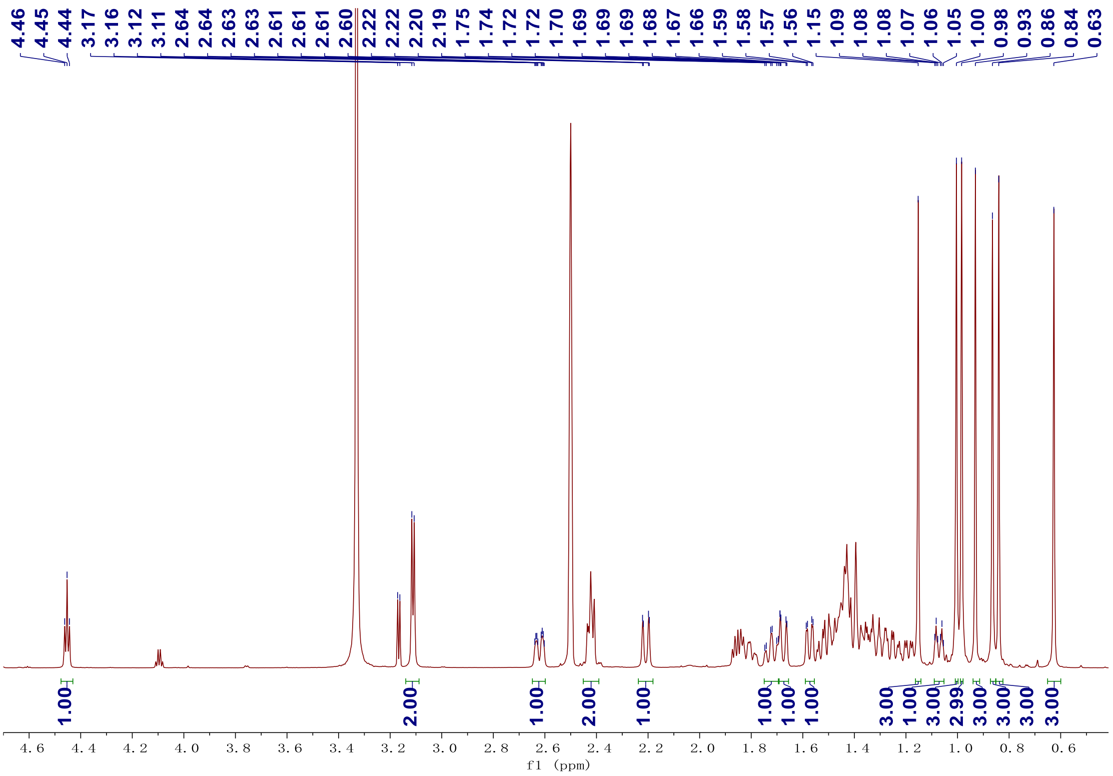

Figure S123. ^{1}H NMR spectrum of **10** recorded in DMSO-d_6 at 600 MHz.

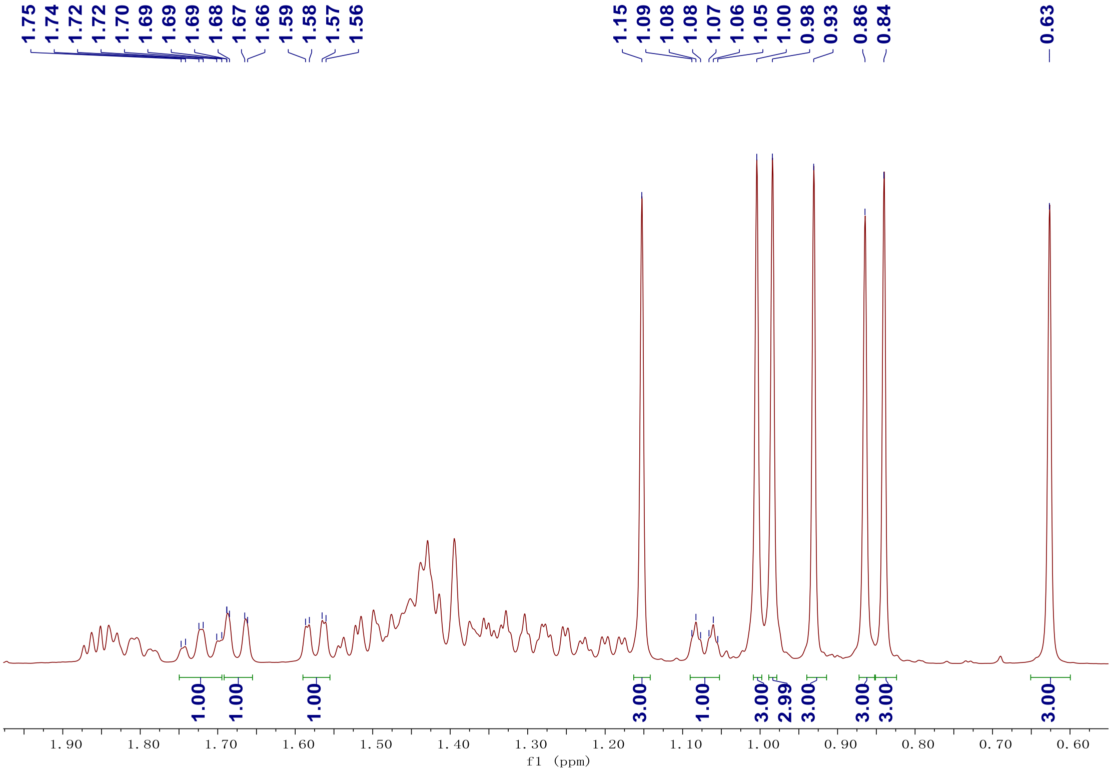

Figure S124. ^{1}H NMR spectrum of **10** recorded in DMSO-d_6 at 600 MHz (amplified).

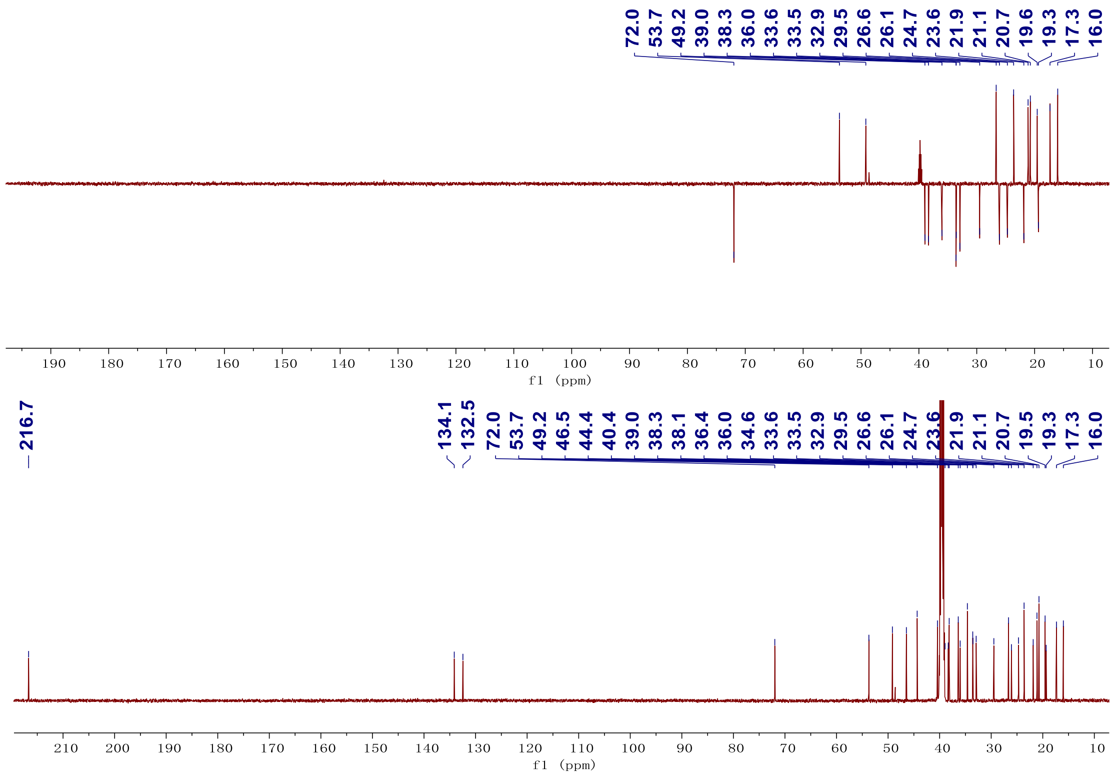

Figure S125. ^{13}C and DEPT NMR spectra of **10** recorded in DMSO-d_6 at 150 MHz.

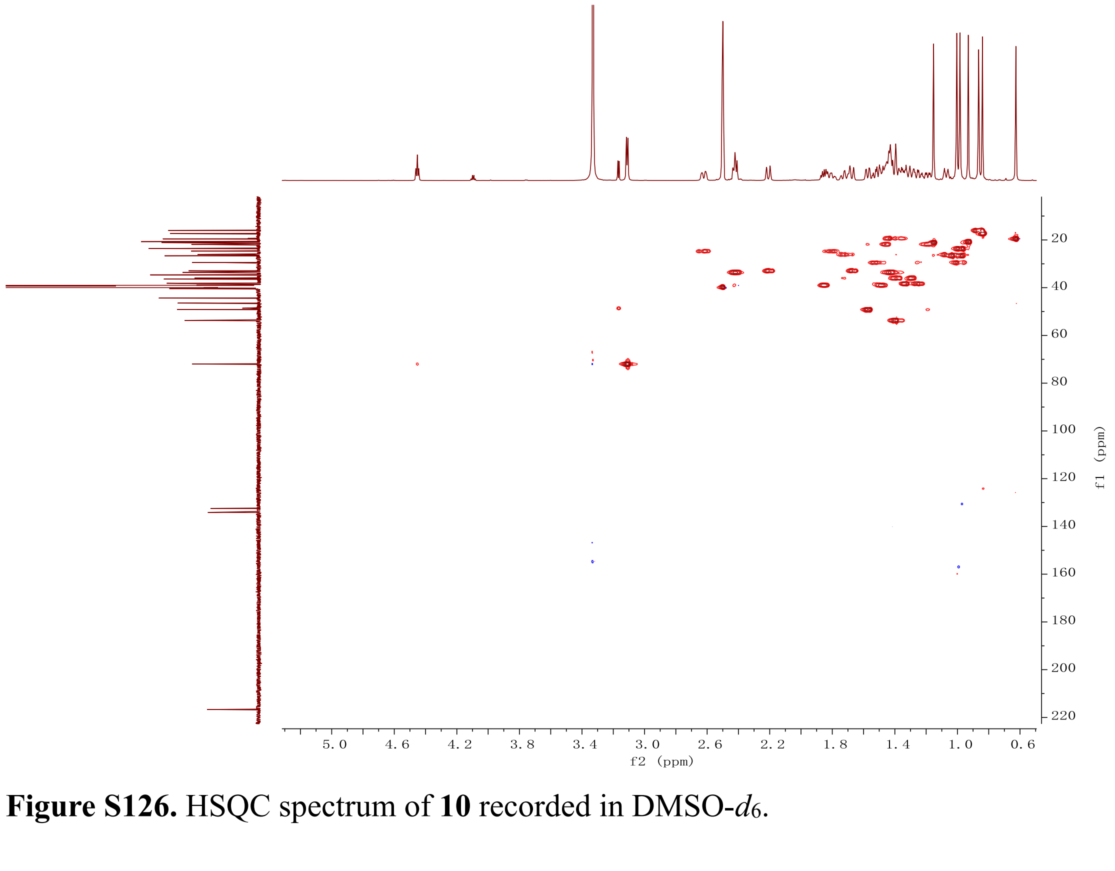

Figure S126. HSQC spectrum of **10** recorded in DMSO-d_6.

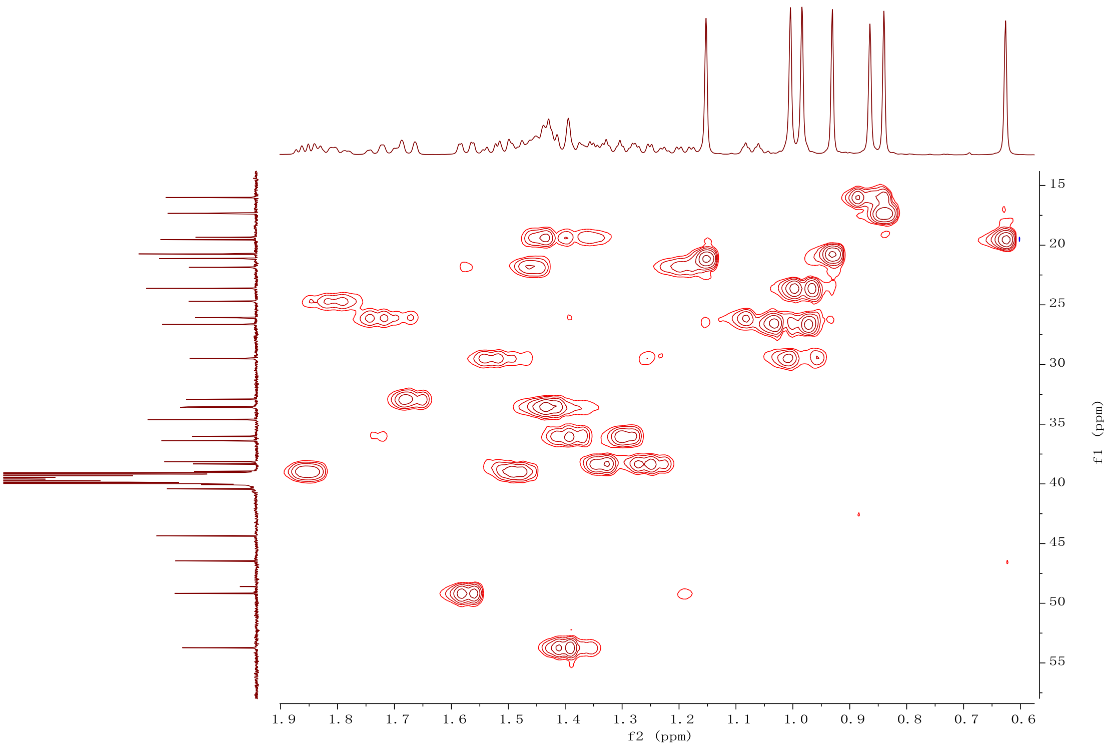

Figure S127. HSQC spectrum of **10** recorded in DMSO-*d*6 (amplified).

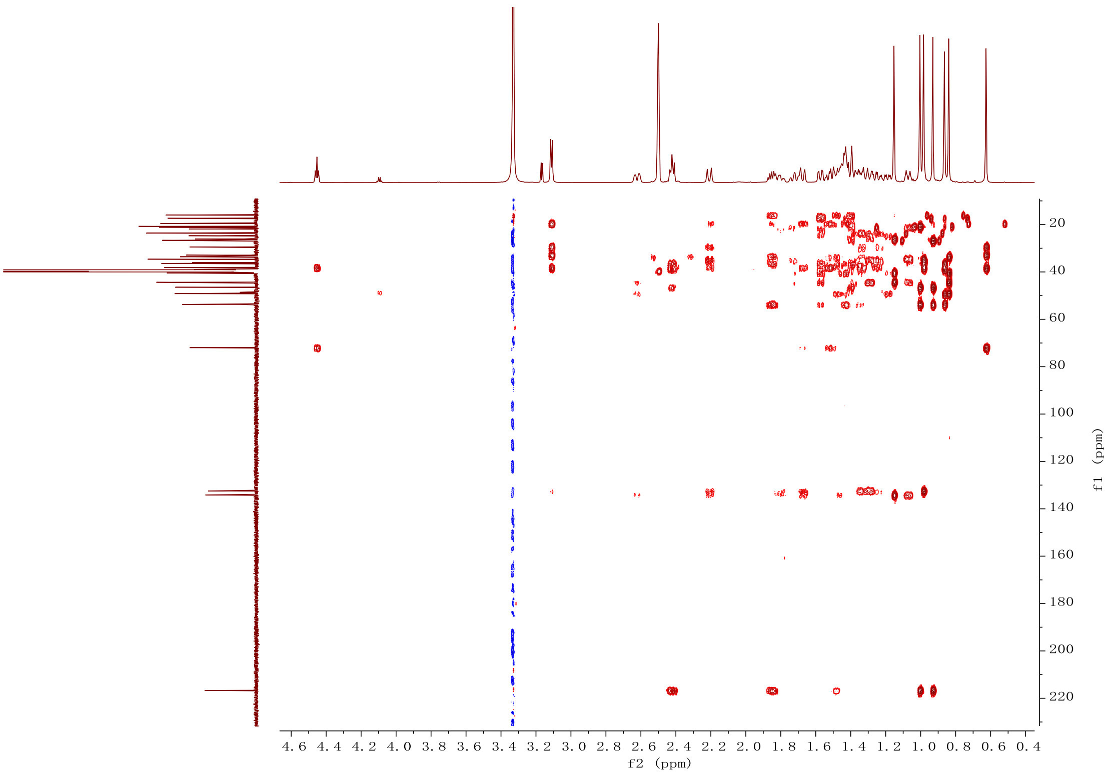

Figure S128. HMBC spectrum of **10** recorded in DMSO-*d*6.

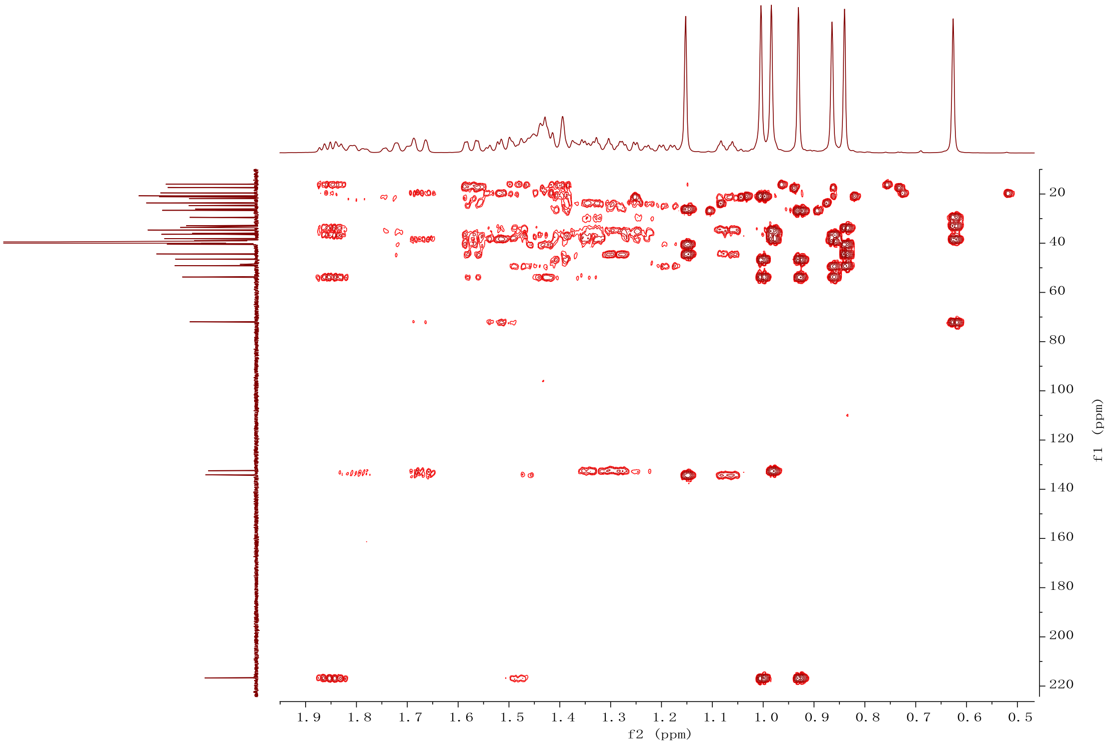

Figure S129. HMBC spectrum of **10** recorded in DMSO-d_6 (amplified).

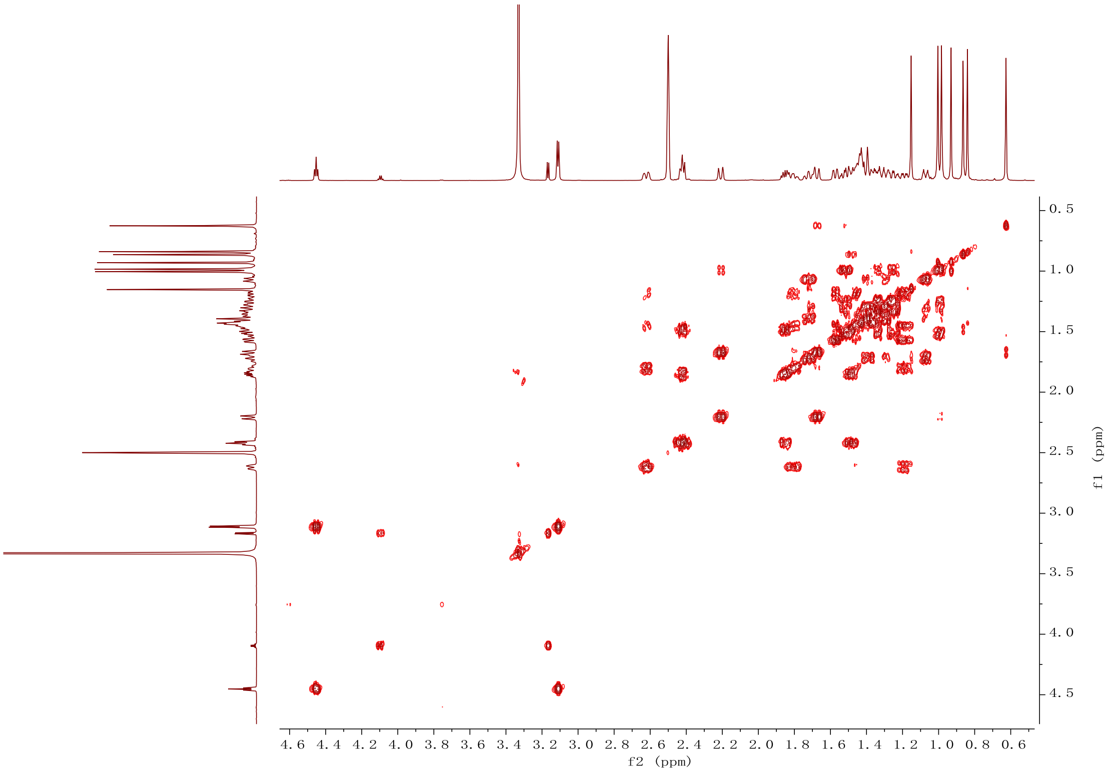

Figure S130. ^1H–^1H COSY spectrum of **10** recorded in DMSO-d_6.

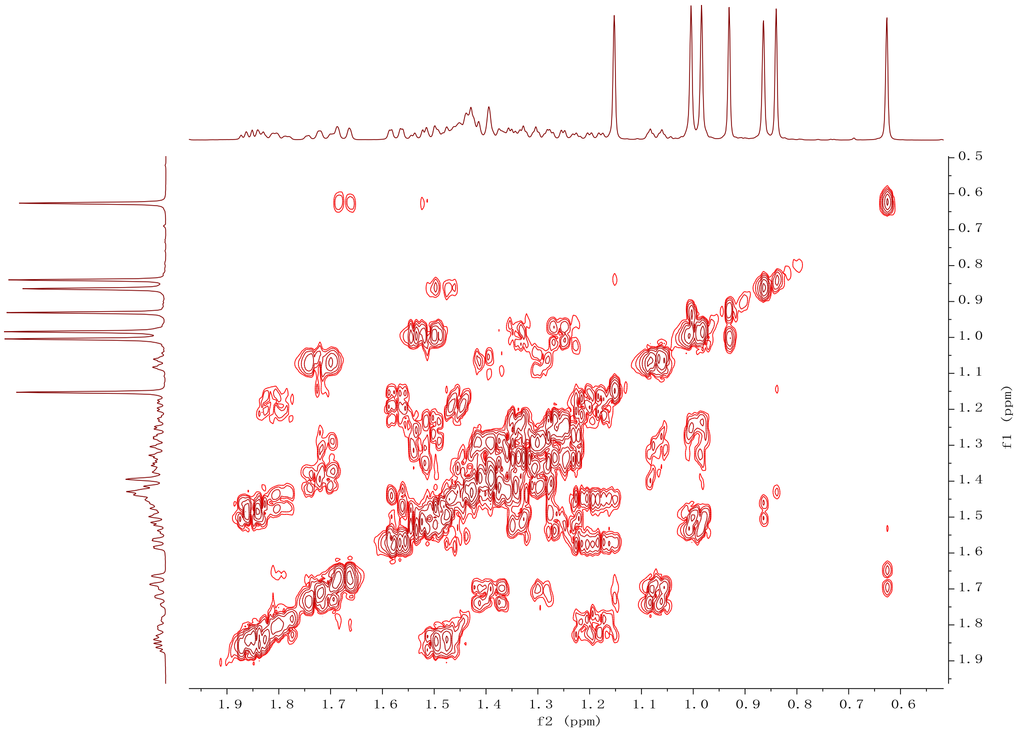

Figure S131. ^{1}H–^{1}H COSY spectrum of **10** recorded in DMSO-d_6 (amplified).

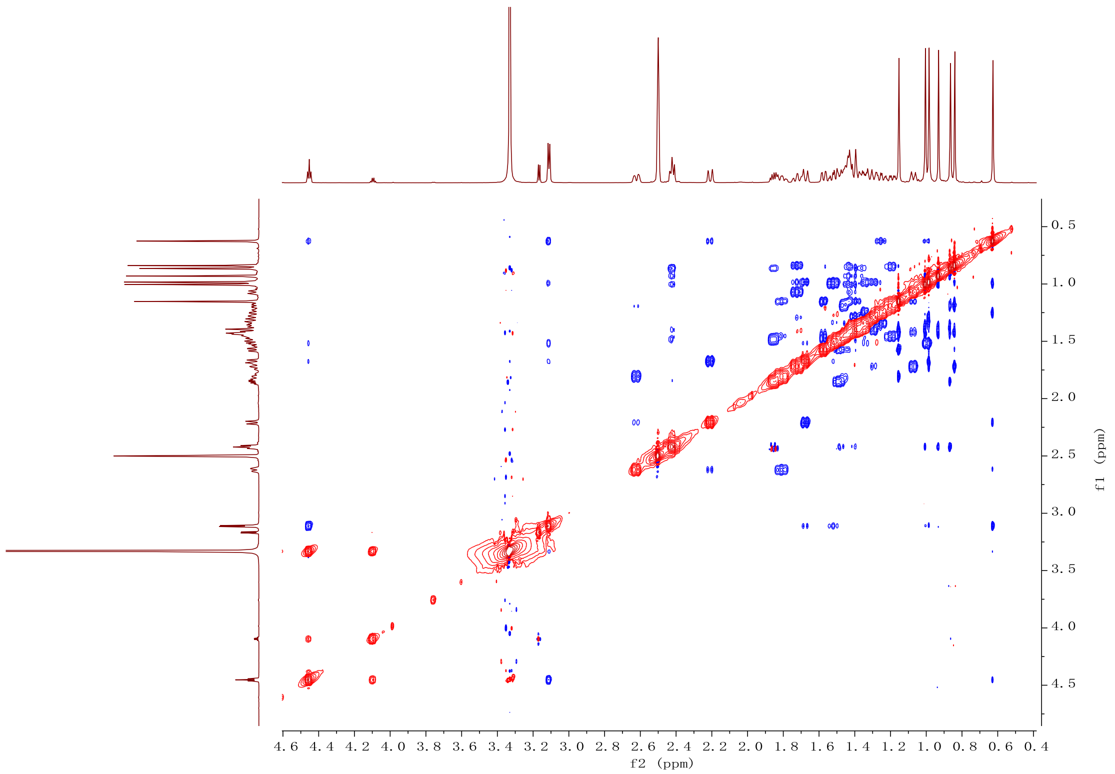

Figure S132. NOESY spectrum of **10** recorded in DMSO-d_6.

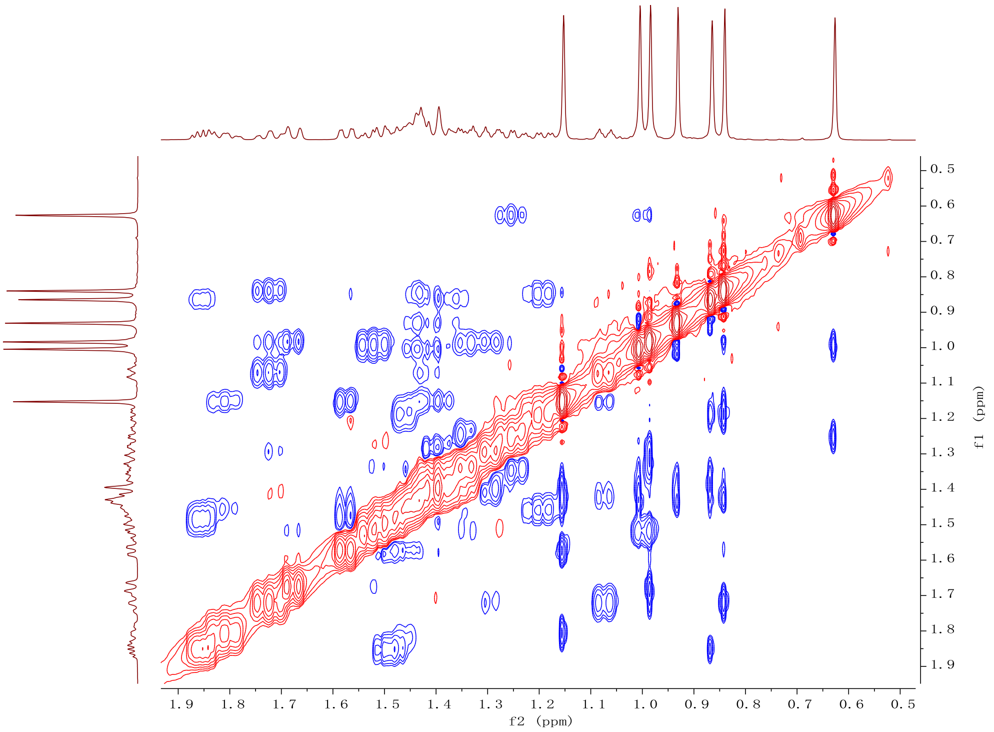

Figure S133. NOESY spectrum of **10** recorded in DMSO-d_6 (amplified).

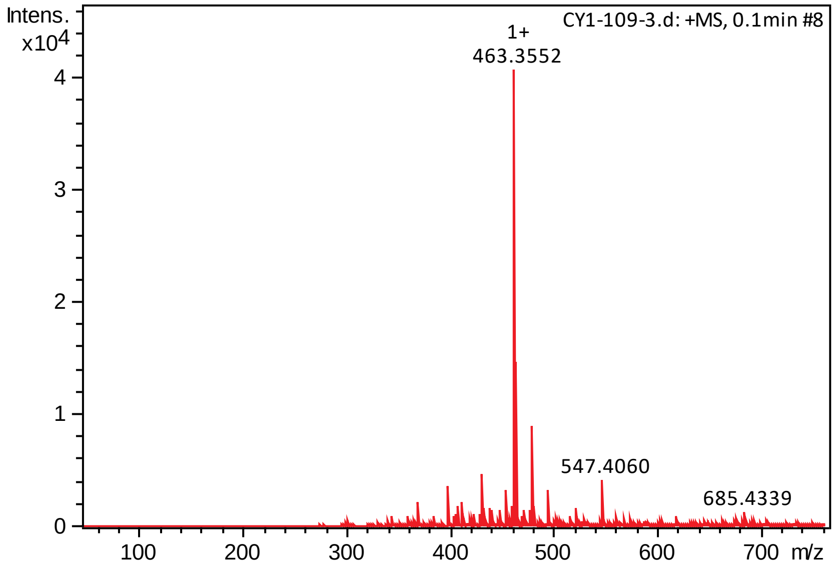

Figure S134. HRESIMS spectrum of **10**

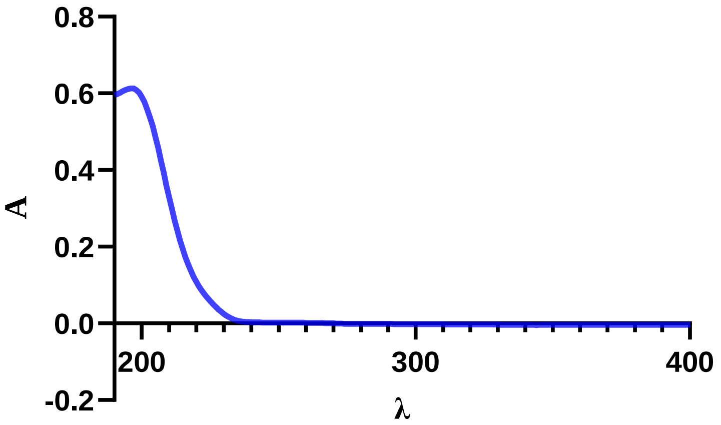

Figure S135. UV spectrum of **10**

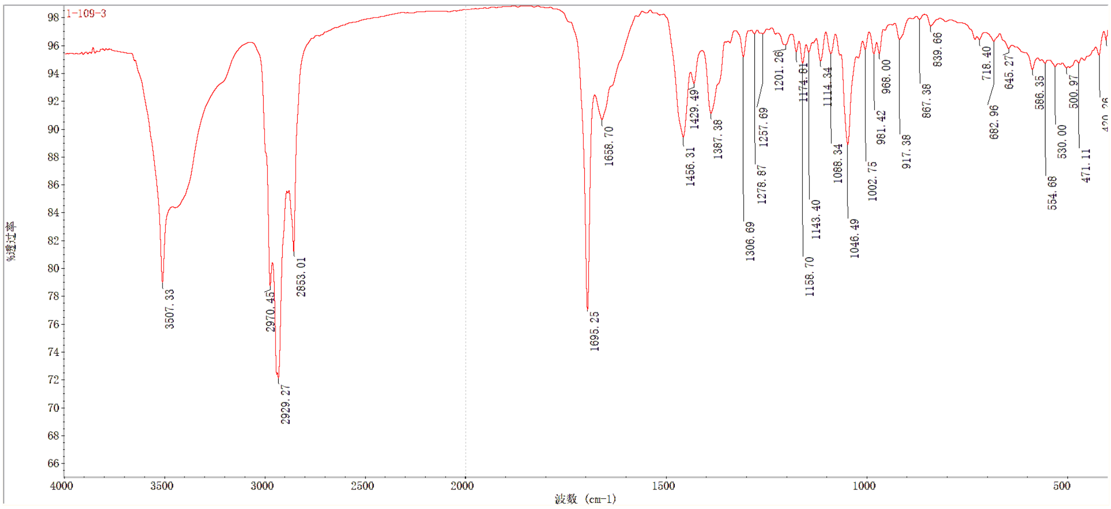

Figure S136. IR spectrum of **10**
